# Identification of novel homologous microRNA genes in the rhesus macaque genome

**DOI:** 10.1186/1471-2164-9-8

**Published:** 2008-01-10

**Authors:** Junming Yue, Yi Sheng, Kyle E Orwig

**Affiliations:** 1 Department of Obstetrics, Gynecology and Reproductive Sciences, University of Pittsburgh School of Medicine, Pittsburgh, PA, 15213, USA; 2 Magee-Womens Research Institute and Foundation, Pittsburgh, PA, 15213, USA

## Abstract

**Background:**

MicroRNAs (miRNAs) are about 22 nucleotide (nt) endogenous small RNAs that negatively regulate gene expression. They are a recently described class of regulatory molecules that has biological implications for tumorigenesis, development, metabolism and viral diseases. To date, 533 miRNAs have been identified in human. However, only 71 miRNAs have been reported in rhesus macaque. The rhesus is widely used in medical research because of its genetic and physiological similarity to human. The rhesus shares approximately 93% similarity with human in genome sequences and miRNA genes are evolutionarily conserved. Therefore, we searched the rhesus genome for sequences similar to human miRNA precursor sequences to identify putative rhesus miRNA genes.

**Results:**

In addition to 71 miRNAs previously reported, we identified 383 novel miRNA genes in the rhesus genome. We compared the total 454 miRNAs identified so far in rhesus to human homologs, 173 miRNA genes showed 100% homology in precursor sequences between rhesus and human; The remaining 281 show more than 90%, less than 100% homology in precursor sequences. Some miRNAs in the rhesus genome are present as clusters similar to human, such as miR-371/373, miR-367/302b, miR-17/92, or have multiple copies distributed in the same or different chromosomes. RT-PCR analysis of expression of eight rhesus miRNA genes in rhesus tissues demonstrated tissue-specific regulation of expression.

**Conclusion:**

Identification of miRNA genes in rhesus will provide the resources for analysis of expression profiles in various tissues by creating a rhesus miRNA array, which is currently not available for this species. Investigation of rhesus miRNAs will also expand our understanding of their biological function through miRNA knockout, knockdown or overexpression.

## Background

MicroRNAs (miRNAs) are non-coding small endogenous RNAs with a length of about 22 nt that negatively regulate gene expression by degrading mRNA or impeding protein translation [1]. MiRNA genes are hosted in intronic, exonic or intergenic regions of the genome and are transcribed into primary miRNA (pri-miRNA) by polymerase II. The pri-miRNAs are processed into ~70 nt pre-miRNAs with a hairpin structure by a microprocessor complex composed of Drosha and Pasha [2-4]. The pre-miRNAs are then transported into the cytoplasm by exportin-5, where the RNAase III enzyme, Dicer, cleaves pre-miRNA into ~22 nt mature miRNAs, which are recruited into the RNA induced silencing complex (RISC) localized in discrete cytoplasmic foci, P bodies [5-8]. The RISC targets the mRNAs by perfect match to degenerate the target transcripts or binds the 3'UTR through imperfect base-pairing to block protein translation [9]. Recently computational analysis suggests miRNA may also bind 5'UTR [10]. One miRNA may target more than a hundred genes [10]. The discovery of miRNAs has led us to rethink the conventional mechanisms of gene regulation, and current research is focused on understanding how these small molecules function in biological processes.

Experimental evidence reveals that miRNAs play important roles in a variety of diseases, such as cancer, diabetes, viral infection, cardiac diseases, as well as in stem cell biology [11-18]. Some miRNAs are present in the genome as clusters where multiple miRNAs are aligned in the same orientation and transcribed as a polycistronic structure, which may function synchronously and cooperatively. The human miR-17/92 cluster composed of 5 miRNAs (miR-17, 18, 19, 20, 92) was found to be related to tumorigenesis and promoted tumor angiogenesis through targeting the anti-angiogenic thrombospondin-1 (Tsp1) by miR-19 or connective tissue growth factor (CTGF) by miR-18 to down-regulate their functions [19,20]. The cluster miR371/373, which is highly expressed in human testicular germ cell tumors, has been demonstrated to function as an oncogene and is capable of overcoming Ras-mediated senescence in human primary fibroblasts [21]. In undifferentiated human ES cells, this cluster is also highly expressed and down-regulated upon differentiation, implicating a role in regulating stem cell self-renewal and differentiation [18].

To date, 533 human miRNAs have been discovered, and the functions of some of them have been experimentally verified [22]. While rhesus is an outstanding model of human physiology, only 71 rhesus miRNAs have been reported and registered in the **Wellcome Trust Sanger Institute miRBase **[23-25]. The study of miRNA in this species lags far behind the mouse, rat and human as well as invertebrates and plants. There are no reports on miRNA functions in rhesus thus far. Recently, the whole rhesus genome was sequenced through an international collaboration, which provides an opportunity to dissect the genome and identify miRNA genes [26].

To facilitate the examination of miRNAs in rhesus, we used human pre-miRNA sequences to query the rhesus genomic database at **UCSC Genome Bioinformatics **[27-29] for homologous rhesus sequences to predict potential miRNA genes. This approach is feasible because miRNA genes are evolutionarily conserved and the human and rhesus genomes share about 93% identity [26,30].

## Results

### MiRNA genes in the rhesus genome

We searched the rhesus genome for potential miRNA genes orthologous to published human precursor miRNA sequences in the **Wellcome Trust Sanger Institute miRBase **[23]. In addition to homology with human miRNA sequences, the search criteria required that miRNA genes encode mature miRNAs with at least 16 base pairings in the stem of the hairpin and a low calculated free energy (-25 k cal/mol) to form a hairpin structure [31,32]. Based on these criteria, we identified 454 rhesus miRNA genes, including 383 novel miRNA genes and 71 that were previously reported [33] (see additional file 1, Table 1). Of 454 rhesus miRNA genes, 173 share 100% homology with the human pre-miRNA (Category A), 281 have >90% and less than 100% homology with human precursor miRNA sequences (Category B, Table 1). While the majority of Category B putative rhesus miRNA sequences contained three or fewer mismatches compared with the mature human sequences, rhesus miRNA sequences (mm1-miR-) 220b, 518e, 557, 589, 625, 639, 650d had four mismatches. The detailed miRNA precursor sequences (shown as cDNA sequences for convenient analysis) and predicted hairpin structure for all 454 rhesus miRNA genes are listed in additional file 1. The 533 human miRNA gene sequences used to search the rhesus genome are available from the **Wellcome Trust Sanger Institute miRBase **[23].

**Table 1:** MiRNA Genes in Rhesus Genome

**Species**	**Known**	**Novel**^a^	**Total**	**A**^b^	**B**^b^
**Rhesus**	71^c^	383	454	173/454	281/454

**a**: Identified in current studies.

**b: Category A**: 100% homology with human precursors miRNA. **Category B**: greater than 90% and less than 100% homology between rhesus and human precursor sequences.

**c**: Previous published rhesus miRNA genes [33]

### MiRNA gene clusters in rhesus genome

**Figure 1:**
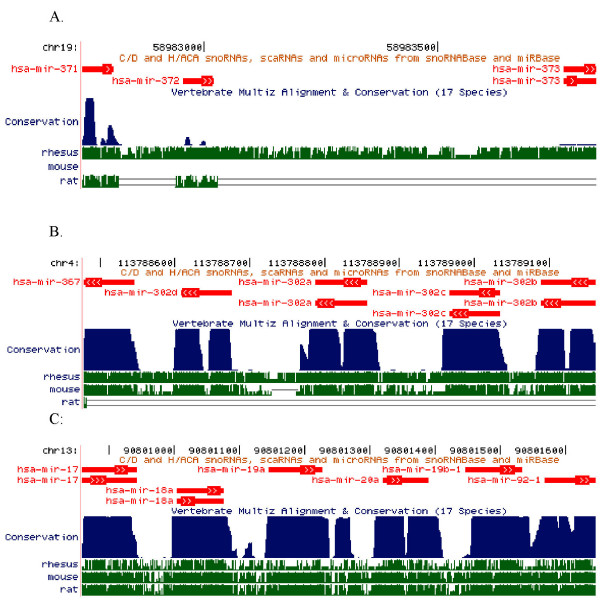
**MiRNA clusters in the human genome aligned with homologous regions of the rhesus, mouse and rat genomes**. (A) Human cluster miR-371/373 is located on chr19: 58,982,741–58,983,839 (1099 bp, [27]), Rhesus has the similar cluster also located on chr19 in rhesus. This cluster is not well conserved in mice and rats. (B) Human cluster miR-367/302b is on chr4: 113,926,634–113,927,317 [27] and hosted in antisense orientation in a 684 bp region, in rhesus and mouse. There is no similar cluster in rat genome. (C) Human miR-17/92 cluster is located on chr13: 90,800,860–90,801,646(787 bp, [27]) and is highly conserved among in the rhesus, mouse, and rat genomes.

MiRNA genes tend to be present as clusters in the genome [34,35]. Clusters were previously defined by Weber [36] as miRNA genes present in the same orientation and not separated by a transcriptional unit. Altuvia and colleagues [37] demonstrated that 42% of known human miRNA genes are arranged in clusters in the genome using a 3 kb threshold between two miRNA genes or 48% if the threshold is 10 kb. We found that at least some rhesus miRNAs are also arranged in clusters in the rhesus genome. Here we listed three human miRNA clusters (Figure 1) that have been associated with specific functions in previous studies. These miRNA clusters are located in regions of the genome that display substantial evolutionary conservation among 17 vertebrate species listed in the **UCSC Genome Bioinformatics **database: [27-29,38]. Sequence conservation in these regions of the human genome with rhesus, mouse and rat are indicated (Figure 1). Cluster miR-371/373 is located on chromosome 19 in human and rhesus, is expressed in human ES cells [18] and functions as an oncogene in human testicular carcinomas [21]. Rhesus miR-372 and miR-373 have 100% similarity in mature miRNA sequences with the human orthologs. Rhesus miR-371 has one nucleotide mismatch with the human sequence and rhesus miR-373* has three nucleotide differences (Table 2, Figure 1A). The mature sequences for miR-373 and miR-373* are encoded from the same pre-miRNA on complementary strands of the hairpin stem (see additional file 1).

**Table 2:** Comparison of MiRNA Clusters in Human and Rhesus

**Clusters**	**MiRNA**	**Chr**	**Mature MiRNA Sequences**^a^
MiR-371/373	hsa-miR-371	19	GUGCCGCCAU**C**UUUUGAGUGU
	mml-miR-371	19	GUGCCGCCAU**G**UUUUGAGUGU
	hsa-miR-372	19	AAAGUGCUGCGACAUUUGAGCGU
	mml-miR-372	19	AAAGUGCUGCGACAUUUGAGCGU
	hsa-miR-373*	19	AC**U**CAAAAUGGG**G**GC**G**CUUUCC
	mml-miR-373*	19	AC**C**CAAAAUGGG**A**GC**A**CUUUCC
	hsa-miR-373	19	GAAGUGCUUCGAUUUUGGGGUGU
	mml-miR-373	19	GAAGUGCUUCGAUUUUGGGGUGU
			
MiR-367/302b	hsa-miR-367	4	AAUUGCACUUUAGCAAUGGUGA
	mml-miR-367	5	AAUUGCACUUUAGCAAUGGUGA
	hsa-miR-302d	4	UAAGUGCUUCCAUGUUUGAGUGU
	mml-miR-302d	5	UAAGUGCUUCCAUGUUUGAGUGU
	hsa-miR-302a*	4	UAAACGUGGAUGUACUUGCUUU
	mml-miR-302a*	5	UAAACGUGGAUGUACUUGCUUU
	hsa-miR-302a	4	UAAGUGCUUCCAUGUUUUGGUGA
	mml-miR-302a	5	UAAGUGCUUCCAUGUUUUGGUGA
	hsa-miR-302c*	4	UUUAACAUGGGGGUACCUGCUG
	mml-miR-302c*	5	UUUAACAUGGGGGUACCUGCUG
	hsa-miR-302c	4	UAAGUGCUUCCAUGUUUCAGUGG
	mml-miR-302c	5	UAAGUGCUUCCAUGUUUCAGUGG
	hsa-miR-302b*	4	ACUUUAACAUGGAAGUGCUUUCU
	mml-miR-302b*	5	ACUUUAACAUGGAAGUGCUUUCU
	hsa-miR-302b	4	UAAGUGCUUCCAUGUUUUAGUAG
	mml-miR-302b	5	UAAGUGCUUCCAUGUUUUAGUAG
			
MiR-17/92	hsa-miR-17-5p	13	CAAAGUGCUUACAGUGCAGGUAGU
	mml-miR-17-5P	X	CAAAGUGCUUACAGUGCAGGUAGU
	hsa-miR-17-3p	13	ACUGCAGUGAAGGCACUUGU
	mml-miR-17-3p	X	ACUGCAGUGAAGGCACUUGU
	hsa-miR-18a	13	UAAGGUGCAUCUAGUGCAGAUA
	mml-miR-18a	X	UAAGGUGCAUCUAGUGCAGAUA
	hsa-miR-19a	13	UGUGCAAAUCUAUGCAAAACUGA
	mml-miR-19a	X	UGUGCAAAUCUAUGCAAAACUGA
	hsa-miR-20a	13	UAAAGUGCUUAUAGUGCAGGUAG
	mml-miR-20a	X	UAAAGUGCUUAUAGUGCAGGUAG
	hsa-miR-19b-1	13	UGUGCAAAUCCAUGCAAAACUGA
	mml-miR-19b-1	X	UGUGCAAAUCCAUGCAAAACUGA
	hsa-miR-92-1	13	UAUUGCACUUGUCCCGGCCUG
	mml-miR-92-1	X	UAUUGCACUUGUCCCGGCCUG

**a**: Mismatches between rhesus and human sequences are indicated with bold letters.

Cluster miR-367/302b genes are expressed in human and mouse ES cells and down-regulated during development into embryoid bodies [11,18]. We found this cluster shares 100% homology in mature miRNA sequence between rhesus (located on chromosome 5) and human (located on chromosome 4). This cluster is also well conserved in mouse, but not in rat (Figure 1B, Table 2). Clusters miR-17/92 (Figure 1C, Table 2) is highly conserved among human, rhesus, mouse and rat and has been reported to function as oncogenes and promote tumorigenesis [19]. This cluster is hosted on chr13 in human and chrX in rhesus.

### Rhesus miRNA gene families

Several Rhesus miRNA genes were considered family members based on sequence similarity criteria, as previously described [39]. The implications for miRNA gene amplification are still unknown, but miRNA genes with multiple copies may augment or amplify the physiological functions of individual miRNA genes. Among 454 rhesus miRNA genes, 32 exist in families with two or more copies. Here we listed five miRNA gene families that are present as multiple copies in the genome (Table 3). MiR-let-7a, miR-7, miR-9, miR-513 and miR-220 families contain three or more copies distributed on the same or different chromosomes that produce identical or slightly differed mature miRNAs.

**Table 3:** Rhesus MiRNA Gene Families

**MiRNA**	**Mature MiRNA**^a^	**Chr**	**Strand**	**Positions in Chr**^b^
mml-miR-let-7a-1	UGAGGUAGUAGGUUGUAUAGUU	15	+	105917273 – 105917352
mml-miR-let-7a-2		14	-	120554305 – 120554376
mml-miR-let-7a-3		10	+	90121100 – 90121173
				
mml-miR-7-1	UGGAAGACUAGUGAUUUUGUUG	15	-	92701750 – 92701859
mml-miR-7-2		7	+	68219893 – 68220002
mml-miR-7-3		19	+	4659068 – 4659177
				
mml-miR-9-1	UCUUUGGUUAUCUAGCUGUAUGA	1	-	135024723–135024811
mml-miR-9-2		6	-	84955411–84955497
mml-miR-9-3		7	+	68995340 – 68995429
				
mml-miR-513-1	UUCACAGGGAGGUGUCAUUUAU	X	-	145346049–145346177
mml-miR-513-2		X	-	145354602–145354730
mml-miR-513-3		X	-	145337083–145337214
				
mml-miR-202a	CCACCACCAUGUCUGACACUUU	X	-	121787204–121787314
mml-miR-202b	CCACCACC**G**UGUC**C**GACAC**C**UU	18	+	2383348 – 2383458
mml-miR-202c	CCACCAC**UG**UGUCUGACAC**C**UU	20	-	88058741–88058838
mml-miR-202d	CCACCACC**G**UGUCUGACAC**C**UU	4	+	3002388–3002492

**a**: Individual genes within a miRNA gene family have the same or different mature miRNA sequences (bold letters indicate the different nucleotides comparing with the mml-miR-202a).

**b**: position of rhesus miRNA genes in the genome can be found at [27].

### Detection of mature miRNA from rhesus tissues by performing polyA tailing RT-PCR

**Figure 2:**
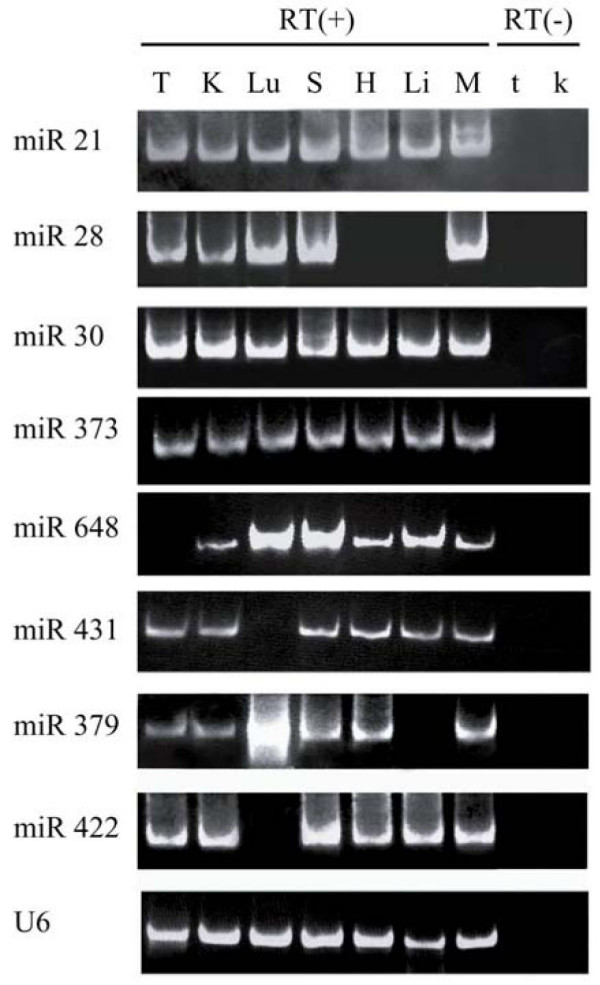
**Selected mature miRNA expression profiles in rhesus tissues**. PolyA tailing RT-PCR was used to detect miRNA expression in adult rhesus different tissues. T: testis, K: kidney, Lu: lung, S: spleen, H: heart, Li: liver, M: Skeletal muscle, t: RT(-) control from testis, k: RT(-) control from kidney. U6 snRNA was amplified as an internal control.

To test if the predicted miRNA genes generate mature miRNAs in adult rhesus tissues, 8 rhesus miRNAs were randomly selected from categories A and B (see Table 1 for category descriptions) and detected using the sensitive polyA tailing RT-PCR [40]. While miR-21, miR-30a and miR-373 were expressed in all 7 tissues including testis, kidney, lung, spleen, heart, liver and skeletal muscle, miR-422, miR-28, miR-379, miR-431 and miR-648 demonstrated more restricted expression patterns (Figure 2).

## Discussion

Based on homology searching of the rhesus genome by querying with human miRNA precursor sequences, we identified 454 miRNA genes, including 383 novel rhesus miRNAs, some of which are arranged in clusters as previously described for human. All of these miRNA genes and clusters are highly conserved between rhesus and human. RT-PCR analyses confirmed expression of rhesus miRNA genes in several tissues. Tissue specific regulation may indicate specialized roles in cell function and or tissue development. Stringent criteria were employed to identify the 454 rhesus miRNAs in this study and some additional miRNAs may yet be identified. While the rhesus genome was recently sequenced, it has not been fully assembled. In addition, rhesus miRNA genes were identified based on human orthologs, which may fail to identify some rhesus specific miRNAs and putative miRNA genes identified in this study contained at least a 16 nt pairing in the stem arm of the hairpin structure of the mature miRNA [31]. We frequently encounter 13 to 15 nt pairings in this core region from the predicted hairpin structure. We didn't consider them as novel miRNA genes in this study, but they could be potential miRNA genes. For example, hsa-miR-484 and bta-miR-484 have 15 nt and 12 nt pairings in stem arm of hairpin structure, respectively. Both hsa-miR-484 and bta-miR-484 have been verified experimentally as miRNA genes in human and bovine, respectively [22].

While we were writing this manuscript, Zhang et al. reported rhesus miR-506, 507, 508, 509-1, 509-2, 510 and 514 sequences [41]. These sequences are also predicted in the current study, although there were some differences in nomenclature. We employed the systematic annotation previously described for miRNAs [31].

## Conclusion

In the current study we identified 454 rhesus miRNA genes, including 71 that were previously reported. Identification of miRNA genes from rhesus will eventually provide the resources for analysis of expression profiles via microarray. These tools will help identify candidate miRNA genes associated with specific tissues, cells or biological functions.

## Methods

### Bioinformatics analysis

Human Pre-miRNA sequences were downloaded from **Wellcome Trust Sanger Institute miRBase, release 10.0 **[22-24] and used to search the rhesus genome at **UCSC Genome Bioinformatics **[27-29,38] for homologous sequences. Chromosomal location of putative rhesus miRNAs was determined at **UCSC Genome Bioinformatics **[27-29,38]. **ClustalW **[28,42,43] was used for sequences alignment. Precursor sequences were analyzed for secondary structure using **MFOLD **[44,45].

### PolyA tailing RT-PCR

Total RNA was extracted from the heart, liver, lung, kidney, skeletal muscle, spleen and testes of rhesus macaques with Trizol Reagent (Invitrogen, Carlsbad, CA). For polyA tailing RT-PCR, 5 μg total RNA from each tissue was treated with DNase(Invitrogen, Carlsbad, CA) and then polyadenylated using polyA polymerase (PAP, Ambion, Austin, TX) according to the manufacturer's instructions. The final reaction mixtures were extracted with phenol/chloroform, precipitated with ethanol and redisolved into 20 μl diethylpyrocarbonate(DEPC)-treated water. Half of the polyA tailed RNA was reverse transcribed into first-strand cDNA using Superscript III transcriptase (Invitrogen, Carlsbad, CA) with the oligodT adapter primer: 5'-GCGAGCACAGAATTAATACGACTCACTATAGGTTTTTTTTTTTTVN-3'. The remaining RNA was used for RT control without reverse transcriptase. For PCR, 2 ul of RT products were used as templates in each reaction. The reverse primer for each miRNA was from the same tailing sequences: 5'-TTCACGAATTTGCGTGTCAT-3'. The forward primers were specific to miRNA mature sequences, and U6 snRNA sequences were listed in Table 4. The PCR conditions were initially denatured at 94°C for 3 min followed by 30 cycles of 94°C for 30 sec, 60°C for 45 sec and 72°C for 30 sec, final extension for 5 min at 72°C. PCR products were separated on 12% native polyacrylamide gels and stained with ethidium bromide. U6 snRNA was amplified as an internal control.

**Table 4:** Primer Sequences for PolyA Tailing RT-PCR

**MiRNA**	**Primer Sequences**
mml-miR-21	TAGCTTATCAGACTGATGTTGA
mml-miR-30a	TGTAAACATCCTCGACTGGAAG
mml-miR-28	AAGGAGCTCACAGTCTATTGAG
mml-miR-373	ACCCAAAATGGGAGCACTTTCC
mml-miR-379	TGGTAGACTATGGAACGTA
mml-miR-422a	CTGGACTCAGGGTCAGAAGGCC
mml-miR-431	TGTCTTGCAGGCCGTCATGCA
mml-miR-648	AAGTGTGCAGGGCACTGAT
U6 Forward	GCTTGCTTCGGCAGCACATATAC
U6 Reverse	TGCATGTCATCCTTGCTCAGGG

## Authors' contributions

JY performed the genomic search and wrote the initial draft of manuscript. YS did RT-PCR and edited the draft. KEO provided experimental resources and advice and edited the final manuscript. All authors read and approved the final manuscript.

## Supplementary Material

Additional file 1 Pre-miRNA sequences in the rhesus genome and predicted secondary structures. The figure provides the pre-miRNA sequence and predicted secondary structure of 454 putative rhesus miRNA genes identified in the current study based on homology with human miRNA sequences. Click here for file
