# Supplementary material for: Identification of novel homologous microRNA genes in the rhesus macaque genome
Source: BMC Genomics. 2008 Jan 10;9:8. doi: 10.1186/1471-2164-9-8 (PMC2254598; doi:10.1186/1471-2164-9-8)
Supplement: Additional file 1 — Pre-miRNA sequences in the rhesus genome and predicted secondary structures. The figure provides the pre-miRNA sequence and predicted secondary structure of 454 putative rhesus miRNA genes identified in the current study based on homology with human miRNA sequences. [file 1471-2164-9-8-S1.pdf]

Supplementary Figure 1. Pre-miRNA sequences in the rhesus genome and predicated secondary structures

MiRNA name in black color: 100% homology with human precursor sequences (Category A).

MiRNA name in blue color: >90% homology with human precursor (Category B).

Lower case letter indicates mismatch with human miRNA sequences.

(Ψ) indicates the 71 rhesus miRNAs previously registered in miRNA database

(Red color indicates mature miRNA sequences)

In category A, the same names were used with the human.

In category B, the previous miRNA was indicated with number 1(e.g. miRNA-1), and all new miRNAs were numbered consecutively (e.g., miRNA-2, miRNA-3…).

mml-miR-1-1

GCTTGGGAAACATACTTCTTTATATGCCCATATGGACCTGCTAAGCTATGGAATGTAAAG
AAGTATGTATCTCAGGC

```
        10        20        30
        A                GC     ---|  AC
GCUUGGGA ACAUACUUCUUUAUAU  CCAUA   UGG  \
CGGACUCU UGUAUGAAGAAAUGUA  GGUAU   AUC  C
        A                A-     CGA^  GU
      70        60         50        40
```

mml-miR-1-2

ACCTACTCAGAGTACATACTTCTTTATGTACCCATATGAACATACAATGCTATGGAATGT
AAAGAAGTATGTATTTTTGGTAGGC

```
        10        20        30        40
A|     C                     AC     UGAACA
 CCUACU AGAGUACAUACUUCUUUAUGU  CCAUA      U
 GGAUGG UUUUAUGUAUGAAGAAAUGUA  GGUAU      A
C^     U                     A-     CGUAAC
    80        70        60         50
```

mml-miR-let-7a-1

TGGGATGAGGTAGTAGGTTGTATAGTTTTAGGGTCACACCCACCACTGGGAGA
TAACTATACAATCTACTGTCTTTCCTA

```
        10        20        30        40
     U   GU                 UUAGG|  ACA    C
UGGGA GAG  AGUAGGUUGUAUAGUU     GUC    CCCA C
AUCCU UUC  UCAUCUAACAUAUCAA     UAG    GGGU A
     -   UG                 -----^  A--    C
          70        60               50
```

mml-miR-let-7a-2

AGGcTGAGGTAGTAGGTTGTATAGTTTAGAATTACATCAAGGGAGATA
ACTGTACAGCCTCCTAGCTTTCCT

```
        10        20        30        40
|  cU   G   U             UAGAAUUAC   AA
AGG  GAG UAG AGGUUGUAUAGUU         AUC  G
UCC  UUC AUC UCCGACAUGUCAA         UAG  G
^  U-   G   C             ---------   AG
 70        60        50
```

mml-miR-let-7a-3

GGGTGAGGTAGTAGGTTGTATAGTTTGGGGCTCTGCCCTGCTATGGGATA
ACTATACAATCTACTGTCTTTCCT

```
        10        20                 30
   U   GU                ---------|     U
GGG GAG  AGUAGGUUGUAUAGUU         UGGGGC \
UCC UUC  UCAUCUAACAUAUCAA         GUCCCG C
   U   UG                UAGGGUAUC^     U
   70        60        50        40
```

mml-miR-let-7b

CGGGGTGAGGTAGTAGGTTGTGTGGTTTCAGGGCAGTGATGTTGCCCCTCAGAAGATAA
CTATACAACCTACTGCCTTCCCTG

```
        10        20            30
     U                    ----   A---|     UG
CGGGG GAGGUAGUAGGUUGUGUGGU    UUC    GGGCAG  \
GUCCC UUCCGUCAUCCAACAUAUCA    AAG    CCCGUU  A
     -                    AUAG   ACUC^     GU
  80         70        60        50        40
```

mml-miR-let-7c

GCATCCGGGTTGAGGTAGTAGGTTGTATGGTTTAGAGTTACACCCTGGGA
GTTAACTGTACAACCTTCTAGCTTTCCTTGGAGC

```
        10        20        30        40
| A      UU   G   U            UA  G UA AC
GC UCCGGG  GAG UAG AGGUUGUAUGGUU  GA U  C  \
CG AGGUUC  UUC AUC UCCAACAUGUCAA  UU A  G  C
^ -      CU   G   U            --  G GG UC
    80        70        60          50
```

mml-miR-let-7d

CCTAGGAAGAGGTAGTAGGTTGCATAGTTTTAGGGCAGGGATTTTGCCCACA
AGGAGGTAACTATACGACCTGCTGCCTTTCTTAGG

```
        10        20        30               40
      A               C      UUA-------|     GG
CCUAGG AGAGGUAGUAGGUUG AUAGUU          GGGCAG  \
GGAUUC UUUCCGUCGUCCAGC UAUCAA          CCCGUU  A
      -               A      UGGAGGAACA^     UU
       80        70        60        50
```

mml-miR-let-7e

```
CCCGGGCTGAGGTAGGAGGTTGTATAGTTGAGGAGGACACCCAAGGAGAT
CACTATACGGCCTCCTAGCTTTCCCCAGG
        10        20        30
  C   CU   G               U   GGA----| A
CC GGG  GAG UAGGAGGUUGUAUAG UGA       GG C
GG CCC  UUC AUCCUCCGGCAUAUC ACU       CC A
  A   CU   G               -   AGAGGAA^ C
        70        60         50        40
```

mml-miR-let-7f-1

```
TCAGAGTGAGGTAGTAGATTGTATAGTTGTGGGGTAGTGATTTTACCCTGTTCAGGAGA
TAACTATACAATCTATTGCCTTCCCTGA
        10        20        30
    AGU                       ---------|      UG
UCAG   GAGGUAGUAGAUUGUAUAGUUGU         GGGGUAG  \
AGUC   UUCCGUUAUCUAACAUAUCAAUA         UCCCAUU  A
    CC-                       GAGGACUUG^      UU
       80        70        60        50
```

mml-miR-let-7f-2

```
TGTGGGATGAGGTAGTAGATTGTATAGTTTTAGGGTCATACCCCATCTTG
GAGATAACTATACAGTCTACTGTCTTTCCCACG
        10        20            30        40
U      U   GU                ----|      UCAU
 GUGGGA GAG  AGUAGAUUGUAUAGU    UUUAGGG     A
 CACCCU UUC  UCAUCUGACAUAUCA    AGGUUCU     C
G      -   UG                AUAG^      ACCC
  80          70        60        50
```

mml-miR-let-7g

```
AGGCTGAGGTAGTAGTTTGTACAGTTTGAGGGTCTATGATACCACCCGGTACAGGAGA
TAACTGTACAGGCCACTGCCTTGCCA
        10        20        30         40
A|  U        A            UGAGG    A-   A    A
 GGC GAGGUAGU GUUUGUACAGUU     GUCU   UG UACC C
 CCG UUCCGUCA CGGACAUGUCAA     UAGA   AC AUGG C
A^  -        C            -----    GG   -    C
    80        70        60               50
```

mml-miR-let-7I

```
CTGGCTGAGGTAGTAGTTTGTGCTGTTGGTCGGGTTGTGACATTGCCCGCTGTGGAGATA
ACTGCGCAAGCTACTGCCTTGCTAG
        10        20               30
     U                 U   --------| U     UGUG
CUGGC GAGGUAGUAGUUUGUGC GUU        GG CGGGU    \
GAUCG UUCCGUCAUCGAACGCG CAA        UC GCCCG    A
     -                 U   UAGAGGUG^ -     UUAC
     80        70        60        50
```

mml-miR-7-1

```
TTGGATGTTGGCCTAGTTCTGTGTGGAAGACTAGTGATTTTGTTGTTTTT
AGATAACTAAATtGACAACAAATCACAGTCTGCCATATGGCACAGGCCAT
GCCTCTACAG
           10        20        30        40           50
U--    U  -|     A  U        A     A       U      --     A
   UGGA GU UGGCCU GU CUGUGUGG AGACU GUGAUUU GUUGUU  UUUAG U
   AUCU CG ACCGGA CA GGUAUACC UCUGA CACUAAA CAACAG  AAAUC A
GAC    C  U^     -  C        G     -       -      uU     A
```

```
.       100         90        80         70         60
```

mml-miR-7-2

CTGGATACAGAGTGaAgtGGCTGGCCCCgTCTGGAAGACTAGTGATTTTG
TTGTTGTCTTACTGCGCTCAACAACAAATCCCAGTCTgCCgAATGGTGCC
AGCCATtGCA

```
          20        30        40        50        60
  AG  A          C    C   A     AGU     U       UCUUA
    UG AGUGGCUGGC CCGU UGG AGACU   GAUUU GUUGUUG     C
    AC UUACCGACCG GGUA GCC UCUGA   CUAAA CAACAAC     U
  --  G          U    A   G     CC-     -       UCGCG
  110       100        90         80         70
```

mml-miR-7-3

AGATTAGAGTGGCTaTGGTCTAGTGCTGTGTGGAAGACTAGTGATTTTGT
TGTTCTGATGTgCTACGACAACAAaTCACAGCCGGCCTCATAGCGCAGAC
TCCCTTCGAC

```
        10        20        30        40        50        60
AGAUUA|  U  CUAU     A        U  AAGA  A       U      C  AU
      GAG GG    GGUCU GUGCUGUG GG    CU GUGAUUU GUUGUU UG  G
      CUU CC    UCAGA CGCGAUAC CC    GA CACUAAA CAACAG AU  U
CAG---^  C  ----     -        U  GGCC  -       -      C  CG
.              100         90        80          70
```

mml-miR-9-1

CGGGGTTGGTTGTTATCTTTGGTTATCTAGCTGTATGAGTGGTGTGGAGTCTTCATAAAG
CTAGATAACCGAAAGTAAAAATAACCCCA

```
        10        20        30        40
C|      GUUG   UC               G      U  UGU
 GGGGUUG    UUA  UUUGGUUAUCUAGCU UAUGAG GG   \
 CCCCAAU    AAU  AAGCCAAUAGAUCGA AUACUU CU   G
A^      AAA-   GA               A      -  GAG
        80         70        60          50
```

mml-miR-9-2

GGAAGCGAGTTGTTATCTTTGGTTATCTAGCTGTATGAGTGTATTGGTCTTCATAAAGCT
AGATAACCGAAAGTAAAAACTCCTTCA

```
        10        20        30        40
G|   C     G   UC               G      UG A
 GAAG GAGUU UUA  UUUGGUUAUCUAGCU UAUGAG  U U
 CUUC CUCAA AAU  AAGCCAAUAGAUCGA AUACUU  G U
A^   -     A   GA               A      CU G
       80        70        60        50
```

mml-miR-9-3

GGAGGCCCGTTTCTCTCTTTGGTTATCTAGCTGTATGAGTGCCACAGAGCCGctcTcAAGC
TAGATAACCGAAAGTAGAAATGAcTCTCA

```
        10         20        30         40
G    CC       -| C               GUAU    - CA
 GAGG  CGUUUCU CU UUUGGUUAUCUAGCU    GAGUG C  C
 CUCU  GUAAAGA GA AAGCCAAUAGAUCGA    cUcGC G  A
A    cA       U^ -               AcU-     C AG
.        80         70        60         50
```

mml-miR-10a

GATCTGTCTGTCTTCTGTATATACCCTGTAGATCCGAATTTGTGTAAGGAATTTTGTGGT
CACAAATTCGTATCTAGGGGAATATGTAGTTGACATAAACACTCCGCTC

```
              10        20        30        40        50
```

```
GAUC----   C--|   UU        A    G     C          UAAGGAA
        UGU   UGUC  CUGUAUAU CCCU UAGAU CGAAUUUGUG       U
        ACA   ACAG  GAUGUAUA GGGG AUCUA GCUUAAACAC       U
CUCGCCUC   AAU^   UU        A    -     U          UGGUGUU
       100        90        80         70        60
```

mml-miR-10b

CCAGAGGTTGTAACGTTGTCTATATATACCCTGTAGAACCGAATTTGTGT
GGTATCCaTATAGTCACAGATTCGATTCTAGGGGAATATATGGTCGATGC
AAAAACTTCA

```
        10        20        30        40        50
CCA|     GUAA     U        A    G     C          UG-   U
   GAGGUU    CGUUG CUAUAUAU CCCU UAGAA CGAAUUUGUG   GUA C
   CUUCAA    GUAGC GGUAUAUA GGGG AUCUU GCUUAGACAC   UAU C
A--^     AAAC     U        A    -     A          UGA   a
.         100        90         80        70        60
```

mml-miR-15a(Ψ)

CCTTGGAGTAAAGTAGCAGCACATAATGGTTTGTGGATTTTGAAAAGGTG
CAGGCCATATTGTGCTGCCTCAAAAATACAAGG

```
        10        20        30        40
|    GAGUAAAGUA        UA          GA    U
CCUUG          GCAGCACA  AUGGUUUGUG  UUU \
GGAAC          CGUCGUGU  UACCGGACGU  AAA G
^    AUAAAAACUC        UA          GG    A
  80        70        60        50
```

mml-miR-15b(ψ)

TTGAGGCCTTAAAGTACTGTAGCAGCACATCATGGTTTACATaCTACAGTCAAGATGCGA
ATCATTATTTGCTGCTCTAGAAATTTAAGGAAATTCAT

```
          10        20        30        40
U   GG--|      GUA   U       C  C      UA    a  ACA
 UGA    CCUUAAA   CUG AGCAGCA AU AUGGUU  CAU CU   \
 ACU    GGAAUUU   GAU UCGUCGU UA UACUAA  GUA GA   G
U   UAAA^      AAA   C       U  U      GC    -  ACU
       90        80        70        60         50
```

mml-miR-16-1(ψ)

GTCAGCAGTGCCTTAGCAGCACGTAAATATTGGCGTTAAGATTCTAAAAT
TATCTCCAGTATTAACTGTGCTGCTGAAGTAAGGTTGAC

```
        10        20         30        40
      AG   C          -| A        CGUUA    UCUA
GUCAGC  UGC UUAGCAGCAC GU AAUAUUGG     AGAU    \
CAGUUG  AUG AGUCGUCGUG CA UUAUGACC     UCUA    A
      GA   A          U^ A        -----    UUAA
        80        70        60             50
```

mml-miR-16-2

GTTCCACTCTAGCAGCACGTAAATATTGGCGTAGTGAAATATgTATTAAA
CACCAATATTACTGTGCTGCTTcAGTGTGAC

```
        10         20        30        40
  UC    CU         -|   A      C  AG   AAU
GU  CACU  AGCAGCAC GUAA UAUUGG GU  UGA   A
CA  GUGA  UCGUCGUG CAUU AUAACC CA  AUU   U
  GU    cU         U^   -      A  A-   AUg
80        70        60         50
```

mml-miR-17-5p(ψ)

GTCAGAATAATGTCAAAGTGCTTACAGTGCAGGTAGTGATATGTGCATCTACTGCAGTGA
AGGCACTTGTAGCATTATGGTGAC

```
        10         20        30         40
    GA       CA-|       A  G     G   -  AUA
GUCA  AUAAUGU   AAGUGCUU CA UGCAG UAG UG   \
CAGU  UAUUACG   UUCACGGA GU ACGUC AUC AC   U
    GG       AUG^       A  G     -   U  GUG
   80        70        60         50
```

mml-miR-17-3p

```
        10         20        30         40
    GA       CA-|       A  G     G   -  AUA
GUCA  AUAAUGU   AAGUGCUU CA UGCAG UAG UG   \
CAGU  UAUUACG   UUCACGGA GU ACGUC AUC AC   U
    GG       AUG^       A  G     -   U  GUG
   80        70        60         50
```

mml-miR-18a(ψ)

TGTTCTAAGGTGCATCTAGTGCAGATAGTGAAGTAGATTAGCATCTACTGCC
CTAAGTGCTCCTTCTGGCA

```
           10          20        30        40
GU---         U   --|  U   UC   U    A   UGAA  AG
     GCUUUUUGU CUA  AGG GCA  UAG GCAG UAG    GU  A
     UGAAGAAUA GGU  UCC CGU  AUC CGUC AUC    CG  U
UGUAU         C   CU^  U   GA   C    -   UA--  AU
 90        80        70        60           50
```

mml-miR-18b

TGTGTTAAGGTGCATCTAGTGCAGTTAGTGAAGCAGCTTAGAATCTACTGCCCTAAATGC
CCCTTCTGGCA

```
          10        20        30
UG     --|  U    C   U     UA  GAAGCA
  UGUUA  AGG GCAU UAG GCAGU  GU      \
  ACGGU  UCC CGUA AUC CGUCA  UA      G
--     CU^  C    A   C     UC  AGAUUC
  70        60        50        40
```

mml-miR-19a(ψ)

GCAGTCCTCTGTTAGTTTTGCATAGTTGCACTACAAGAAGAATGTAGTTGTGCAAATCTA
TGCAAAACTGATGGTGGCCTGC

```
        10        20             30
    U  U                 --    ---|     AGA
GCAG CC CUGUUAGUUUUGCAUAG  UUGC   ACUACA   \
CGUC GG GGUAGUCAAAACGUAUC  AACG   UGAUGU   A
    C  U                 UA    UGU^     AAG
 80        70        60        50        40
```

mml-miR-19b-1(ψ)

CACTGTTCTATGGTTAGTTTTGCAGGTTTGCATCCAGCTGTGTGATATTCTGCTGTGCAAATC
CATGCAAAACTGACTGTGGTAGTG

```
       10        20          30        40
|    UU                 -  -      UC    UGUGUG
CACUG  CUAUGGUUAGUUUUGCA GG UUUGCA  CAGC      \
GUGAU  GGUGUCAGUCAAAACGU CC AAACGU  GUCG      A
^    --                 A  U      --    UCUUAU
        80        70        60          50
```

mml-miR-19b-2(ψ)
ACATTGCTACTTACAATTAGTTTTGCAGGTTTGCATTTCAGCGTATATATGTATATGTG
GCTGTGCAAATCCATGCAAAACTGATTGTGATAATGT
```
        10        20          30        40
|     CUAC                 -  -       UUCA       U
ACAUUG    UUACAAUUAGUUUUGCA GG UUUGCAU    GCGUAUA A
UGUAAU    AGUGUUAGUCAAAACGU CC AAACGUG    UGUAUAU U
^     ----                 A  U       UCGG       G
         90        80        70        60        50
```

mml-miR-20a(ψ)
GTAGCACTAAAGTGCTTATAGTGCAGGTAGTGTTTAGTTATCTACTGCATTATGAGCACT
TAAAGTACTGC
```
         10        20        30
    C   A-|                G   -  UU
GUAG ACU  AAGUGCUUAUAGUGCAG UAG UG  U
CGUC UGA  UUCACGAGUAUUACGUC AUC AU  A
    A   AA^                -   U  UG
70        60        50         40
```

mml-miR-20b
AGTACCAAAGTGCTCATAGTGCAGGTAGTTTTGGCATGACTCTACTGTAGTGTGGGCACT
TCCAGTACT
```
         10        20         30
     CA-           G     GU-|   UUG
AGUAC   AAGUGCUCAUA UGCAG   AGUU   \
UCAUG   UUCACGGGUGU AUGUC   UCAG   G
     ACC           G     AUC^   UAC
        60        50        40
```

mml-miR-21(ψ)
TGTCGGGTAGCTTATCAGACTGATGTTGACTGTTGAATCTCATGGCAACACCAGTCGATG
GGCTGTCTGACA
```
        10        20        30
U|    GU        A     A     A    U AA
 GUCGG  AGCUUAUC GACUG UGUUG CUGU G  \
 CAGUC  UCGGGUAG CUGAC ACAAC GGUA C  U
A^    UG        -     C     -    - UC
 70        60         50          40
```

mml-miR-22(ψ)
GGCTGAGCCGCAGTAGTTCTTCAGTGGCAAGCTTTATGTCCTGACCCAGCTAAAGCTGCCA
GTTGAAGAACTGTTGCCCTCTGCC
```
        10        20         30        40
|  U   CC               -     A      U  CCUG
GGC GAG  GCAGUAGUUCUUCAG UGGCA GCUUUA GU    \
CCG CUC  CGUUGUCAAGAAGUU ACCGU CGAAAU CG    A
^  U   C-               G     -      -  ACCC
    80         70         60          50
```

mml-miR-23a(ψ)
GGCCGGCTGGGGTTCCTGGGGATGGGATTTGCTTCCTGTCACAAATCACATTGCCAGGGA
TTTCCAACCGACC
```
        10         20        30
  C   C   -|         G    G      CUUC
GG CGG UGG GGUUCCUGG GAUG GAUUUG    C
```

```
CC GCC ACC UUAGGGACC UUAC CUAAAC    U
  A   A   U^        G    A      ACUG
  70        60        50        40
```

mml-miR-23b

CTCAGGTGCTCTGGCTGCTTGGGTTCCTGGCATGCTGATTTGTGACTTAAGATTAAAATC
ACATTGCCAGGGATTACCACGCAACCACGACCTTGGC

```
         10        20          30         40
-  C    GCUC   C   U   --         -|  C      GUGACU
 CU AGGU    UGG UGC UGG  GUUCCUGGC AUG UGAUUU      U
 GG UCCA    ACC ACG ACC  UAGGGACCG UAC ACUAAA      A
C  U    GC--   A   C   AU        U^  -      AUUAGA
      90          80        70         60        50
```

mml-miR-24-1/189(ψ)

CTCCGGTGCCTACTGAGCTGATATCAGTTCTCATTTTACACACTGGCTCAGTTCAGCAGG
AACAGGAG

```
        10        20        30
|   G  G   A         UA      UCUCAU
CUCC GU CCU CUGAGCUGA  UCAGU      \
GAGG CA GGA GACUUGACU  GGUCA      U
^   A  A   C         C-      CACAUU
       60        50        40
```

mml-miR-24-2(ψ)

CTCTGCCTCCCGTGCCTACTGAGCTGAAACACAGTTGGTTTGTGcACACTGGCTCAGTTCA
GCAGGAACAGG

```
        10        20        30        40
CUCUGCCU| C  G   A         AACA    UG  U
        CC GU CCU CUGAGCUGA    CAGU  GU U
        GG CA GGA GACUUGACU    GUCA  cG G
--------^ A  A   C         CG--    CA  U
         70        60          50
```

mml-miR-25(ψ)

GGCCAGTGTTGAGAGGCGGAGACTTGGGCAATTGCTGGACGCTGCCCTGG
GCATTGCACTTGTCTCGGTCTGACAGTGCCGGCC

```
        10        20         30          40
    A UG    AG     G     UU   -    U    --| ACG
GGCC G  UUG  AGGC GAGAC  GG GCAA UGCU  GG    \
CCGG C  GAC  UCUG CUCUG  UC CGUU ACGG  CC    C
    C GU    AG     G     U-   A    -    GU^ CGU
   80        70         60         50
```

mml-miR-26a-1(ψ)

GTGGCCTCGTTCAAGTAATCCAGGATAGGCTGTGCAGGTCCCAATGGGCCTATTCTTGGT
TACTTGCACGGGGACGC

```
        10        20        30        40
|  G      U         C          GUG  GGU
GUG CCUCGU CAAGUAAUC AGGAUAGGCU   CA   C
CGC GGGGCA GUUCAUUGG UCUUAUCCGG   GU   C
^  A      C         U          ---  AAC
      70        60        50
```

mml-miR-26a-2

GGCTGTGGCTGGATTCAAGTAATCCAGGATAGGCTGTTTCCATCTGTGAG
GCCTATTCTTGATTACTTGTTTCTGGAGGCAGCT

```
        10        20        30        40
|     GG UG  UU         C          GUUUCC
GGCUGU  C  GA  CAAGUAAUC AGGAUAGGCU      A
UCGACG  G  CU  GUUCAUUAG UCUUAUCCGG      U
^     GA GU  UU         U          AGUGUC
   80        70        60        50
```

mml-miR-26b

CCGGGACCCAGTTCAAGTAATTCAGGATAGGTTGTGTGCTGTCCAGCCTGTTCTCCATTA
CTTGGCTCGGGGACCGG

```
         10        20        30
    GA   -|  U        UC           UGUG
CCGG  CCC AGU CAAGUAAU  AGGAUAGGUUG    \
GGCC  GGG UCG GUUCAUUA  UCUUGUCCGAC    C
    AG   C^  -        CC           CUGU
      70         60        50        40
```

mml-miR-27a(ψ)

CTGAGGAGCAGGGCTTAGCTGCTTGTGAGCAGGGTCCACACCAAGTCGTGTTCACAGTGG
CTAAGTTCCGCCCCCCAG

```
        10        20        30
|  A  A  A         UG  U       G  UCCAC
CUG GG GC GGGCUUAGC  CU GUGAGCA GG     \
GAC CC CG CUUGAAUCG  GA CACUUGU CU     A
^  C  C  C         GU  -       G  GAACC
       70        60         50
```

mml-miR-27b

ACCTCTCTAACAAGGTGCAGAGCTTAGCTGATTGGTGAACAGTGATTGGTTTCCGCTTTG
TTCACAGTGGCTAAGTTCTGCACCTGAAGAGAAGGTG

```
          10        20        30        40        50
-   -|    AACA                  AUUG        UGAU   U
 ACC UCUCU    AGGUGCAGAGCUUAGCUG    GUGAACAG    UGG \
 UGG AGAGA    UCCACGUCUUGAAUCGGU    CACUUGUU    GCC U
G   A^    AG--                  GA--        UC--   U
      90          80        70          60
```

mml-miR-28(ψ)

GGTCCTTGCCCTCAAGGAGCTCACAGTCTATTGAGTTtCCTTTgTGACTT
TCCCACTAGATTGTGAGCTCCTGGAGGGCAGGCACT

```
        10        20        30           40
   C         A                UU----|    tCC
GGU CUUGCCCUC AGGAGCUCACAGUCUA      GAGUU   U
UCA GGACGGGAG UCCUCGAGUGUUAGAU      UUCAG   U
   C         G                CACCCU^    UgU
     80        70        60        50
```

mml-miR-29a(ψ)

ATGACTGATTTCTTTTGGTGTTCAGAGTCAATATAATTTTCTAGCACCATCTGAAATCGGTTAT

```
        10        20        30
|           UUU       C    UCAAU
AUGACUGAUUUC   UGGUGUU AGAG     \
UAUUGGCUAAAG   ACCACGA UCUU     A
^           UCU       -    UUAAU
   60        50         40
```

mml-miR-29b-1

CTTCAGGAAGCTGGTTTCATATGGTGGTTTAGATTTAAATAGTGATTGTCTAGCACCATT
TGAAATCAGTGTTCTTGGGGG

```
          10        20        30        40
-         -|         U      GU      UUAAAU
 CUUCAGGAA GCUGGUUUCA AUGGUG  UUAGAU      \
 GGGGUUCUU UGACUAAAGU UACCAC  GAUCUG      A
G         G^         U      --      UUAGUG
80        70        60          50
```

<u>mml-miR-29b-2</u>

CTTCTGGAAGCTGGTTTCACATGGTGGCTTAGATTTTTCCATCTTTGTATCTAGCACCAT
TTGAAATCAGTGTTTTAGGAG

```
         10        20        30        40
         -|         C      G  U     UUUUCC
CUUCUGGAA GCUGGUUUCA AUGGUG CU AGAU      A
GAGGAUUUU UGACUAAAGU UACCAC GA UCUA      U
         G^         U      -  -     UGUUUC
80        70        60          50
```

<u>mml-miR-29c</u>

ATCTCTTACACAGGCTGACCGATTTCTCCTGGTGTTCAGAGTCTGTTTTTGTCTAGCACC
ATTTGAAATCGGTTATGATGTAGGGGGA

```
        10          20        30           40
A         -| GGC          UCC       ---     UC
 UCUCUUACA CA   UGACCGAUUUC   UGGUGUU   CAGAG  \
 GGGGGAUGU GU   AUUGGCUAAAG   ACCACGA   GUUUU  U
A         A^ ---          UUU       UCU     UG
       80           70        60        50
```

<u>mml-miR-30a-5p(ψ)</u>

GCGACTGTAAACATCCTCGACTGGAAGCTGTGAAGCCAtAGATGGGCTTTCAGTCGGATG
TTTGCAGCTGC

```
        10        20             30
   A           UC           -----|  A
GCG CUGUAAACAUCC  GACUGGAAGCU     GUG  A
CGU GACGUUUGUAGG  CUGACUUUCGG     uAC  G
   C           --           GUAGA^  C
70        60          50        40
```

<u>mml-miR-30a-3p</u>

```
        10        20             30
   A           UC           -----|  A
GCG CUGUAAACAUCC  GACUGGAAGCU     GUG  A
CGU GACGUUUGUAGG  CUGACUUUCGG     UAC  G
   C           --           GUAGA^  C
70        60          50        40
```

<u>mml-miR-30b</u>(ψ)

ACCAAGTTTCAGTTCATGTAAACATCCTACACTCAGCTGTAATACATGGATTGGCTGGGA
GGTGGATGTTTACTTCAGCTGACTTGGA

```
        10        20         30         40
A|    UU      CAU         U  A-         UAAUA
 CCAAG  UCAGUU   GUAAACAUCC AC  CUCAGCUG     C
 GGUUC  AGUCGA   CAUUUGUAGG UG  GGGUCGGU     A
A^    --      CUU         -  GA         UAGGU
         80        70         60        50
```

<u>mml-miR-30c-1</u>

```
ACCATGCTGTAGTGTGTGTAAACATCCTACACTCTCAGCTGTGAGCTCAAGGTGGCTGGG
AGAGGGTTGTTTACTCCTTCTGCCATGGA
        10        20        30        40
A|    CU    UGUGU       U   ACA        UG GAGC
 CCAUG  GUAG     GUAAACA CCU   CUCUCAGC  U    \
 GGUAC  CGUC     CAUUUGU GGG   GAGGGUCG  G    U
A^    --    UUCCU       U   A--        GU GAAC
          80        70          60        50
```

mml-miR-30c-2

```
AGATACTGTAAACATCCTACACTCTCAGCTGTGGAAAGTAAGAAAGCTGG
GAGAAGGCTGTTTACTCTcTCT
        10        20        30
|  UACU       U   ACA         GUGGAA
AGA    GUAAACA CCU   CUCUCAGCU      A
UCU    CAUUUGU GGA   GAGGGUCGA      G
^  CuCU       C   A--         AAGAAU
 70        60          50        40
```

mml-miR-30d

```
TTGTTGTAAACATCCCCGACTGGAAGCTGTAAGACACAGCTAAGCTTTCAGTC
AGATGTTTGCTGCTAC
         10        20        30
UU-| U         CCC         GUAAGA
   GU GUAAACAUC   GACUGGAAGCU      C
   CG CGUUUGUAG   CUGACUUUCGA      A
CAU^ U         A--         AUCGAC
        60          50        40
```

mml-miR-30e

```
GGGCAGTCTTcGCTACTGTAAACATCCTTGACTGGAAGCTGTAAGGTGTTCAGAGGAGCT
TTCAGTCGGATGTTTACAGCGGCAGGCTGCCA
        10        20        30        40
G|       Uc  UA           UU          GUAAGGU
 GGCAGUCU  GC  CUGUAAACAUCC  GACUGGAAGCU       G
 CCGUCGGA  CG  GACAUUUGUAGG  CUGACUUUCGA       U
A^       --  GC           --          GGAGACU
 90          80        70          60        50
```

mml-miR-30e*

```
        10        20        30        40
G|       Uc  UA           UU          GUAAGGU
 GGCAGUCU  GC  CUGUAAACAUCC  GACUGGAAGCU       G
 CCGUCGGA  CG  GACAUUUGUAGG  CUGACUUUCGA       U
A^       --  GC           --          GGAGACU
 90          80        70          60        50
```

mml-miR-31(ψ)

```
GGAGAGGAGGCAAGAUGCTGGCATAGCTGTTGAACTGGGAACCTGCTATG
CCAACATATTGCCATCTTTCC
        10        20         30
      GA     G   C         U-|  GAA
GGAGAG  GGCAA AUG UGGCAUAGC   GUU   C
CCUUUC  CCGUU UAC ACCGUAUCG   CAA   U
      UA     A   A         UC^  GGG
70        60        50        40
```

mml-miR-32(ψ)

GGAGATATTGCACATTACTAAGTTGCATGTTGTCACGGCCTCAATGCAAT
TTAGTGTGTGTGATATTTTC

```
        10        20          30
|       UG      U            - UU  C
GGAGAUAU  CACAU ACUAAGUUGCAU G  GU A
CUUUUAUA  GUGUG UGAUUUAACGUA C  CG C
^       GU      -            A UC  G
.        60         50        40
```

mml-miR-33a(ψ)
CTGCGGTGCATTGTAGTTGCATTGCATGTTCTGGTGGTACCCATGCAATGTTTCCACAGTGCATTACAG

```
        10        20          30
|  CG         AGUU          UUCU  UG
CUG  GUGCAUUGU    GCAUUGCAUG    GG  \
GAC  UACGUGACA    UGUAACGUAC    CC  G
^  AU         CCUU          ----  AU
        60        50             40
```

mml-miR-33b
GCGGGCGGCCCCGCGGTGCATTGCTGTTGCATTGCACGTGTGTGAGGCGGGTGCAGTGCC
TCGGCAGTGCAGCCCGGAGCCGGCCCCTGGCACCgC

```
                 10         20        30        40
-----  ---|   -   C   C  -          UU         G   GU
     GC   GGGC GGC CCG GG UGCAUUGCUG  GCAUUGCAC UGU  \
     CG   CCCG CCG GGC CC ACGUGACGGC  CGUGACGUG GCG  G
CgCCA  GUC^   G   A   -  G          UC         G   GA
      90        80         70        60        50
```

mml-miR-34a(ψ)
GGCCAGCTGTGAGTGTTTCTTTGGCAGTGTCTTAGCTGGTTGTTGTGAGC
AATAGTAAGGAAGCAATCAGCAAGTATACTGCCCTAGAAGTGCTaCACaT
TGTGGGGCC

```
           10         20        30         40         50
    A-  --    -  UG      UU        -|  A          GUGA-  A
GGCC  GC  UGUG AG  UUUCU  GGCAGUGU CUU GCUGGUUGUU     GC A
CCGG  UG  aCAC UC  GAAGA  CCGUCAUA GAA CGACUAACGA     UG U
    GG  UU    a  GU      UC        U^  -          AGGAA  A
       100        90        80         70         60
```

mml-miR-34b
GTGCTCGGTTTGTAGGCAGTGTCATTAGCTGATTGTACTGTGGTGGTTACAATCACTAAC
TCCACTGCCATCAAAACAAGGCAC

```
        10         20        30          40
     CG    GUA-|      UCA    C        -  GU
GUGCU  GUUU    GGCAGUG   UUAG UGAUUGUA CU  \
CACGG  CAAA    CCGUCAC   AAUC ACUAACAU GG  G
     AA    ACUA^      CUC    -        U  UG
   80        70        60         50
```

mml-miR-34c
AGTCTAGTTACcAGGCAGTGTAGTTAGCTGATTGCTGATAGTACCAATCACTAACCACAC
GGCCAGGTAAAAAGATT

```
        10        20        30
|    AG     A   A    A     C      C  A
AGUCU  UUACc GGC GUGU GUUAG UGAUUG UG U
UUAGA  AAUGG CCG CACA CAAUC ACUAAC AU A
^    AA     A   G    C     -      C  G
      70        60         50        40
```

mml-miR-92a-1(ψ)

CTTTCTACACAGGTTGGGATCGGTTGCAATGCTGTGTTTCTGTATGGTATTGCACTTGTCCC
GGCCTGTTGAGTTTGG

```
         10        20        30
CUUU-| AC            C   U           UU
     CU  ACAGGUUGGGAU GGU GCAAUGCUGUG  U
     GA  UGUCCGGCCCUG UCA CGUUAUGGUAU  C
GGUUU^ GU            U   -           GU
       70        60         50        40
```

mml-miR-92a-2

TCATCCCTGGGTGGGGATTTGTTGCATTACTTGTGTTCTATATAAAGTATTGCACTTGTC
CCGGCCTGTGGAAGA

```
        10        20        30
| A   C     G     UU  U   U     -    U
UC UCC UGGGU GGGAU  GU GCA UACUU GUGU \
AG AGG GUCCG CCCUG  CA CGU AUGAA UAUA C
^ A   U     G     UU  -   U     A    U
    70        60         50        40
```

mml-miR-92b

CGGGCCCCGGGCGGGCGGGAGGGACGGGACGCGGTGCAGTGTTGTTcTTTCCCCCGCCAA
TATTGCACTCGTCCCGGCCTCCGGCCCCCCCGGCCC

```
       10        20        30        40        50
C|    CC   C    G     GA       C            UUcUUU
 GGGCC  GGG GGGC GGAGG  CGGGACG GGUGCAGUGUUG      \
 CCCGG  CCC CCCG CCUCC  GCCCUGC UCACGUUAUAAC      C
-^    C-   -    G     G-       -            CGCCCC
       90         80         70         60
```

mml-miR-93(ψ)

CTGGGGGCTCCAAAGTGCTGTTCGTGCAGGTAGTGTGATTAaCCgACCTACTGCTGAGCT
AGCACTTCCCGAGCCCCCaG

```
        10          20        30        40
          CA-         -|    U    G    UG  AU
CUGGGGGCUC   AAGUGCU GUUCG GCAG UAG   UG  U
GaCCCCCGAG   UUCACGA CGAGU CGUC AUC   gC  A
          CCC        U^    -    -    CA  Ca
.        70         60           50
```

mml-miR-95

AACACAGTGGGCACTCAATAAATGTCTGTTGAATTGAAATGCGTTACATTCAACGGGTAT
TTATTGAGCACCCACTCTGTG

```
        10        20        30          40
A   C      CA          GU        -|  AA
 ACA AGUGGG  CUCAAUAAAU  CUGUUGAAU UGA  U
 UGU UCACCC  GAGUUAUUUA  GGCAACUUA AUU  G
G   C      AC          UG        C^  GC
80        70         60        50
```

mml-miR-96(ψ)

TGGCCGATTTTGGCACTAGCACATTTTTGCTTGTGTCTCTCCGCTCTGAG
CAATCATGTGCAGTGCCAATATGGGAAA

```
        10        20        30
UGG  G  U        A      UU      ---|  UC
   CC AU UUGGCACU GCACAU  UUGCUU   GUG  U
```

```
   GG UA AACCGUGA CGUGUA  AACGAG   CGC  C
AAA  G  U        -      CU      UCU^  CU
       70         60         50         40
```

mml-miR-98(ψ)

AGGATTgTGCTCATGCCAGGGTGAGGTAGTAAGTTGTATTGTTGTGGGGT
AGGGATATTAGGCCCCAATTAGAAGATAACTATACAACTTACTACTTTCC
CTGGTGTGTGGCATATTCA

```
        10         20        30        40                 50
AG  U      -          U                 U    ---------|     AG   A
  GA UgUGCU CAUGCCAGGG GAGGUAGUAAGUUGUAU GUUG         UGGGGU  GG \
  CU AUACGG GUGUGGUCCC UUUCAUCAUUCAACAUA CAAU         ACCCCG  UU U
A-  U      U          -                 U    AGAAGAUUA^     GA   A
        110       100         90        80        70        60
```

mml-miR-99a(ψ)

CCCATTGGCATAAACCCGTAGATCCGATCTTGTGGTGAAGTGGACCGCACAAGCTCGCTT
CTATGGGTCTGTGTCAGTGTG

```
        10         20       30        40
CC|         A         UC   U      G  AAG
  CAUUGGCAUA ACCCGUAGA  CGA CUUGUG UG   U
  GUGACUGUGU UGGGUAUCU  GCU GAACAC GC   G
GU^         C         UC   C      -  CAG
80        70        60        50
```

mml-miR-99b

GGCACCCACCCGTAGAACCGACCTTGCGGGGCCTTCGCCGCACACAAGCTCGTGTCTGTG
GGTCCGTGTC

```
        10        20             30
     CC         AC   C  ----     -| C
GGCAC  ACCCGUAGA  CGA CU    UGCGG GG \
CUGUG  UGGGUGUCU  GCU GA    ACGCC CU C
     CC         GU   C  ACAC     G^ U
.        60        50        40
```

mml-miR-100(ψ)

CCTGTTGCCACAAACCCGTAGATCCGAACTTGTGGTATTAGTCCGCACAAGCTTGTgTCT
ATAGGTATGTGTCTtTTAGG

```
         10        20        30
    UUGC-|   A   CG    UC   A      GUAUU
CCUG     CACA ACC  UAGA  CGA CUUGUG      \
GGAU     GUGU UGG  AUCU  GUU GAACAC      A
    UuUCU^   A   AU    gU   C      GCCUG
.        70        60        50
```

mml-miR-101-1(ψ)

TGCCCTGGCTCAGTTATCACAGTGCTGATGCTGTCcATTCTAAAGGTACA
GTACTGTGATAACTGAAGGATGGCA

```
        10        20         30
U|  CUGGC                  A    GUCcA
 GCC     UCAGUUAUCACAGUGCUG UGCU     U
 CGG     AGUCAAUAGUGUCAUGAC AUGG     U
A^  UAGGA                  -    AAAUC
    70        60        50          40
```

mml-miR-101-2

ACTGTCCTTTTTCGGTTATCATGGTACCGATGCTGTATATCTGAAGGTA

CAGTACTGTGATAACTGAAGAATGGTGGT

```
        10        20         30       40
| UG  C                  ACCGAU       AUCU
AC  UC UUUUUCGGUUAUCAUGGU      GCUGUAU    \
UG  GG AAGAAGUCAAUAGUGUCA      UGACAUG    G
^ GU  U                  ------       GAAA
        70        60               50
```

mml-miR-103-1(ψ)

TACTGCCCTCGGCTTCTTTACAGTGCTGCCTTGTTGCATATGGATCAAGC
AGCATTGTACAGGGCTATGAAGGCATTG

```
        10          20         30
UAC    C  --|   U  U           C   UUGCA
   UGCC UC  GGCU CU UACAGUGCUGC UUG     U
   ACGG AG  UCGG GA AUGUUACGACG AAC     A
GUU    A  UA^   -  C           -   UAGGU
       70         60        50         40
```

mml-miR-103-2

TTGTGCTTTCAGCTTCTTTACAGTGCTGCCTTGTAGCATTCAGGTCAAGC
AGCATTGTACAGGGCTATGAAAGAACCA

```
        10          20           30
UU  G     --   U   U           ----|   UAG
  GU CUUUC  AGC UCU UACAGUGCUGC    CUUG   \
  CA GAAAG  UCG GGA AUGUUACGACG    GGAC   C
AC  A     UA   -   C           AACU^   UUA
       70         60        50        40
```

mml-miR-105-1(ψ)

TGTGCATCGTGGTCAAATGCTCAGACTCCTGTGGTGGCTGCTCATGCACC
ACGGATGTTTGAGCATGTGCTACGGTGTCTA

```
        10        20        30        40
UGU|        U  A          UC        G UGC
   GCAUCGUGG CA AUGCUCAGAC  CUGUGGUG C   \
   UGUGGCAUC GU UACGAGUUUG  GGCACCAC G   U
AUC^        -  G          UA        - UAC
80        70         60        50
```

mml-miR-105-2

TGTGCATCGTGGTCAAATGCTCAGACTCCTGTGGTGGCTGCTTATGCACC
ACGGATGTTTGAGCATGTGCTATGGTGTCTA

```
        10        20        30        40
UGU|        U  A          UC        G UGC
   GCAUCGUGG CA AUGCUCAGAC  CUGUGGUG C   \
   UGUGGUAUC GU UACGAGUUUG  GGCACCAC G   U
AUC^        -  G          UA        - UAU
80        70         60        50
```

mml-miR-106a(ψ)

CCTTGGCCATGTAAAAGTGCTTACAGTGCAGGTAGCTTTTTGAGATCTACTGCAATGcAA
GCACTTCTTACATTACCATGG

```
        10         20        30        40
  U   CC      -|       A  G     G   C  UU
CC UGG  AUGUAA AAGUGCUU CA UGCAG UAG UU  \
GG ACC  UACAUU UUCACGAA GU ACGUC AUC AG  U
  U   AU      C^       c  A     -   U  AG
80        70        60        50
```

mml-miR-106b(ψ)

CCTGCtGGGGCTAAAGTGCTGACAGTGCAGATAGTGGTCCTCTCCGTGCTACCGCACTGT
GGGTACTTGCTGCTCCAGCAGG

```
        10         20        30           40
           UA-       G       AGA    --| UC
CCUGCuGGGGC   AAGUGCU ACAGUGC   UAGU  GG  C
GGACGACCUCG   UUCAUGG UGUCACG   AUCG  CC  U
           UCG       G       CC-    UG^ UC
 80        70        60         50
```

mml-miR-107(ψ)

CTCTCTGCTTTCAGCTTCTTTACAGTGTTGCCTTGTGGCATGGAGTTCAAGCAGCATTGT
ACAGGGCTATCAAAGCACAGA

```
        10          20        30        40
C   C      C--|   U  U           C   U   AU
 UCU UGCUUU   AGCU CU UACAGUGUUGC UUG GGC  \
 AGA ACGAAA   UCGG GA AUGUUACGACG AAC UUG  G
-   C      CUA^   -  C           -   -   AG
 80        70          60          50
```

mml-miR-122a

CCTTAGCAGAGCTGTGGAGTGTGACAATGGTGTTTGTGTCTAAACTATCAAACGCCATTA
TCACACTAAATAGCTACTaCTAGGC

```
         10        20        30        40
C     C  -|     GG       C           UGUCU
 CUUAG AG AGCUGU  AGUGUGA AAUGGUGUUUG     A
 GGAUC UC UCGAUA  UCACACU UUACCGCAAAC     A
C     a  A^     AA       A           UAUCA
    80        70        60        50
```

mml-miR-124a-1(ψ)

ATCAAGATcAGAGGCTCTGCcCTCCGTGTTCACAGCGGACCTTGATTTAA
TGTCATACAATTAAGGCACGCGGTGAATGCCAAGAGCGGAGCCTACGGCT
GCACTTGAA

```
        10            20        30        40        50
A     AU   ----|        C  CC        A   GA        UAAUG
 UCAAG  cAG    AGGCUCUGC cU  GUGUUCAC GCG  CCUUGAUU     \
 AGUUC  GUC    UCCGAGGCG GA  CGUAAGUG CGC  GGAAUUAA     U
A     AC   GGCA^        A  AC        G   AC        CAUAC
       100        90        80        70        60
```

mml-miR-124a-2

AGGCCTCTCTCTCCGTGTTCACAGCGGACCTTGATTTAAATGTCCATACA
ATTAAGGCACGCGGTGAATGCCAAGAATGGGGCTG

```
        10        20        30         40
A|     UC   CC        A   GA        UAAAUG
 GGCCUC  UCU  GUGUUCAC GCG  CCUUGAUU      \
 UCGGGG  AGA  CGUAAGUG CGC  GGAAUUAA      U
G^     UA   AC        G   AC        CAUACC
    80        70        60        50
```

mml-miR-125a

TGCCAGTCTCTgGGTCCCTGAGACCCTTTAACCTGTGAGGACATCCAGGGTCACAGGTGA
GGTTCTTGGGAGCCTGGCGTCTGGCC

```
        10        20        30            40
```

```
U     UCU Ug  UC  UG   C    UA        ----| A
 GCCAG   C  GG  CC  AGA CCUU  ACCUGUGA    GG C
 CGGUC   G  CC  GG  UCU GGAG  UGGACACU    CC A
C     UGC GU  GA  GU   U    --        GGGA^ U
     80        70        60           50
```

mml-miR-125b-1(ψ)

TGCGCTCCTCTCAGTCCCTGAGACCCTAACTTGTGATGTTTACCGTTTAAATCCACGGGT
TAGGCTCTTGGGAGCTGCGAGTCGTGCT

```
        10        20        30        40
U|   UC   U   UC  UG   C           AU     CC
 GCGC  CUC CAG  CC  AGA CCUAACUUGUG  GUUUA  \
 CGUG  GAG GUC  GG  UCU GGAUUGGGCAC  UAAAU  G
U^   CU   C   GA  GU   C           C-     UU
       80        70        60         50
```

mml-miR-125b-2(ψ)

ACCAGACTTTTCCTAGTCCCTGAGACCCTAACTTGTGAGGTATTTTAGTAACATCACAAG
TCAGGCTCTT GGGACCTAGG CGGAGGGGA

```
        10        20        30        40
A| AGA   UU     UC  UG   C   A        GG-   U
 CC   CUU  CCUAG  CC  AGA CCU ACUUGUGA   UAU U
 GG   GAG  GGAUC  GG  UCU GGA UGAACACU   AUG U
A^ G--   GC     CA  GU   C   C        ACA   A
          80        70        60        50
```

mml-miR-126

CGCTGGTGATGGGACATTATTACTTTTGGTACGCGCTGTGACACTTCAAACTCGTACCGT
GAGTAATAATGCGCtGTCCACaGCA

```
        10        20        30        40
C|   GU     GA           U       CGCUG   C
 GCUG  GAUGG  CAUUAUUACUU UGGUACG     UGA A
 CGAC  CUGuC  GUAAUAAUGAG GCCAUGC     ACU C
A^   AC     GC           U       UCAA-   U
    80        70        60        50
```

mml-miR-126*:

```
        10        20        30        40
C|   GU     GA           U       CGCUG   C
 GCUG  GAUGG  CAUUAUUACUU UGGUACG     UGA A
 CGAC  CUGuC  GUAAUAAUGAG GCCAUGC     ACU C
A^   AC     GC           U       UCAA-   U
    80        70        60        50
```

mml-miR-127(ψ)

TGTGATCACTGTCTCCAGCCTGCTGAAGCTCAGAGGGCTCTGATTCAGAA
AGATCATCGGATCCGTCTGAGCTTGGCTGGTCGGAAGTCTCcTCATC

```
          10        20        30        40
U    UC--|  GUC   A   U   G         G  C      --  AG
 GUGA    ACU   UCC GCC GCU AAGCUCAGA GG UCUGAU  UC  \
 UACU    UGA   AGG UGG CGG UUCGAGUCU CC AGGCUA  AG  A
C    cCUC^  ---   C   U   -         G  U      CU  AA
      90           80         70        60        50
```

mml-miR-128a(ψ)

TGAGCTGTTGGATTCGGGGCCGTAGCACTGTCTGAGAGGTTTACATTTCT

CACAGTGAACCGGTCTCTTTTTCAGCTGCTTC

```
        10        20        30        40
U|   U      UUC       UAG      CU       U
 GAGC GUUGGA   GGGGCCG   CACUGU  GAGAGGU U
 UUCG CGACUU   CUCUGGC   GUGACA  CUCUUUA A
C^   U      UUU       CAA      --       C
 80        70        60          50
```

mml-miR-128b

TGTGCAGTGGGAAGGGGGGCCGATACACTGTACGAGAGTGAGTAGCAGGTCTCACAGTGA
ACCGGTCTCTTTCCCTACTGTGTC

```
        10        20         30         40
U|UG       A          AUA      AC    G-  AG
 G  CAGUGGG AGGGGGGCCG   CACUGU  GAGA  UG  \
 U  GUCAUCC UUUCUCUGGC   GUGACA  CUCU  AC  U
C^GU       C          CAA      --    GG  GA
   80        70        60          50
```

mml-miR-129-1

GGATCTTTTTGCGGTCTGGGCTTGCTGTTCCTCTCAACAGTAGTCAGGAA
GCCCTTACCCCAAAAAGTATCT

```
         10        20        30
    -|      C   CU      G   UUCCU  C
GGAU CUUUUUG GGU  GGGCUU CUG     CU A
UCUA GAAAAAC CCA  CCCGAA GAC     GA A
    U^      C   UU      G   UGAU-  C
 70        60        50         40
```

mml-miR-130a(ψ)

TGCTGCTGGCCAGAGCTCTTTTCACATTGTGCTACTGTCTGCACCTGTCACTAGCAGTGC
AATGTTAAAAGGGCATTGGCCGTGTAGTG

```
        10        20        30        40
U     -|      A        C      UG  A   UCU  A
 GCUGC UGGCCAG GCUCUUUU ACAUUG  CU CUG   GC C
 UGAUG GCCGGUU CGGGAAAA UGUAAC  GA GAU   UG C
G     U^      A        U      GU  C   CAC  U
        80        70        60        50
```

mml-miR-130b

GGCCTGCCCGACACTCTTTCCCTGTTGCACTACTgTgGGCCaCTGGGAAGCAGTGCAATG
ATGAAAGGGCATCGGTCAGGTC

```
        10        20        30        40
|     C    CA       CC         ACU  gGG  a
GGCCUG CCGA  CUCUUUC  UGUUGCACU   gU   CC C
CUGGAC GGCU  GGGAAAG  GUAACGUGA   CG   GG U
^     U    AC       UA         ---  AA-  G
 80        70        60           50
```

mml-miR-132

CCGCCCCCGCGTCTCCAGGGCAACCGTGGCTTTCGATTGTTACTGTGGGAACTGGAGGTA
ACAGTCTACAGCCATGGTCGCCCCGCAGCACGCCCACGCGC

```
        10         20        30        40        50
C   CCCC    -| CCA    A         UUC          GUGGG
 CGC    GCGU CU   GGGC ACCGUGGCU   GAUUGUUACU     A
 GCG    CGCA GA   CCCG UGGUACCGA   CUGACAAUGG     A
C   CACC    C^ CGC    C         CAU          AGGUC
 .        90        80        70        60
```

mml-miR-133a-1(ψ)

ACAATGCTTTGCTAGAGCTGGTAAAATGGAACCAAATCGCCTCTTCAATGGATTTGGTCC
CCTTCAACCAGCTGTAGCTATGCATTGA

```
        10        20        30        40
A|     UUU    G       AA  U  A         GCCUC
 CAAUGC   GCUA AGCUGGU  AA GG ACCAAAUC     U
 GUUACG   CGAU UCGACCA  UU CC UGGUUUAG     U
A^     UAU    G       AC  C  C         GUAAC
       80        70        60        50
```

mml-miR-133a-2

GGGAGCCAAATGCTTTGCTAGAGCTGGTAAAATGGAACCAAATCGACTGT
CCAATGGATTTGGTCCCCTTCAACCAGCTGTAGCTGTGCATTGATGGCGC
CG

```
          10        20        30        40        50
G  A    --|    UUU    G       AA  U  A         GACUG
 GG GCCA  AAUGC   GCUA AGCUGGU  AA GG ACCAAAUC     U
 CC CGGU  UUACG   CGAU UCGACCA  UU CC UGGUUUAG     C
G  G    AG^    UGU    G       AC  C  C         GUAAC
100        90        80        70        60
```

mml-miR-133b

CCTCAGAAGAAAGATGCCCCCTGCTCTGGCTGGTCAAACGGAACCAAGTCCGTCTTCCTG
AGAGGTTTGGTCCCCTTCAACCAGCTACAGCAGGGCTGGCAATtCCCAGTCCTTGGAGA

```
        10              20        30        40        50
C   A  A  AA--  --    --|      C        CA  C  A      U  G    C
 CUC GA GA    GA  UGCC  CCCUGCU UGGCUGGU  AA GG ACCAAG CC UCUU \
 GAG UU CU    Cu  ACGG  GGGACGA AUCGACCA  UU CC UGGUUU GG AGAG C
A   G  C  GACC  UA    UC^      C        AC  C  C      -  -    U
       110       100        90        80        70          60
```

mml-miR-134

CAGGGTGTGTGACTGGTTGACCAGAGGGGCgTGCACTGTGTTCACCCTGTGGGCCACCTA
GTCACCAACCCTC

```
        10        20         30
C     GU        U  A-| G    GCgU  AC
 AGGGU  GUGACUGG UG  CCA AGGG    GC  \
 UCCCA  CACUGAUC AC  GGU UCCC    UG  U
C     AC        C  CG^ G    ACU-  UG
  70        60        50         40
```

mml-miR-135a-1(ψ)

AGGCCTCGCTGTTCTCTATGGCTTTTTATTCCTATGTGATTCTACTGCTCACTCATATAG
GGATTGGAGCCGTGGCGCACGGCGGGGACA

```
        10        20        30        40
AGG|        UCU         UU             UUCUAC
   CCUCGCUGU   CUAUGGCUUU  AUUCCUAUGUGA      \
   GGGGCGGCA   GGUGCCGAGG  UAGGGAUAUACU      U
ACA^        CGC         U-             CACUCG
.        80        70         60        50
```

mml-miR-135a-2

AGATAAATTCACTCTAGTGCTTTATGGCTTTTTATTCCTATGTGATAGTA
ATAAAGTCTCATGTAGGGATGGAAGCCATGAAATACATTGTGAAAAATCA

```
        10        20        30        40        50
A|  AAA     UCUA   C            U            UAGUAA
```

```
 GAU   UUCAC    GUG UUUAUGGCUUUU AUUCCUAUGUGA      \
 CUA   AAGUG    CAU AAGUACCGAAGG UAGGGAUGUACU      U
A^  AA-     UUA-   A            -            CUGAAA
.         90         80         70        60
```

mml-miR-135b

CACTCTGCTGTGGCCTATGGCTTTTCATTCCTATGTGATTGCTGTCCCAAACTCATGTAG
GGCTAAAAGCCATGGGCTACAGTGAGGGGCGAGCTCC

```
                10        20        30          40
CA-------    -                   CAU          --|  CU
         CUCU GCUGUGGCCUAUGGCUUUU   UCCUAUGUGA  UUG  G
         GGGA UGACAUCGGGUACCGAAAA   GGGAUGUACU  AAC  U
CCUCGAGCG    G                   UC-          CA^  CC
      90        80        70         60        50
```

mml-miR-136

TTGGATGAGCCCTCGGAGGACTCCATTTGTTTTGATGATGGATTCTTATGCTCCATCATC
GTCTCAAATGAGTCTTCAGAGGGTTCTATCAT

```
         10        20        30        40
UUG   -|       G        C     UUU          UUCU
   GAU GAGCCCUC GAGGACUC AUUUG   UGAUGAUGGA    \
   CUA CUUGGGAG CUUCUGAG UAAAC   GCUACUACCU    U
UA-   U^       A        -     UCU          CGUA
  90        80        70         60        50
```

mml-miR-137

GGTCCTCTGACTCTCTTCGGTGACGGGTATTCTTGGGTGGATAATACGGA
TTACGTTGTTATTGCTTAAGAATACGCGTAGTCGAGGAGAGTACCAGCGG
CA

```
          10        20        30        40         50
G U  U   --|          UG   G             G      -  GA
 G CC CUG  ACUCUCUUCGG  ACG GUAUUCUUGGGUG AUAAUA CG  \
 C GG GAC  UGAGAGGAGCU  UGC CAUAAGAAUUCGU UAUUGU GC  U
A -  C   CA^          GA   G             -      U  AU
 100        90        80        70         60
```

mml-miR-138-2

CGTTGCTGCAGCTGGTGTTGTGAATCAGGCCGACGAGCAGCGCATCCTCTTACCCGGCTA
TTTCACGACACCAGGGTTGCATCA

```
        10        20        30         40
CGU|  U  AG             UCA     AC-   C  CG
   UGC GC  CUGGUGUUGUGAA   GGCCG   GAG AG  C
   ACG UG  GACCACAGCACUU   UCGGC   UUC UC  A
ACU^  U  G-             UA-     CCA   -  CU
   80         70        60         50
```

mml-miR-139

GTGTATTCTACAGTGCACGTGTCTCCAGTGTGGCTCGGAGGCTGGAGACGCGGCCCTGTT
GGAGTAAC

```
         10        20        30
GUG      -|   U  A            GUGGC
   UAUUCU ACAG GC CGUGUCUCCAGU     \
   AUGAGG UGUC CG GCGCAGAGGUCG     U
CA-      U^   C  -            GAGGC
        60         50        40
```

mml-miR-140

TGTGTCTCTCTCTGTGTCCTGCCAGTGGTTTTACCCTATGGTAGGTTACGTCATGCTGTT
CTACCACAGGGTAGAACCACGGACAGGATACCGGGGCACC

```
        10        20         30        40        50
U|      UCUCU        -  A             A       UU   UC
 GUGUCUC     GUGUCCUG CC GUGGUUUUACCCU UGGUAGG  ACG  A
 CACGGGG     CAUAGGAC GG CACCAAGAUGGGA ACCAUCU  UGU  U
C^      C----        A  -             C       --   CG
.            90         80        70        60
```

mml-miR-141(ψ)

GGCCGGCCCTGGGTCCATCTTCCAGTACAGTGTTGGATGGTCTAATTGTG
AAGCTCCTAACACTGTCTGGTAAAGATGGCcCCCGGGTcGGTT

```
        10        20          30         40
         U   U       --|   U        -   UGG  UAA
GGCCGGCCC GGG CCAUCUU  CCAG ACAGUGUU GGA   UC   \
UUGGcUGGG CCc GGUAGAA  GGUC UGUCACAA CCU   AG   U
         C   C       AU^   -        U   CGA  UGU
  90        80        70         60        50
```

mml-miR-142-5p

GACAGTGCAGTCACCCATAAAGTAGAAAGCACTACTAACAGCACTGGAGGGTGTAGTGTT
TCCTACTTTATGGATGAGTGTACTGTG

```
        10        20        30           40
G        G   C           A         UAA---| G
 ACAGUGCA UCA CCAUAAAGUAG AAGCACUAC      CA C
 UGUCAUGU AGU GGUAUUUCAUC UUUGUGAUG      GU A
G        G   A           C         UGGGAG^ C
      80        70        60        50
```

mml-miR-142-3p

```
        10        20        30        40
G        G   C           A       ACUAACA|    G
 ACAGUGCA UCA CCAUAAAGUAG AAGCACU       GCACU G
 UGUCAUGU AGU GGUAUUUCAUC UUUGUGA       UGUGG A
G        G   A           C       -------^    G
      80        70        60               50
```

mml-miR-143

GCGCAGCGCCgTGTCTCCCAGCCTGAGGTGCAGTGCTGCATCTCTGGTCAGTTGGGAGTC
TGAGATGAAGCACTGTAGCTCAGGAAGAGAGAAGTTGTTCTGCAGC

```
          10        20        30        40        50
GC-    C  CgUG    CCAG      G         G      U  -| AG
   GCAG GC    UCUC    CCUGAG UGCAGUGCU CAUCUC GG UC  U
   CGUC UG    AGAG    GGACUC AUGUCACGA GUAGAG CU AG  U
CGA    U  UUGA    AGAA      G         A      U  G^ GG
    100        90        80        70        60
```

mml-miR-144

TGGGGCCCTGGCTGGGATATCATCATATACTGTAAGTTTGtGATGAGACACTACAGTATA
GATGATGTACTAGTCCGGGCACCCCC

```
          10        20        30         40
U-   -|   U      G        A         A-    Gu
  GGG GCCC GGCUGG AUAUCAUC UAUACUGUA  GUUU  G
  CCC CGGG CUGAUC UGUAGUAG AUAUGACAU  CAGA  A
CC   A^   C      A        -         CA    GU
     80        70         60        50
```

mml-miR-145(ψ)

CACCTTGTCCTCACGGTCCAGTTTTCCCAGGAATCCCTTAaATGCTAAGATGGGGATTCC
TGGAAATACTGTTCTTGAGGTCATGGTT

```
        10        20        30        40
C|  U  U     C  UC    U  C            UAaAUG
 ACC UG CCUCA GG  CAGU UU CCAGGAAUCCCU      \
 UGG AC GGAGU UC  GUCA AA GGUCCUUAGGGG      C
U^  U  U     -  UU    U  A            UAGAAU
       80         70        60        50
```

mml-miR-146a

CCtATGTGTATCCTCAGCTTTGAGAACTGAATTCCATGGGTTGTGTCAGTGTCAGACCTg
TGAAATTCAGTTCTTCAGCTGGGATATCTCTGTCgTCGT

```
             10        20        30        40
CCUAUGU-----|     U     UU            C        GUGUC
            GUAUCC CAGCU  GAGAACUGAAUU CAUGGGUU     A
            UAUAGG GUCGA  UUCUUGACUUAA GUgUCCAG     G
UGCUgCUGUCUC^     -     C-            A        ACUGU
        90         80         70        60        50
```

mml-miR-146b

CCTGGCACTGAGAACTGAATTCCATAGGCTGTGAGCTCTAGCAATGCCCTGTGGACTCA
GTTCTGGTGCCCGG

```
        10        20        30
CCU|     G        AU        CU  GA  U
   GGCACU AGAACUGA  UCCAUAGG  GU  GC C
   CCGUGG UCUUGACU  AGGUGUCC  UA  CG U
GGC^     -        C-        CG  A-  A
  70         60         50         40
```

mml-miR-147

AATCTAAAGAaAACATTTCTGCACACACACCAGACTATtGAAGCCAGTGT
GTGGAAATGCTTCTGCTAcATT

```
         10        20        30
AAUCUAA-|   AA      UG     ACACCAGAC
        AGAa  CAUUUC  CACAC         U
        UCUU  GUAAAG  GUGUG         A
UUAcAUCG^   C-      GU     ACCGAAGtU
 70        60         50        40
```

mml-miR-147b

TATAAATCTAGTGGAAACATTTCcGCACAAACTAGATTCTGGACACCAGT
GTGCGGAAgTGCTTCTGCTgCATTTTTAGG

```
          10        20         30       40
--| U    C        A            AACUAGAUU    A
  UA AAAU UAGUGGAA CAUUUCcGCACA         CUGG \
  AU UUUA gUCGUCUU GUgAAGGCGUGU         GACC C
GG^ U    C        C            ---------    A
.        70        60        50
```

mml-miR-148a

GAGGCAAAGTTCTGAGACACTCCGACTCTGAGTATGATAGAAGTCAGTGC
ACTACAGAACTTTGTCTC

```
        10          20         30
              -  A-|   CC    -   AGU
GAGGCAAAGUUCUG AG  CACU  GACU CUG   \
CUCUGUUUCAAGAC UC  GUGA  CUGA GAU   A
```

```
              A  AC^   --    A   AGU
       60        50          40
```

mml-miR-148b

CAAGCACGATTAGCATTTGAGGTGAAGTTCTGTTATACACTCAGGCTGTGGCTCTCTGAA
AGTCAGTGCATCACAGAACTTTGTCTCGAAAGCTTTCTA

```
        10        20        30        40        50
CAAGCAC| UU   A       UG         U  A    CA    GUGGCU
       GA  AGC UUUGAGG  AAGUUCUGU AU CACU  GGCU      \
       CU  UCG AAGCUCU  UUCAAGACA UA GUGA  CUGA      C
AU-----^ U-   A       GU         C  C    --    AAGUCU
              90        80        70          60
```

mml-miR-149

GCCGGCGCCCaAGCTCTGGCTCCGTGTCTTCACTCCCGTGtTTGTCCGAGGAGGGAGGGA
GGGACGGGGGCTGTGCTGGGGCAGCcGGA

```
         10        20         30        40
G     -|   a   UC       -  G     A     GUG   GU
 CCGGC GCCC AGC  UGGCUCC GU UCUUC CUCCC   uUU  \
 GGcCG CGGG UCG  GUCGGGG CA GGAGG GAGGG   GGA  C
A     A^   G   U-       G  G     -     A--   GC
        80         70        60           50
```

mml-miR-150

CTCCCCATGGCCCTGTCTCCCAACCCTTGTACCAGTGCTGGGCTCAGACCCTGGTACAGG
CCTGGGGGACAGGGACCTGGGGAC

```
        10         20        30         40
C|     U  -            ACC          UG-   G
 UCCCCA GG CCCUGUCUCCCA   CUUGUACCAG   CUG G
 AGGGGU CC GGGACAGGGGGU   GGACAUGGUC   GAC C
C^     -  A            CC-          CCA   U
   80         70         60        50
```

mml-miR-151

TTTCCTGCCCTCGAGGAGCTCACAGTCTAGTATGTCTCATCCCCTACTAGACTGAAGCTC
CTTGAGGACAGGGATGGTCATACTCACCTC

```
          10        20        30
.-U|     C            CA         UGUCU
   UUCCUG CCUCGAGGAGCU  CAGUCUAGUA     C
   AGGGAC GGAGUUCCUCGA  GUCAGAUCAU     A
\ -^     A            A-         CCCCU
      70        60         50        40
```

mml-miR-152

TGTCCCCCCCGGCCCAGGTTCTGTGATACACTCCGACTCGGGCTCTGGAGCAGTCAGTGC
ATGACAGAACTTGGGCCCGGAAGGACC

```
        10        20        30        40
U|   CC  C               G  A    CC    CGG   C
 GUCC  CC GGCCCAGGUUCUGU AU CACU  GACU   GCU U
 CAGG  GG CCGGGUUCAAGACA UA GUGA  CUGA   CGA G
C^   AA  C               G  C    --    ---   G
      80        70        60             50
```

mml-miR-153-1(ψ)

CTCACAGCTGCCAGTGTCATTTTTGTGATCTGCAGCTAGTATTCTCACTC
CAGTTGCATAGTCACAAAAGTGATCATTGGCAGGTGTGGC

```
        10         20        30           40
C     G         -             C-       --|  AU
```

```
 UCACA CUGCCAGUG UCAUUUUUGUGAU  UGCAGCU  AGU  U
 GGUGU GACGGUUAC AGUGAAAACACUG  ACGUUGA  UCA  C
C     G         U             AU       CC^  CU
.        80        70        60        50
```

mml-miR-153-2(ψ)

AGCGGTGGCCAGTGTCATTTTTGTGATGTTGCAGCTAGTAATATGAGCCC
AGTTGCATAGTCACAAAAGTGATCATTGGAAACTGTG

```
        10        20         30         40
A     GG      -             GU       A-| AAU
 GCGGU  CCAGUG UCAUUUUUGUGAU  UGCAGCU  GU   \
 UGUCA  GGUUAC AGUGAAAACACUG  ACGUUGA  CG   A
G     AA      U             AU       CC^ AGU
      80        70        60        50
```

mml-miR-154

GaGGTACTTGAAGATAGGTTATCCGTGTTGCCTTCGCTTTATTTGTGACG
AATCATACACGGTTGACCTATTTTTCAGTACCAA

```
        10        20         30          40
Ga      U            U      -  CCU--|   UUU
  GGUACU GAAGAUAGGUUA CCGUGU UG     UCGC   \
  CCAUGA UUUUUAUCCAGU GGCACA AC     AGUG   A
AA      C            U      U  UAAGC^   UUU
   80        70        60        50
```

mml-miR-154*

```
        10        20         30          40
Ga      U            U      -  CCU--|   UUU
  GGUACU GAAGAUAGGUUA CCGUGU UG     UCGC   \
  CCAUGA UUUUUAUCCAGU GGCACA AC     AGUG   A
AA      C            U      U  UAAGC^   UUU
   80        70        60        50
```

mml-miR-155

CTGTTAATGCTAATCGTGATAGGGGTTTTTACCTCCAACTGACTCCTACATgTTAGCATT
AACAG

```
        10        20        30
|            U    A        UUUACC
CUGUUAAUGCUAA CGUG UAGGGGUU      \
GACAAUUACGAUU gUAC AUCCUCAG      U
^            -    -        UCAACC
    60         50         40
```

mml-miR-181a-1(ψ)

TGAGTTTTGAGGTTGCTTCAGTGAACATTCAACGCTGTCGGTGAGTTTGGAATTAAAATC
AAAACCATCGACCGTTGATTGTACCCTATGGCTAACCATCATCTACTCCA

```
          10          20        30        40        50
U-    UU-   -    -|  UC  UGA   U      CU       A    G  AUU
  GAGU   UGA GGUU GCU  AG   ACA UCAACG  GUCGGUG GUUU GA   \
  CUCA   ACU CCAA CGG  UC   UGU AGUUGC  CAGCUAC CAAA CU   A
AC    UCU   A    U^  UA  CCA   U      --       -    A  AAA
.       100        90        80          70        60
```

mml-miR-181a-2(ψ)

AGAAGGGCTATCAGGCCAGCCTTCAGAGGACTCCAAGGAACATTCAACGC
TGTCGGTGAGTTTGGGATTTGAAAAAACCACTGACCGTTGACTGTACCTc

```
GGGGTCCTTA
      30        40        50        60
  A         A   A   U      CU        A    GGGAU
   GAGGACUCC AGG ACA UCAACG  GUCGGUG GUUU     \
   UUCCUGGGG UCC UGU AGUUGC  CAGUCAC CAAA     U
  A         c   A   C      --        -    AAAGU
110       100        90          80         70
```

mml-miR-181b-1(ψ)

```
CCTGTGCAGAGATTATTTTTAAAAGGTCACAATCAACATTCATTGCTGT
CGGTGGGTTGAACTGTGTaGACAAGCTCACTGAACAATGAATGCAACTGT
GGCCCCGCTT
                30        40        50        60
         AAAA       AUCAA         CUG          GAA  G
             GGUCACA     CAUUCAUUG   UCGGUGGGUU   CU U
             CCGGUGU     GUAAGUAAC   AGUCACUCGA   Ga G
         CC--       CAAC-         A--          ACA  U
               100         90          80        70
```

mml-miR-181b-2

```
CTGATGGCTGCACTCAACATTCATTGCTGTCGGTGGGTTTGAGTCTGAATCAACTCACTG
ATCgATGAATGCAAACTGCGGACCAAACA
         10        20        30        40
CUGA   -|   CUCAA        CU            U  GUC
    UGG CUGCA     CAUUCAUUG  GUCGGUGGGUU GA   \
    ACC GGCGU     GUAAGUAgC  UAGUCACUCAA CU   U
ACAA   A^     CAAAC        --           -  AAG
        80        70          60          50
```

mml-miR-181C(ψ)

```
CGGAgAATTTGCCAAGGGTTTGGGGGAACATTCAACCTGTCGGTGAGTTTGGGCAGCTCA
GGCAAACCATCGACCGTTGAGTGGACCCTGAGaCCTGGAcTTGCCATCCT
        10         20        30        40        50
C   gAAUUUG-|  AG     G    AA        CU        A     ---  C
 GGA        CCA  GGUUU GGGG  CAUUCAAC  GUCGGUG GUUUG   GG A
 CCU        GGU  CCaGA UCCC  GUGAGUUG  CAGCUAC CAAAC   CU G
U   ACCGUUcA^  --     G    AG        C-        -     GGA  C
.       100          90        80         70         60
```

mml-miR-181d

```
GTCCCCTCCCCTAGGCCACAGCCaAGGTCACAATCAACATTCATTGTTGTCGGTGGGTTG
TGAGGACcGAGGCCAGACCCACCGGGGGATGAATGTCACTGTGGCTGGGCCAGACACGGC
TTAAGGGGAATGGGGAC
          10         20            30        40        50        60
      --     -      ACA----|  aA        AUCA         G UG          GUGA
ACc
GUCCCC  UCCCC UAGGCC       GCC  GGUCACA    ACAUUCAUU U  UCGGUGGGUU
GG   \
CAGGGG  AGGGG AUUCGG       CGG  UCGGUGU    UGUAAGUAG G  GGCCACCCAG
CC   G
      UA     A      CACAGAC^  G-        CAC-         G --          A---
GGA
     130       120       110        100          90          80
70
```

mml-miR-182(ψ)

```
AGCTGCTTGCCTCCCCCtGTTTTTGGCAATGGTAGAACTCACACTGGTGAGGTAAtgGGAT
```

CCGGTGGTTCTAGACTTGCCAACTAcGGGGCGAGGgCTCAGCCGGCAC

```
            10        20        30        40        50
A-   -   U- -|   C      UU       UGG      UCA      UGAGGU
  GCU GCU  G CCUC CCCUGU  UUGGCAA   UAGAAC   CACUGG      A
  CGG CGA  C GGAG GGGGCA  AACCGUU   AUCUUG   GUGGCC      A
CA   C   CU g^   C      UC       CAG      ---      UAGGGU
       100        90        80        70           60
```

mml-miR-182*

```
            10        20        30        40        50
A-   -   U- -|   C      UU       UGG      UCA      UGAGGU
  GCU GCU  G CCUC CCCUGU  UUGGCAA   UAGAAC   CACUGG      A
  CGG CGA  C GGAG GGGGCA  AACCGUU   AUCUUG   GUGGCC      A
CA   C   CU g^   C      UC       CAG      ---      UAGGGU
       100        90        80        70           60
```

mml-miR-183(ψ)

CCGCAGAacGTGACTCCTGTTCTGTGTATGGCACTGGTAGAATTCACTGTGAACAGTCTC
gGTCAGTGAATTACCGAAGGGCCATAAACAGAGCAGAGACAGATCCACGA

```
        10             20        30          40          50
CCGCAGAa    AC----  -         G      AC--|    GA        --   AC
        cGUG      UC CUGUUCUGU UAUGGC    UGGUA  AUUCACUG  UGA  A
        GCAC      AG GACGAGACA AUACCG    GCCAU  UAAGUGAC  gCU  G
A-------    CUAGAC  A         A      GGAA^    --        UG   CU
.              100        90        80          70        60
```

mml-miR-184

TCAGTCACGTCCCCTTATCACTTTTCCAGCCCAGCTTTaTGACTGTAAGTGTTGGACGGA
GAACTGATAAGGGTAGGTGATTGA

```
        10        20        30         40
|       GUC         C      AGC     -     G
UCAGUCAC   CCCUUAUCA UUUUCC   CCAGC UUUaU A
AGUUAGUG   GGGAAUAGU AAGAGG   GGUUG GAAUG C
^       GAU         C      CA-     U     U
   80        70        60         50
```

mml-miR-185

AGGGcGCGAGGGATTGGAGAGAAAGGCAGTTCCTGATGGTCCCCTCCtCAGGG
GCTGGCTTTCCTCTGGTCCTTCCCTCCCA

```
        10        20        30        40
A|  cGC            A     G          U  UC
 GGG   GAGGGAUUGGAG GAAAG CAGUUCCUGA GG  \
 CCC   CUUCCUGGUCUC CUUUC GUCGGGGACu CU  C
A^  UCC            -     G          C  CC
 80        70         60        50
```

mml-miR-186

TGCTTGTAACTTTCCAAAGAATTCTCCTTTTGGGCTTTCTGGTTTTATTTTAAGCCCAAA
GGTGAATTTcTTGGGAAGTTTGAGCT

```
        10        20        30        40
U|    U          A      UC           UCU   U
 GCUUG AACUUUCCAA GAAUUC  CUUUUGGGCUU   GGU \
 CGAGU UUGAAGGGUU UUUAAG  GGAAACCCGAA   UUA U
U^    -          c      U-           UU-   U
      80        70         60        50
```

mml-miR-187

GGTCaGGCTCACtATGACACAGTGTGAGACCTCGGGCTACAACACAGGAC
CCGGGtGCTGCTCTGACCCCTCGTGTCTTGTGTTGCAGCCGGAGGGACGC
AGGTCCGCA

```
         10        20        30        40        50
uAU-|  ACA    GAGA    G    A           C    uGCUGC
    GAC   GUGU    CCUC GGCU CAACACAGGAC CGGG      U
    CUG   CGCA    GGAG CCGA GUUGUGUUCUG GCUC      C
ACGC^  GA-    G---    G    C           U    CCCAGU
      90            80        70        60
```

mml-miR-188(ψ)

TGCTCCCTCTCTCACATCCCTTGCATGGTGGAGGGTGAGCTTTaTGAAAA
CCCCTCCCACATGCAGGGTTTGCAGGATGGtGAGCC

```
         10        20        30        40
U    -| UC   CA  UC         GU      UGAGCUU
 GCUC CC  UCU  CA  CCUUGCAUG  GGAGGG       U
 CGAG GG  AGG  GU  GGGACGUAC  CCUCCC       a
C    u^ U-   AC  UU         AC      CAAAAGU
     80          70        60        50
```

mml-miR-190(ψ)

TGCAGGCCTCTGTGTGATATGTTTGATATATTAGGTTGTTATTTAATCCA
ACTATATATCAAACATATTCCTACAGTGTCTTGCC

```
        10         20        30        40
UG    CCU     U-|                UA      UUAU
  CAGG   CUGUG  GAUAUGUUUGAUAUAU  GGUUG     U
  GUUC   GACAU  UUAUACAAACUAUAUA  UCAAC     U
CC    UGU     CC^               --      CUAA
    80        70        60          50
```

mml-miR-190b

TGCTTCTGTGTGATATGTTTGATATTGGGTTGTTTAATTAGGAACCAACT
AAATGTCAAACATATTCTTACAGCAGCtG

```
         10         20         30
U-   U     U-              G    -|   AA
  GCU CUGUG  GAUAUGUUUGAUAUU GGUU GUUU  U
  CGA GACAU  UUAUACAAACUGUAA UCAA CAAG  U
Gu   C     UC               A    C^   GA
        70        60        50        40
```

mml-miR-191

CGGCTGGACAGCGGGCAACGGAATCCCAAAAGCAGCTGTTGTCTCCAGAG
CATTCCAGCTGCGCTTGGATTTCGTCCCCTGCTCTCCTGCCT

```
        10        20        30          40
C   U   C      CA         CAAAA      U--|  CUC
 GGC GGA AGCGGG  ACGGAAUCC     GCAGCUG   UGU   \
 CCG CCU UCGUCC  UGCUUUAGG     CGUCGAC   ACG   C
U   U   C      CC         UUCG-      CUU^  AGA
 90        80        70          60        50
```

mml-miR-191*

```
        10        20        30          40
C   U   C      CA         CAAAA      U--|  CUC
 GGC GGA AGCGGG  ACGGAAUCC     GCAGCUG   UGU   \
 CCG CCU UCGUCC  UGCUUUAGG     CGUCGAC   ACG   C
U   U   C      CC         UUCG-      CUU^  AGA
```

```
 90        80        70         60        50
```

mml-miR-192

GCtGAGACCGAGTGCACAGGGCTCTGACCTATGAATTGACAGCCAGTGCTCTCGTCTCCC
CTCTGGCTGCCAATTCCATAGGTCACAGGTATGTTCGCCTCAATGCCAGC

```
        10          20         30         40        50
    AGACC   U  --   GG   C-         -|     A       UGCUCUC
GCtG     GAG GC  ACA  GCU  UGACCUAU GAAUUG CAGCCAG       G
CGAC     CUC CG  UGU  UGG  ACUGGAUA CUUAAC GUCGGUC       U
    CGUAA   -  CU   A-   AC         C^     C       UCCCCUC
.       100          90         80         70        60
```

mml-miR-193a

GGATGGGAGCTGAGGGCTGGGTCTTTGCGGGCGAGATGAGGGTGTCGGATCAACTGGCCT
ACAAAGTCCCAGTcCTCGGCCCCCG

```
        10        20        30        40
GGAU|  A             U     CG   G  A   GGGU
    GGG GCUGAGGGCUGGG CUUUG  GGC AG UGA    G
    CCC CGGCUCcUGACCC GAAAC  CCG UC ACU    U
G---^  C             U     AU   G  A     AGGC
       80        70        60        50
```

mml-miR-193b

GTGGTCTCAGAATCGGGGTTTTGAGGGCGAGATGAGTTTATGTTTTATCCAACTGGCCCT
CAAAGTCCCGCTTTTGGGGTCAT

```
        10        20        30        40
|  GU       U    GU         G  A  AG UUAU
GUG  CUCAGAA CGGG  UUUGAGGGC AG UG  U    \
UAC  GGGUUUU GCCC  AAACUCCCG UC AC  A    G
^  UG       C    UG         G  A  CU UUUU
  80        70        60        50
```

mml-miR-194-1(ψ)

ATGGTGTTATCAAGTGTAACAGCAACTCCATGTGGACTGTGTACCAATTTCCAGTGGAGA
TGCTGTTACTTTTGATGGTTACCAA

```
         10        20        30        40
A    -|UU      U         A      G    CU  GU
 UGGU G  AUCAAG GUAACAGCA CUCCAU UGGA  GU  A
 ACCA U  UAGUUU CAUUGUCGU GAGGUG ACCU  UA  C
A    U^GG      U         A      -    U-  AC
    80        70        60         50
```

mml-miR-194-2

TGGcTCCCGCCCCCTGTAACAGCAACTCCATGTGGAAGTGtCCACTGaTTCCAGTGGGGC
TGCTGTTATCTGGGGCGAGGGCCgG

```
         10        20        30            40
-      C      CU         A      G    ---|  G
 UGGcUC CGCCCC  GUAACAGCA CUCCAU UGGA   AGU u
 gCCGGG GCGGGG  UAUUGUCGU GGGGUG ACCU   UCA C
G      A      UC         C      -    UaG^  C
    80        70        60         50
```

mml-miR-195

AGCTTCCCTGGCTCTAGCAGCACAGAAATATTGGCACAGGGAAGCaAGTCTGCCAATATT
GGCTGTGCTGCTCCAGGCAGGGTGGTG

```
        10        20         30        40
A   U     G  CU         A-|        CAG  AAG
 GCU CCCUG CU  AGCAGCACAG  AAUAUUGGCA   GG   \
```

```
 UGG GGGAC GA  UCGUCGUGUC  UUAUAACCGU   CU   C
G   U     G  CC          GG^         ---  GAa
      80        70        60            50
```

mml-miR-196a-1(ψ)

GTGAATTAGGTAGTTTCATGTTGTTGGGCCTGGGTTTCTGAACACAACAACATTAAACCA
CCCGATTCAC

```
        10        20        30
|     UA   A    C         GGCCU  G
GUGAAU  GGU GUUU AUGUUGUUG     GG U
CACUUA  CCA CAAA UACAACAAC     UC U
^     GC   C    U         ACAAG  U
.        60        50        40
```

mml-miR-196a-2

TGCTCGCTCAGCTGATCTGTGGCTTAGGTAGTTTCATGTTGTTGGGATTGAGTTTTGAACT
CGGCAACAAGAAACTGCCTGAGTTACATCAGTCGGTTTTCGTCGAGGGC

```
           10          20        30        40        50
UG-    - UC-       C--|                A          A UG G
   CUCG C   AGCUGAU   UGUGGCUUAGGUAGUUUC UGUUGUUGGG U  A \
   GAGC G   UUGGCUG   ACAUUGAGUCCGUCAAAG ACAACGGCUC A  U U
CGG    U CUU       ACU^                A          A GU U
.       100        90        80        70        60
```

mml-miR-196b

ACTGGTCGGTGATTTAGGTAGTTTCCTGTTGTTGGGATCCACCTTTCTCT
CGACAGCACGACACTGCCTTCATTA

```
        10        20        30        40
ACUGGUC|    UUU       U  C        UCCA
       GGUGA   AGGUAGU UC UGUUGUUGGGA    C
       UUACU   UCCGUCA AG ACGACAGCUCU    C
A------^    ---       C  C        CUUU
          70           60         50
```

mml-miR-197

GGCTGTGCCGGGTAGAGAGGGCAGTGGGAGGTAAGAGCTCTTCACCCTTC
ACCACCTTCTCCACCCAGCATGGCC

```
        10        20        30
        C    A       CA     GA   -|  A
GGCUGUGC GGGU GAGAGGG  GUGG  GGU AAG G
CCGGUACG CCCA CUCUUCC  CACU  CCA UUC C
        A    C       AC     UC   C^  U
    70        60        50        40
```

mml-miR-198(ψ)

TCATTGGTCCAGAGGGGAaATAGGTTCCTGTGATTTTTCCTTCTTCTCTg
TAGAATAAATGA

```
           10                  20
     GG-  --          ---------|   G
UCAUU   UC  CAGAGGGGAa         AUAG U
AGUAA   AG  GUCUCUUCUU         UGUC U
     AUA  AU          CCUUUUUAG^   C
 60        50        40        30
```

mml-miR-199a-2

GCCAACCCAGTGTTCAGACTACCTGTTCAGGAGGCTCTCAAcGTGTACAG

```
TAGTCTGCACATTGGTTAGGC
        10        20        30
   AAC       U       C    U  G---|  G
GCC   CCAGUGU CAGACUA CUGU CA    GAG \
CGG   GGUUACA GUCUGAU GACA GU    CUC C
   AUU       C       -    U  GcAA^  U
70        60         50        40
```

mml-miR-199a*

```
        10        20        30
   AAC       U       C    U  G---|  G
GCC   CCAGUGU CAGACUA CUGU CA    GAG \
CGG   GGUUACA GUCUGAU GACA GU    CUC C
   AUU       C       -    U  GcAA^  U
70        60         50        40
```

mml-miR-199a-1(ψ)

```
AGGAAGCTTCTGGAGATCCTGCTCCGTCGCCCCAGTGTTCAGACTACCTG
TTCAGGACAATGCCGTTGTACAGTAGTCTGCACATTGGTTAGACTGGGCA
AGGGAGAGCA
        10        20         30        40        50
AGGAA|  U  GGAGA    -    C   GCC       U        C   UCA--  AC
     GCU CU     UCCU GCUC GUC   CCAGUGU CAGACUAC UGU     GG  A
     CGA GA     GGGA CGGG CAG   GGUUACA GUCUGAUG ACA     CC  A
A----^  -  -----    A    U   AUU       C        -   UGUUG  GU
.                100        90        80          70        60
```

mml-miR-200a*

```
CCGGGCCCCTGTGAGCATCTTACCGGACAGTGCTGGATTTCCCAGCTTGACTCTAACACT
GTCTGGTAACGATGTTCAAAGGTGACCCaC
         10         20        30
CC   CC-   G         -            -----------|    AU
  GGG   CCU UGAGCAUC UUACCGGACAGU            GCUGG  \
  CCC   GGA ACUUGUAG AAUGGUCUGUCA            CGACC  U
Ca   AGU   A         C            CAAUCUCAGUU^    CU
.        80        70         60         50        40
```

mml-miR-200a

```
         10         20        30
CC   CC-   G         -            -----------|    AU
  GGG   CCU UGAGCAUC UUACCGGACAGU            GCUGG  \
  CCC   GGA ACUUGUAG AAUGGUCUGUCA            CGACC  U
Ca   AGU   A         C            CAAUCUCAGUU^    CU
.        80        70         60         50        40
```

mml-miR-200c(ψ)

```
CCCTCGTCTTACCCAGCAGTGTTTGGGTGCGGTTGGGAGTCTCTAATACT
GCCGGGTAATGATGGAGG
         10         20        30
  CU    -|     A        U   U  GGU
CC  CGUC UUACCC GCAGUGUU GGG GC   U
GG  GUAG AAUGGG CGUCAUAA CUC UG   G
  AG    U^     C        U   -  AGG
       60         50         40
```

mml-miR-203

GTGcTGGGGACTCGCGCGCTGGGTCCAGTGGTTCTTAACAGTTCAACAGTTCTGTAGCGC
AATTGTGAAATGTTTAGGACCACTAGACCCGGCGGGCaCGGCGACAGCGA

```
        10        20        30        40        50
G|    GGGACUC  G         C         U    G    A     C  U
 UGcUG       GC CGCUGGGUC AGUGGUUCU AACA UUCA CAGUU UG \
 GCGAC       CG GCGGCCCAG UCACCAGGA UUGU AAGU GUUAA GC A
A^    AGCGGCa  G         A         U    A    -     C  G
.       100        90        80        70         60
```

mml-miR-204

GGCTACAGTCTTTCTTCATGTGACTCGTGGACTTCCCTTTGTCATCCTATGCCTGAGAAT
ATATGAAGGAGGCTGGGAAGGCAAAGGGACGTTCAATTGTCATCACTGGC

```
        10        20        30        40        50        60
GGCUA|   CUUUCUUCAU     UCG     U          A     U    GAGAAUA
     CAGU          GUGAC   UGGAC UCCCUUUGUC UCCUA GCCU       \
     GUCA          UACUG   ACUUG AGGGAAACGG AGGGU CGGA       U
CG---^   C---------     UUA     C          A     -    GGAAGUA
.                   100        90        80         70
```

mml-miR-205

AAAGATCCTCAGgCAATCCATGTGCTTCTCTTGTCCTTCATTCCACCGGAGTCTGTCTCA
TACCCAACCAGATTTCAGTGGAGTGAAGTTCAGGAGGCATGGAGCTGACg

```
        10        20        30        40        50        60
AAAGAUCC|   G AA       GCUU      UC           C        UCUCAU
        UCAG C  UCCAUGU    CUCUUG  CUUCAUUCCAC GGAGUCUG      \
        AGUC G  AGGUACG    GAGGAC  GAAGUGAGGUG CUUUAGAC      A
GC------^   - --       ----      UU           A        CAACCC
.                100            90        80        70
```

mml-miR-206

TGCTTCCCGAGGCCACATGCTTCTTTATATCCCCATATGGATTACTTTGCTATGGAATGT
AAGGAAGTGTGTGGTTTCGGCAAGTG

```
        10        20        30        40
U|   C                        CC     UG  UU
 GCUU CCGAGGCCACAUGCUUCUUUAUAU  CCAUA  GA  \
 UGAA GGCUUUGGUGUGUGAAGGAAUGUA  GGUAU  UU  A
G^   C                        A-     CG  UC
     80        70        60         50
```

mml-miR-208

TGACaGGCGAGCTTTTGGCCCGGGTTATACCTGATGCTCACGTATAAGAC
GAGCAAAAAGCTTGTTGGTCA

```
        10        20        30
U|  aG         G  C  GG      C  AU
 GAC  GCGAGCUUUU GC CG  UUAUAC UG  \
 CUG  UGUUCGAAAA CG GC  AAUAUG AC  G
A^  GU         A  A  AG      C  UC
70        60        50        40
```

mml-miR-208b

CCTCTCAGGGAAGCTTTTTGCTCGAATTATGTTTCTGATCCGAATATAAG
ACGAACAAAAGGTTTGTCTGAGGGCAG

```
          10        20        30
C--|       G          C   AA        CUG
   CUCUCAGG AAGCUUUUUG UCG  UUAUGUUU   \
   GGGAGUCU UUUGGAAAAC AGC  AAUAUAAG   A
GAC^       G          A   AG        CCU
      70        60        50        40
```

mml-miR-210

ACCCGGCAGTcCCTCCAGGCGCAGGGCAGCCCCTGCCCACCGCACACTGC
GCTGCCCCAGACCCACTGTGCGTGTGACAGCGGCTGATCTGTGCCTGGGC
AGCGCGACCC

```
        10         20        30        40         50
ACCC  CA  cCC-           GG     C   CC    -     C  CG-| GC
    GG  GU    UCCAGGCGCAG  CAGCC CUG  CAC CGCACA UG   CU  \
    CC  CG    GGGUCCGUGUC  GUCGG GAC  GUG GCGUGU AC   GA  C
C---  AG  CGAC           UA     C   A-   U      C  CCA^ CC
.          100        90        80         70        60
```

mml-miR-211(ψ)

CTGGCCATGTGACTTGTGGGCTTCCCTTTGTCATCCTTtGCCTAGGGCTCTGAGCAGGGC
AGGGACAGCAAAGGGGTGCTCAGTTGTCACTTCCCACAGCACaGAG

```
                10        20        30        40        50
--   -  CAU-----|    UUG     U         CA      u    AGG  UC
  CUG GC        GUGAC   UGGGC UCCCUUUGU  UCCUU GCCU   GC  \
  GAC CG        CACUG   ACUCG GGGGAAACG  AGGGA CGGG   CG  U
GA   A  ACACCCUU^    UUG     U         AC      -    A--  AG
    100        90        80        70         60
```

mml-miR-212

CGGGGCACCCCGCCCGGACAGCGCGCCGGCACCTTGGCTCTAGACTGCTTACTGCCCGGG
CCGCCCTCAGTAACAGTCTCCAGTCAgGGCCACCGACGCCTGGCCCCGCC

```
         10        20         30        40        50
-| CC   A   C   C   CA-        CU      C      CCC   C
 GC  GG CAG GCG CGG   CCUUGGCU  AGACUG UUACUG   GGG \
 CG  CC GUC CGC GCC   GGgACUGA  UCUGAC AAUGAC   CCC C
C^ CC   G   -   A   ACC        CC      -      U--   G
        90         80        70          60
```

mml-miR-214(ψ)

GGCCTGGCTGGACAGAGTTGTCATGTGTCTGCCTGTCTACACTTGCTGTGCAGAACATCC
GCTCACCTGTACAGCAGGCACAGACAGGCAGTCACATGACAACCCAGCCT

```
        10        20        30        40        50
GGCCU|     ACAGA          U          ACA             AACAUC
     GGCUGG     GUUGUCAUGUG CUGCCUGUCU   CUUGCUGUGCAG      \
     CCGACC     CAACAGUACAC GACGGACAGA   GGACGACAUGUC      C
U----^     -----          U          CAC             CACUCG
.                100        90        80        70
```

mml-miR-215(ψ)

ATCATTAAGAAATGGTATACAGGAAAATGACCTATGAATTGACAGACAcTATAGCTGAGT
TTGTCTGTCATTTCTTTAGGCCAATATTCTGTATGACTGTGCTACTTCAA

```
        10              20        30         40        50
AUCAUUAA   A    ------        AAA  A    U-|  U         cU  AG
        GAA UGGU      AUACAGGA   UG CCUA  GAA UGACAGACA  AU  C
        CUU AUCG      UAUGUCUU   AC GGAU  CUU ACUGUCUGU  UG  U
AA------   C    UGUCAG        AUA  C    UU^  U         U-  AG
              100        90        80        70         60
```

mml-miR-216a

GATGGCTGTGAGTTGGCTTAATCTCAGCTGGCAACTGTGAGATGTTCATACAATCCCTCA
CAGTGGTCTCTGGGATTAcGCTAAACAGAGCAATTTCCTTGCCCTCgCGA

```
                    10          20        30        40
```

```
----| U   --------   GAG--     U         CU   A        A---   U
    GA GGC        UGU     UUGGC UAAUCUCAG  GGC ACUGUGAG    UGU C
    CU CCG        ACG     AAUCG AUUAGGGUC  CUG UGACACUC    ACA A
AGCg^ C   UUCCUUUA   AGACA     c         U-   G        CCUA   U
.       100        90        80        70         60        50
```

mml-miR-216b

GCAGACTGGAAAATCTCTGCAGGCAAATGTGATGTCACTGAaGAAATCAC
ACACTTACCCGTAGAGATTCTACAGTCTGACA

```
          10        20          30
G--       GAA         A  C  A--|    G  AC
   CAGACUG   AAUCUCUGC GG AA   UGUGAU UC  U
   GUCUGAC   UUAGAGAUG CC UU   ACACUA AG  G
ACA       AUC         C  A  CAC^    A  aA
 80        70         60        50        40
```

mml-miR-217

AaTATAATTATTACATAGTTTTTGATGTCGCAGATTCTGCATCAGGAACTGATTGGATAA
GAATCAGTCACCATCAGTTCCTAATGCATTGCCTTCAGCATCTAAACAAG

```
        10         20           30        40        50         60
AaUAUAAUUAUUACAUA    UU     C----|   AUUC     C          UG-   AAG
                 GUUU  GAUGU     GCAG    UGCAU AGGAACUGAU   GAU   A
                 CAAA  CUACG     CGUU    ACGUA UCCUUGACUA   CUG   A
GAA--------------    U-     ACUUC^   ----     A          CCA   ACU
.                      100         90           80        70
```

mml-miR-218-1

AATGTAGCGAGATTTTCTGTTGTGCTTGATCTAACCATGTGGTTGCGAGGTATGAGTAAA
ACATGGTTCCGTCAAGCACCATGGAACGTCACGCAGCTTTCTACA

```
             10        20        30        40        50
AAU-----| A   A   U      U          CU        GG    GAGG
        GU GCG GAU UUCUGU GUGCUUGAU  AACCAUGU  UUGC    \
        CG CGC CUG AAGGUA CACGAACUG  UUGGUACA  AAUG    U
ACAUCUUU^ A   A   C      C          CC        A-    AGUA
   100        90        80        70        60
```

mml-miR-218-2(ψ)

GACCAGTCGCTGCGGGGCTTTCCTTTGTGCTTGATCTAACCATGTGGTGGAACGATGGAA
ACGGAACATGGTTCTGTCAAGCACCGCGGAAAGCACCGTGCTCTCCTGCA

```
          10        20        30        40        50
GAC   UC--| U    G       UUU         CU         GGUGGAACGA  G
   CAG    GC GCGG GCUUUCC   GUGCUUGAU  AACCAUGU           UG A
   GUC    CG UGCC CGAAAGG   CACGAACUG  UUGGUACA           GC A
AC-   CUCU^ -    A       CGC         UC         AG--------  A
.        100         90        80        70                60
```

mml-miR-219-1(ψ)

CCGCCCCGGGCCGCGGCTCCTGATTGTCCAAACGCAATTCTCGAGTCTATGGCTCtGGCCGAG
AGTTGAGTCTGGACGTCCCGAGCCGCCGCCCCCAAACCTCGAGgGGG

```
                   10        20        30        40        50
CCG    -----------|   C       CU   U     A  G         A   UAUG
   CCCC           GGGC GCGGCUC  GAU GUCCA AC CAAUUCUCG GUC    \
   GGgG           CCCG CGCCGAG  CUG CAGGU UG GUUGAGAGC CGG    G
G--    AGCUCCAAACC^   C       CC   -     C  A         -   uCUC
.         100         90        80         70        60
```

mml-miR-219-2

ACTCAGGGGCTTCGCCACTGATTGTCCAAACGCAATTCTTGTACGAGTCTGCGGCCAACC
GAGAATTGTGGCTGGACATCTGTGGCTGAGCTCCGGG

```
        10        20        30         40       50
A|  A       C     U   U     AA           UACGA  CU
 CUC GGGGCUU GCCAC GAU GUCCA  CGCAAUUCUUG     GU  \
 GGG CCUCGAG CGGUG CUA CAGGU  GUGUUAAGAGC     CG  G
-^  -       U     U   -     CG           CAAC-  GC
        90        80         70        60
```

mml-miR-220b

GACAGcGTGGCgTTGTAGGGCTCCACcACCGTgTCcGACACcTTGGGCGA
GGGCAtgAcGCTGAAGGTGTTCATGATGCGGTCcGGGAACTCCTCgCGGA
TCTTgCTGATG

```
         10          20        30          40        50
GA-     UGGCg    -   - C   ACc        c--|       G    AGGG
   CAGcG     UUGU AGG G UCC   ACCGUgUC   GACACcUU GGCG    \
   GUCgU     GGCg UCC C AGG   UGGCGUAG   UUGUGGAA UCGc    C
GUA     UCUA-    C   U A   GcC        UAC^       G    AguA
 .       100         90        80        70        60
```

mml-miR-220a(ψ)

GACAGcGTGGCATTGTAGGGCTCCACcACCaTgTCTGACACTTTGGGtGA
GaGCACCAcGCTGAAGGTGTTCATaATGtGGTCTGGGAACTCCTtgtGGA
TCTTACTGATG

```
         10         20          30          40             50
GA-   cGU| C   G-     C--   ACc       C-   -      G-----    A
   CAG   GG AUU  UAGGG   UCC   ACCaUgU  UGA CACUUU      GGuG G
   GUC   UC UAG  guUCC   AGG   UGGuGUA  ACU GUGGAA      CCAC a
GUA   AU-^ -   Gu     UCA   GUC       aU   U      GUCGcA    G
 .         100        90        80        70        60
```

mml-miR-220c

GACAGcGTGGCATTGTAGGGCTCCACcACtgTgTCTGACACcTTGGGCGA
GGGCACgAcGCTGAAGGTGTTCATGATGCGGTCcGGatACTCCTCACG

```
        10        20        30          40        50
GACAG|    CAUU   GGGC   ACc        -   -      G    AGGG
     cGUGG    GUA    UCC   ACugUgUC UGA CACCUU GGCG    \
     GCACU    CAu    aGG   UGGCGUAG ACU GUGGAA UCGc    C
-----^    CCU-   ----   cC-        U   U      G    AgCA
             90             80        70        60
```

mml-miR-220d

GTGGCgTTGTAGGGCTCCAcCACCGTgTCTGACACcTTGGGtGAGGGCAt
gAcGCTGAAGGTGTTCATGATGCGGTCTGGGtACTCtTCcCGGATCTTgC
TGATG

```
          10               20         30         40        50
G--     UU  A-   C------|   cC        -   -      GGGuGAG   Au
   UGGCg  GU  GGG       UCCA  ACCGUgUC UGA CACcUU       GGC  \
   GUCgU  UA  CcC       GGGU  UGGCGUAG ACU GUGGAA       UCG  g
GUA     UC  GG   UuCUCAu^   C-        U   U      G------   cA
   100        90        80         70        60
```

mml-miR-221(ψ)

TGAACATCCAGGTCTGGGGCATGAACCTGGCATACAATGTAGATTTCTGTGTTCGTTAGG
CAACAGCTACATTGTCTGCTGGGTTTCAGGCTACCTGGAAACATGTTCTC

```
             10        20         30        40        50
U-     ----|      CUGG   A    -  UG   U         AUUU    -   CG
```

```
  GAACA    UCCAGGU    GGC UGAA CC  GCA ACAAUGUAG    CUGU GUU  \
  CUUGU    AGGUCCA    UCG ACUU GG  CGU UGUUACAUC    GACA CGG  U
CU     ACAA^      ----   G    U  GU   C         ----    A   AU
.       100            90        80        70            60
```

mml-miR-222

GCTGCtGGAAGGTATAGGTACCCTCAATGGCTCAGTAGCCAGTGTAGATCCTGTCTTTCG
TAATCAGCAGCTACATCTGGCTACTGGGTCTCTGATGGCATCTTCTAGCT

```
        10        20        30        40         50
GCU|         AUAGGUA  C   AU              -     AUC   UCUUU
   GCuGGAAGGU       CC UCA  GGCUCAGUAGCCAG UGUAG   CUG     \
   CGAUCUUCUA       GG AGU  CUGGGUCAUCGGUC ACAUC   GAC     C
U--^         C------  U   CU              U     GAC   UAAUG
.         100              90        80        70        60
```

mml-miR-223(ψ)

CCcGGCCTCCTGCAGTGCCACGCTCCGTGTATTTGACAAGCTGAGTTGGACACTCCgTGT
GGTAGAGTGTCAGTTTGTCAAATACCCCAAGTGCGGCAtATGCTTACCAG

```
        10        20        30          40       50
CCc| CCUCCU   -     A    CCGU              GAGUUG       CgUG
   GG      GCA GUGCC CGCU    GUAUUUGACAAGCU      GACACUC    \
   CC      CGU uACGG GUGA    CAUAAACUGUUUGA      CUGUGAG    U
GA-^ AUU---   A     C    ACCC              ------       AUGG
.           100        90        80              70
```

mml-miR-224(ψ)

GGGCTTTCAAGTCACTAGTGGTTCCGTTTAGTAGATGATTGTGCATTGTTTCAAAATGGT
GCCCTAGTGACTACAAAGCCC

```
        10        20        30           40
       CA         U   U      AGUA--|   AU
GGGCUUU  AGUCACUAG GGU CCGUUU      GAUG  U
CCCGAAA  UCAGUGAUC CCG GGUAAA      UUAC  G
       CA         -   U      ACUUUG^   GU
80        70         60        50
```

mml-miR-296

AGGACCCTTCCAGAGGGCCCCCCCTCAATCCTGTTGTGCCTAATTCAGAGGGTTGGGTGG
AGGCTCTCCTGAAGGGCTCT

```
        10        20        30        40
| GA      CA       C  C         G   UGCC
AG  CCCUUC  GAGGGCC CC CUCAAUCCU UUG    \
UC  GGGAAG  CUCUCGG GG GGGUUGGGA GAC    U
^ UC      UC       A  U         -   UUAA
         70        60        50
```

mml-miR-297

TGTATGTATGTGTGCATGTGCATaTAtgTGTGTgTATAtATATATATGTA
TTATGTACTCATATATCA

```
        10          20        30
UGU       U      -|              U
   AUGUAUG GUGCAU GUGCAUaUAugUGUG g
   UAUAUAC CAUGUA UAUGUAUAUAUAuAU U
AC-       U      U^              A
        60        50        40
```

mml-miR-298

TCAGGTCTTCAGCAGAAGCcGGGtGGTTCTCCCAGTGGTTTTCCTTGACT

GTGAGGAACTAGCCTGCTGtTTTGCTCAGGAaTGAGCT

```
           10          20         30        40
---   GG    C      --    c  G     -|  C    GGUUU
   UCA  UCUU AGCAGA  AGC GG uGGUU CUC CAGU     \
   AGU  AGGA UCGUUU  UCG CC AUCAA GAG GUCA     U
UCG   a-    C      uG   U  G     G^  U    GUUCC
        80        70        60        50
```

mml-miR-299-5p

AAGAAATGGTTTACCGTCCCACATACATTTTCAATATGTATGTGGGAcGGTAAACCGCTT
CTT

```
        10        20        30
|    A                      UUU
AAGAA UGGUUUACCGUCCCACAUACAU   \
UUCUU GCCAAAUGGcAGGGUGUAUGUA   C
^    C                      UAA
  60        50        40
```

mml-miR-299-3p

```
        10        20        30
|    A                      UUU
AAGAA UGGUUUACCGUCCCACAUACAU   \
UUCUU GCCAAAUGGcAGGGUGUAUGUA   C
^    C                      UAA
  60        50        40
```

mml-miR-301a

ACTGCTAACGAATGCTCTGACTTTATTGCACTACTGTACTTTACAGCTAGCAGTGCAATA
GTATTGTCAAAGCATCTGAAAGCAGG

```
        10        20           30
A     AA  A     C    U---          A----|    C
 CUGCU  CG AUGCU UGAC    UUAUUGCACU     CUGUA \
 GACGA  GU UACGA ACUG    GAUAACGUGA     GACAU U
G     AA  C     A    UUAU          CGAUC^    U
     80        70        60        50
```

mml-miR-301b

CCGCAGGTGCTCTGACGAGGTTGCACTACTGTGCTCTGAGAAGCAGTGCA
ATGATATTGTCAAAGCATCTGGGACCA

```
            10           20        30
----  G        C      G---|       A  GUG  C
    CC CAGGUGCU UGACGA    GUUGCACU CU   CU \
    GG GUCUACGA ACUGUU    UAACGUGA GA   GA U
ACCA  -        A      AUAG^      C  A--  G
       70        60        50          40
```

mml-miR-302a*

CCACCACTTAAACGTGGATGTACTTGCTTTGAAACTAAAGAAGTAAGTGCTTCCATGTTT
TGGTGATGG

```
        10        20        30
|  C    U         U           GAAA
CCA CACU AAACGUGGA GUACUUGCUUU    \
GGU GUGG UUUGUACCU CGUGAAUGAAG    C
^  A    U         U           AAAU
        60        50        40
```

mml-miR-302a

```
        10        20        30
```

```
|  C    U         U           GAAA
CCA CACU AAACGUGGA GUACUUGCUUU    \
GGU GUGG UUUGUACCU CGUGAAUGAAG    C
^  A    U         U           AAAU
        60        50        40
```

mml-miR-302b*

GCTCCCTTCAACTTTAACATGGAAGTGCTTTCTGTGACTTTAAAAtaaGTAAGTGCTTCC
ATGTTTTAGTAGGAGT

```
        10        20        30        40
|    CUUCA   UU          UG   U  GUGACU
GCUCC     ACU  AACAUGGAAG  CUU CU      U
UGAGG     UGA  UUGUACCUUC  GAA Ga      U
^    A----   UU          GU   U  auAAAA
         70        60        50
```

mml-miR-302b

```
        10        20        30        40
|    CUUCA   UU          UG   U  GUGACU
GCUCC     ACU  AACAUGGAAG  CUU CU      U
UGAGG     UGA  UUGUACCUUC  GAA Ga      U
^    A----   UU          GU   U  auAAAA
         70        60        50
```

mml-miR-302c*

CCTTTGCTTTAACATGGGGGTACCTGCTGTGTGAAACAAAAGTAAGTGCTTCCATGTTTC
AGTGGAGG

```
        10        20         30
    UG  UU             C    G-| UG
CCUU  CU  AACAUGGGGGUAC UGCU  UG  \
GGAG  GA  UUGUACCUUCGUG AUGA  AC  A
    GU  CU             A    AA^ AA
       60        50        40
```

mml-miR-302c

```
        10        20         30
    UG  UU             C    G-| UG
CCUU  CU  AACAUGGGGGUAC UGCU  UG  \
GGAG  GA  UUGUACCUUCGUG AUGA  AC  A
    GU  CU             A    AA^ AA
       60        50        40
```

mml-miR-302d

CCTCTACTTTAACATGGAGGCACTTGCTGTGgTATGACAAAAATAAGTGCTTCCATGTTT
GAGTGTGG

```
        10        20         30
| UC     U                CUG-  gU
CC  UACUU AACAUGGAGGCACUUG    UG  A
GG  GUGAG UUGUACCUUCGUGAAU    AC  U
^ U-     U                AAAA  AG
        60        50        40
```

mml-miR-320

GCTTCGCTCCCCTCCGCCTTCTCTTCCCGGTTCTTCCCGGAGTCGGGAAAAGCTGGGTTG
AGAGGGCGAAAAAGGATGAGG

```
        10         20        30         40
G|    C   CCUC-         UU       C      G
 CUUCG UCC     CGCCUUCUC  CCCGGUU UUCCCG A
```

```
 GGAGU AGG     GCGGGAGAG  GGGUCGA AAGGGC G
-^    -   AAAAA         UU       A      U
 80         70        60        50
```

mml-miR-323

TTGGTACTTGGAGAGAGGTGGTCCGTGGCGCGTTCGCTTTATTTATGGCGCACATTACAC
GGTCGACCTCTTTGCAGTATCTAATC

```
           10         20        30         40
UU---|     U G         -  U     GCGC   U    UUA
     GGUACU G AGAGAGGU GG CCGUG    GU CGCU   U
     CUAUGA C UUUCUCCA CU GGCAC    CA GCGG   U
CUAAU^     - G        G  -     AUUA   C    UAU
     80          70          60         50
```

mml-miR-324-5p

CTGACTATGCCTCCCCGCATCCCCTAGGGCATTGGTGTAAAGCTGGAGACCCACTGCC
CCAGGTGCTGCTGGGGGTTGTAGTC

```
        10        20        30        40
CU|     GC     C   UCC   A     U   U  AAAG
  GACUAU  CUCCC GCA   CCU GGGCA UGG GU    C
  CUGAUG  GGGGG CGU   GGA CCCGU ACC CA    U
--^     UU     U   CGU   C     C   -  GAGG
    80        70        60         50
```

mml-miR-324-3p

```
       10        20        30        40
CU|     GC     C   UCC   A     U   U  AAAG
  GACUAU  CUCCC GCA   CCU GGGCA UGG GU    C
  CUGAUG  GGGGG CGU   GGA CCCGU ACC CA    U
--^     UU     U   CGU   C     C   -  GAGG
    80        70        60         50
```

mml-miR-325

ATgCAGTGCTTGGTTCCTAGTAGGTGTCCAGTAAGTGTTTGTtACATAATTTGTTTATTG
AGGACCTCCTATCAATCAAGCACTGTGCTAGGCTCTGG

```
                  10        20          30                40
AU----------             CC   U    G  -      -------|   UU
            GCAGUGCUUGGUU  UAG AGGU UC CAGUAA       GUGU  \
            UGUCACGAACUAA  AUC UCCA GG GUUAUU       UACA  G
GGUCUCGGAUCG             CU   C    -  A      UGUUUAA^   uU
       90        80        70         60        50
```

mml-miR-329-1

GTGGTACCTGAAGgGAGGTTTTCTGGGTTTCTGTTTCTTTAATGAGGAtGAAACACACCT
GGTTAACCTCTTTTCCAGTATCAA

```
        10        20        30        40
G|     CU            UU       UUC      U  AA
 UGGUAC  GAAGgGAGGUU  CUGGGU   UGUUUC UU  U
 ACUAUG  CUUUUCUCCAA  GGUCCA   ACAAAG AG  G
A^     AC            UU       C--      u  GA
   80        70        60           50
```

mml-miR-329-2

GGTACCTGAAGgGAGGTTTTCTGGGTcTCTGTTTCTTTAcTGAGGAtGAAACACACCTGG
TTAACCTCTTTTCCAGTATC

```
        10        20        30        40
|    CU            UU UG   cUC       U  Ac
GGUAC  GAAGgGAGGUU  C  GGU   UGUUUC UU  U
```

```
CUAUG  CUUUUCUCCAA  G  CCA   ACAAAG AG  G
^    AC           UU GU   C--      u  GA
.        70        60          50
```

mml-miR-330

CTTTGGCGATCACTGCCTCTCTGGGCCTGTGTCTTAGGCTCTGCAAGATCAACCGAGCAA
AGCACACGGCCTGCAGAGAGGCAGCGCTCTGCCC

```
         10        20           30        40
CUUU   -  UCA           --     -|   U   AG    UGCAAG
    GGC GA   CUGCCUCUCUG  GGCC UGUG CUU  GCUC      \
    CCG CU   GACGGAGAGAC  CCGG ACAC GAA  CGAG      A
C---   U  CGC           GU     C^   -   A-    CCAACU
      90        80        70          60          50
```

mml-miR-331

GAGTTTGGTTTTGTTTGGGTTTGTTCTAGGTATGGTCCCAGGGATCCCAGATCAAACCAG
GCCCCTGGGCCTATCCTAGAACCAACCTAAaCTC

```
        10        20        30        40        50
GAGUUUGGUUUU|        U        U    U       AU CCAGAU
            GUUUGGGUU GUUCUAGG AUGG CCCAGGG  C      \
            CaAAUCCAA CAAGAUCC UAUC GGGUCCC  G      C
CU----------^        C        -    C       CG ACCAAA
             90        80         70        60
```

mml-miR-335

GAGCGGGGGTCAAGAGCAATAACGAAAAATGTTTGTCATAAACCGTTTTTCATT
ATTGCTCCTGACCTCCTCTCATTTGCTATATTCA

```
                      10        20        30
--------------|  C        A         C        U  GU
              GAG GGGGGUCA GAGCAAUAA GAAAAAUG UU  C
              CUC CCUCCAGU CUCGUUAUU CUUUUUGC AA  A
ACUUAUAUCGUUUA^  U        C         A        C  AU
       80        70        60        50        40
```

mml-miR-337

GTAGTCAGTAGTTGGGGGGTGGGAACGGCTTCATACAGGAGTTGATGCACAGTTATCCAG
CTCCTATATGATGCCTTTCTTCATCCCCTTCAA

```
        10        20          30        40          50
GUAGUCAGUAGUU        -    C   U     C       -|   CA
             GGGGGGUG GGAA GGC UCAUA AGGAGUU GAUG  C
             UCCCCUAC UCUU CCG AGUAU UCCUCGA CUAU  A
AACU---------        U    U   U     A       C^   UG
  90                 80        70        60
```

mml-miR-338

TCTCCAACAATATCCTGGTGCTGAGTGATGACTCAGGtGACTCCAGCATC
AGTGATTTTGTTGAAGA

```
        10         20         30
   C      U   -        -|   G  GAC
UCU CAACAA AUC CUGGUGCU GAGU AU   \
AGA GUUGUU UAG GACUACGA CUCA uG   U
   A      U   U        C^   G  GAC
      60        50        40
```

mml-miR-339

CGGGGCGGCCGCTCTCCCTGTCCTCCAGGAGCTCACGTGTGCCTGCCTGT
GAGCGCCTCGACGACAGAGCCGGCGCCcGCCCCAGTGTCTGCGC

```
                  10        20        30        40
```

```
C----------|      C    CUCC     C  C   A       U  G
           GGGGCGG CGCU    CUGUC UC AGG GCUCACG GU C
           CCCCGcC GCGG    GACAG AG UCC CGAGUGU CG C
CGCGUCUGUGA^      C    CCGA     C  C   G       C  U
   90        80        70        60        50
```

mml-miR-340

TTGTACCTGGTGTGATTATAAAGCAATGAGACTGATTGTCATATGTtGTTTGTGGGATCC
GTCTCAGTTACTTTATAGCCATACCTGGTATCTTA

```
          10        20         30        40
UU--     U      AU       CAA-|     U    G     UGU
    GUACC GGUGUG  UAUAAAG    UGAGAC GAUU UCAUA   \
    UAUGG CCAUAC  AUAUUUC    ACUCUG CUAG GGUGU   u
AUUC     U      CG       AUUG^     C    -     UUG
    90        80        70        60         50
```

mml-miR-342

GAAACTGGGCTCAAGGTGAGGGGTGCTATCTGTGATTGAGGGACATGGTTAATGGAATTG
TCTCACACAGAAATCGCACCCGTCACCTTGGCCTACTTA

```
        10        20           30        40       50
GAAAC     U        G       UA--|     AUUGA      UGG  A
     UGGGC CAAGGUGA GGGUGC    UCUGUG     GGGACA   UU A
     AUCCG GUUCCACU CCCACG    AGACAC     CUCUGU   AG U
AUUC-     -        G       CUAA^     A----      UA-  G
          90        80        70            60
```

mml-miR-345

AAACCCTAGGTCgGCTGACTCCTAGTCaAGGGCTCGTGgTGGCTGGTGGG
CCCTGAACGAGGGtTCTGGAGGCCTGGGTTTGAATATC

```
               10        20        30        40
-------|     U     gGCU  C    A  Ca        UGgU
       AAACCC AGGUC    GA UCCU GU  AGGGCUCG    G
       UUUGGG UCCGG    CU GGGA CA  UCCCGGGU    G
CUAUAAG^     -     AGGU  u    G  AG        GGUC
       80         70        60        50
```

mml-miR-346

GTCTGTCTGCCCGCATGCCTGCCTCTCTGTTGCTCTGAAGGAGGCAGGGGCTGGGCCTGC
AGCTGCCTGGGCAGAGCGGCTCCTGC

```
          10        20         30        40
  |    G UG     U     CU     -  AUG       U  GUUG
--UCUGU U  GGCG CUGU  GCCC GC  CCUGCCUC CU     C
  AGACG G  CCGU GACG  CGGG CG  GGACGGAG GA     U
\ ^    - GU     C     UC     U  G--       -  AGUC
         80        70          60         50
```

mml-miR-361

GGAGCTTATCAGAATCTCCAGGGGTACTTTATAATTTCAAAAAGTCCCCCAGGTGTGATT
CTGATTTGCTTC

```
        10          20        30
     UU         U--| A    U     AUAA
GGAGC  AUCAGAAUC   CC GGGG ACUUU    U
CUUCG  UAGUCUUAG   GG CCCC UGAAA    U
     UU         UGU^ A    C     AACU
 70        60        50        40
```

mml-miR-362

CTcGAATCCTTGGAACCTAGGTGTGAGTGCTATTTCAGTGCAACACACCTATTCAAGGAT
TCAAA

```
        10         20         30
CUc|          ACC        A- UG   AU
   GAAUCCUUGGA   UAGGUGUG   G   CU   \
   CUUAGGAACUU   AUCCACAC   C   GA   U
AAA^          ---        AA GU   CU
    60            50        40
```

mml-miR-363*

TGTTGTCGGGTGGATCACGATGCAATTTTGATtAGTATCATAGGAGAAAAATTGCACGGT
ATCCATCTGTAAACC

```
        10         20         30
U   GU           CA  A          GA--|  GU
 GUU  CGGGUGGAU  CG UGCAAUUUU      UuA  A
 CAA  GUCUACCUA  GC ACGUUAAAA      GAU  U
C   AU           UG  -          AGAG^  AC
    70         60         50         40
```

mml-miR-363

```
        10         20         30
U   GU           CA  A          GA--|  GU
 GUU  CGGGUGGAU  CG UGCAAUUUU      UuA  A
 CAA  GUCUACCUA  GC ACGUUAAAA      GAU  U
C   AU           UG  -          AGAG^  AC
    70         60         50         40
```

mml-miR-365-1

ACCGCAGGGAAAATGAGGGACTTTTGGGGGCAGATGTGTTTCCATTCCACTATCATAATG
CCCCTAAAAATCCTTATTGCTCTTGCA

```
        10         20         30              40
ACC      AA        AC          ----   -|  UUUC
   GCAGGG  AAUGAGGG  UUUUGGGGGCA    GAU GUG    \
   CGUUCU  UUAUUCCU  AAAAUCCCCGU    CUA CAC    C
A--      CG        A-          AAUA   U^  CUUA
                 80        70         60         50
```

mml-miR-365-2

AGAGTGTTCAAGGACAGCAAGAAAAATGAGGGACTTTCAGGGGCAGCTGTGTTTTCTGAC
TCAGTCATAATGCCCCTAAAAATCCTTATTGTTCTTGCAGTGTGCATCaGG

```
          10         20         30         40             50
A--  G   UCAAGG| A       A          AC   C       -----    U   U
   GA UGU       AC GCAAGAA AAUGAGGG  UUU AGGGGCA     GCUG GUU U
   CU ACG       UG CGUUCUU UUAUUCCU  AAA UCCCCGU     UGAC CAG C
GGa  -   UG----^ A       G          AA   -       AAUAC    U   U
 .              100        90         80          70         60
```

mml-miR-367

CCAcTACTGTTGCTAATATGCAACTCTGTTGAAcAcAAATTGGAATTGCACTTTAGCAAT
GGTGATGG

```
        10         20          30
   c            UA     C    --|  A
CCA UACUGUUGCUAA  UGCAA UCUG   UUG A
GGU GUGGUAACGAUU  ACGUU AGGU   AAc c
   A            UC     A    UA^  A
       60         50         40
```

mml-miR-369-5p

TTGAAGGGAGATCGACCGTGTTATATTCGCTTTATTGACTTCGAATAATACATGGTTGAT
CTTTTCTCAG

```
        10        20        30
U|  AG              UA      CUUUA
 UGA  GGAGAUCGACCGUGU  UAUUCG     U
 ACU  UUUCUAGUUGGUACA  AUAAGC     U
G^  CU              UA      UUCAG
.        60        50        40
```

mml-miR-369-3p

```
        10        20        30
U|  AG              UA      CUUUA
 UGA  GGAGAUCGACCGUGU  UAUUCG     U
 ACU  UUUCUAGUUGGUACA  AUAAGC     U
G^  CU              UA      UUCAG
.        60        50        40
```

mml-miR-370

AGACAGAGAAGCCAGGTCACGTCTCTGCAGTTACACAGCTCAtGAGTGCCTGCTGGGGTG
GAACCTGGTCTGTCT

```
        10        20        30
|     AGAA       CA GU   U    U   A  GC
AGACAG    GCCAGGU  C  CUC GCAG UAC CA  \
UCUGUC    UGGUCCA  G  GGG CGUC GUG Gu  U
^     ----       AG UG   U    C   A  AC
    70            60        50
```

mml-miR-371

GTGGCACTCAAACTGTGGGGGCACTTTCTGCTCTCTGGTGAAAaaaGTGCCGCCATgTTT
TGAGTGTTAC

```
        10        20          30
            CU    G        C--|   C
GUGGCACUCAAA  GUGG GGCACUUU   UGCU U
CAUUGUGAGUUU  UACC CCGUGaaa   GUGG C
            Ug    G        AAA^   U
.        60        50        40
```

mml-miR-372

GTGatCCTCAAATGTGGAGCACTATTCTGATGTCCAAGTGGAAAGTGCTGCGACATTTGA
GCGTCAC

```
        10         20        30
     C         -|G      A    GAUGU
GUGaU CUCAAAUGU G AGCACU UUCU     \
CACUG GAGUUUACA C UCGUGA AAGG     C
     C         G^G      -    UGAAC
      60        50         40
```

mml-miR-373*

GGGATACcCAAAATGGGaGCaCTTTCCCTTTTGTCTGTgCTGGGAAGTGCTTCGATTTTG
GGGTGTCCC

```
         10        20        30
      -|       G         U   UUUUGU
GGGAUA CcCAAAAU GGaGCaCUU CCC      \
CCCUGU GGGUUUUA CUUCGUGAA GGG      C
      G^       G         -   UCgUGU
        60        50         40
```

mml-miR-373

```
         10        20        30
      -|       G         U   UUUUGU
GGGAUA CcCAAAAU GGaGCaCUU CCC      \
CCCUGU GGGUUUUA CUUCGUGAA GGG      C
      G^       G         -   UCgUGU
        60        50         40
```

mml-miR-374a

TACATCGGCCATTATAATACAACCTGATAAGTGTTAcAGCACTTATCAGATTGTATTGTAATTGTCTGTGT
A

```
        10        20        30
|    C   C           C           U
UACAU GGC AUUAUAAUACAA CUGAUAAGUGU A
AUGUG CUG UAAUGUUAUGUU GACUAUUCACG c
^    U   U           A           A
 70        60        50        40
```

mml-miR-374b

ACTCGGATGGATATAATACAACCTGCTAAGTGTCCTAGCACTTAGCAGGT
TGTATTATCATTGTCCGTGTCT

```
          10        20        30
--| U       AU                   C
  AC CGGAUGG  AUAAUACAACCUGCUAAGUGU C
  UG GCCUGUU  UAUUAUGUUGGACGAUUCACG U
UC^ U       AC                   A
 70        60        50        40
```

mml-miR-374b*

```
         10        20        30
--| U       AU                   C
  AC CGGAUGG  AUAAUACAACCUGCUAAGUGU C
  UG GCCUGUU  UAUUAUGUUGGACGAUUCACG U
UC^ U       AC                   A
 70        60        50        40
```

mml-miR-375

CCCCGCGACGAGCCCCTCGCACAAACCGGACCTGAGCGTTTTGTTCGTTCGGCTCGCGTGAGGC

```
         10        20        30
C  -|   A      CCU  C     C  GAC
 CC CGCG CGAGCC   CG ACAAA CG   C
 GG GUGC GCUCGG   GC UGUUU GC   U
C  A^   -      CUU  U     U  GAG
   60          50        40
```

mml-miR-376a-1*

TAAAAGGTAGATTCTCCTTCTATGAGTACATTATTTATGATTAATCATAGAGGAAAATCCACGTTTTC

```
        10        20         30
U    G  A    C   U       GUA--|  UA
 AAAA GU GAUU UCC UCUAUGA     CAU  \
 UUUU CA CUAA AGG AGAUACU     GUA  U
C    G  C    A   -       AAUUA^  UU
       60         50        40
```

mml-miR-376a-1

```
        10        20         30
U    G  A    C   U       GUA--|  UA
 AAAA GU GAUU UCC UCUAUGA     CAU  \
```

```
 UUUU CA CUAA AGG AGAUACU     GUA  U
C    G  C    A   -       AAUUA^  UU
       60         50         40
```

mml-miR-376a-2

GGTATTTAAAAGGTAGATTTTCCTTCTATGGTTACGTGTTTGATGGTTAA
TCATAGAGGAAAATCCACGTTTTCAGTATC

```
        10        20        30
      U    G  A        U          --|  GU
GGUAUU AAAA GU GAUUUUCC UCUAUGGUUA  CGU  \
CUAUGA UUUU CA CUAAAAGG AGAUACUAAU  GUA  U
      C    G  C        -          UG^  GU
.        70        60          50
```

mml-miR-376b

CAGTCCTTCTTTGGTATTTAAAACGTGGATATTCCTTCTATGTTTACGTG
ATTCCTGGTTAATCATAGAGGAAAATCCATGTTTTCAGTATCAAATGCTG

```
        10        20        30        40        50
|   CCUUC         U           A     U     UUUACG    UC
CAGU     UUUGGUAUU AAAACGUGGAU UUCCU CUAUG      UGAU  \
GUCG     AAACUAUGA UUUUGUACCUA AAGGA GAUAC      AUUG  C
^   U----         C           A     -     UA----    GU
.            90        80        70              60
```

mml-miR-376c

AAAAGGTGGATATTCCTTCTATGTTTATGTTATTTATGGTTAAACATAGAGGAAATTCCACGTTTT

```
        10        20        30
|   G     UA      U         UGUUAU
AAAA GUGGA  UUCCU CUAUGUUUA      \
UUUU CACCU  AAGGA GAUACAAAU      U
^   G     UA      -         UGGUAU
     60        50          40
```

mml-miR-377

TTGAGCAGAGGTTGCCCTTGGTGAATTCGCTTTATTTATGTTGAATCACACAAAGGCAACTTTTGTTTG

```
        10        20          30
UU              C   -|   A    - UUUA
  GAGCAGAGGUUGCC UUG GUGA UUCG C    \
  UUGUUUUCAACGG AAC CACU AAGU G    U
G-              A   A^   -    U UAUU
         60        50          40
```

mml-miR-378

AGGGCTCCTGACTCCAGGTCCTGTGTGTTACCTcGAAATAGCACTGGACTTGGAGTCAGAAGGCCT

```
        10        20        30
|  G  C              UGU      CCU
AGG CU CUGACUCCAGGUCC   GUGUUA   c
UCC GA GACUGAGGUUCAGG   CACGAU   G
^  G  A              U--      AAA
     60        50          40
```

mml-miR-379

AGAGATGGTAGACTATGGAACGTAGGCGTTATGATTTtTGACCTATGTAACATGGTCCACTAACTCT

```
        10        20         30
    A    A       GA       -| UUAU
AGAG UGGU GACUAUG  ACGUAGG CG    G
UCUC AUCA CUGGUAC  UGUAUCC GU    A
```

```
    A    C       AA       A^ uUUU
      60        50        40
```

mml-miR-380-5p

AAGATGGTTGACCATAGAACATGCGCTATCTCTGTGTCGTATGTAATATGGTCCACgTCTT

```
        10        20        30
|     GUU       GA       C   C
AAGAUG   GACCAUA  ACAUGCG UAU U
UUCUgC   CUGGUAU  UGUAUGC GUG C
^     AC-       AA       U   U
60         50         40
```

mml-miR-380-3p

```
        10        20        30
|     GUU       GA       C   C
AAGAUG   GACCAUA  ACAUGCG UAU U
UUCUgC   CUGGUAU  UGUAUGC GUG C
^     AC-       AA       U   U
60         50         40
```

mml-miR-381

TACTTAAAGCGAGGTTGCCCTTTGTATATTCGGTTTATTGACATGGAATATACAAGGGCA
AGCTCTCTGTGAGTA

```
        10        20        30
|     A  C   G        U        G  UUA
UACUUA AG GAG UUGCCCUU GUAUAUUC GU   U
AUGAGU UC CUC AACGGGAA CAUAUAAG UA   U
^     G  U   G        -        G  CAG
    70        60         50        40
```

mml-miR-382

TACTTGAAGAGAAGTTGTTCGTGGTGGATTCGCTTTACTTATGACGAATCATTCACGGAC
AACACTTTTTTCAGTA

```
        10         20        30
|   U      A-           UG       C  UAC
UACU GAAGAGA  GUUGUUCGUGG  GAUUCG UU   \
AUGA CUUUUUU  CAACAGGCACU  CUAAGC AG   U
^   -      CA           UA       -  UAU
      70        60        50         40
```

mml-miR-383

CTCCTCAGATCAGAAGGTGATTGTGGCTTTGGGTGGATATTAATCAGCCACAGCACTGCC
TGGTCAGAAAGAG

```
         10        20        30
   C-| A      AA    A        UUG   GGA
CUC  UC GAUCAG  GGUG UUGUGGCU   GGU   U
GAG  AG CUGGUC  UCAC GACACCGA   CUA   A
   AA^ A      CG    -        ---   AUU
  70        60         50           40
```

mml-miR-384

TGTTAAATtAGGAATTgTAAACAATTCCTAGgCAATATGTATAATGTTCATAAGaCATTC
CTAGAAATTGTTCATAATGCCTGTAACA

```
        10        20        30        40        50
U    AAU|   A     A       C     CAAUAUGUAU      CA
 GUUA   UAGG AUUgU AACAAUU CUAGg          AAUGUU  \
 CAAU   GUCC UAAUA UUGUUAA GAUCC          UUACaG  U
A    ---^   G     C       A     ----------      AA
          80        70        60
```

mml-miR-409-5P

TGGTACTCGGGGAGAGGTTACCCGAGCAACTTTGCATCTGGACGACGAATGTTGCTCGGT
GAACCCCTTTTCGGTATCA

```
        10        20        30        40
|     U       A    AC         -    CA  UG
UGGUAC CGGGGAG GGUU  CCGAGCAAC UUUG  UC  \
ACUAUG GCUUUUC CCAA  GGCUCGUUG AAGC  AG  G
^     -       C    GU         U    --  CA
         70        60        50
```

mml-miR-409-3p

```
        10        20        30        40
|     U       A    AC         -    CA  UG
UGGUAC CGGGGAG GGUU  CCGAGCAAC UUUG  UC  \
ACUAUG GCUUUUC CCAA  GGCUCGUUG AAGC  AG  G
^     -       C    GU         U    --  CA
         70        60        50
```

mml-miR-410

GGTACCTGAGgAGAGGTTGTCTGTGATGAGTTCGCTTTTATTAATGACGAATATAACACA
GATGGCCTGTTTTCAGTACC

```
        10        20        30        40
|    C      G             A  A     CUUUUA
GGUAC UGAGgA AGGUUGUCUGUG UG GUUCG      \
CCAUG ACUUUU UCCGGUAGACAC AU UAAGC      U
^    -      G             A  A     AGUAAU
.         70        60        50
```

mml-miR-411

TGGTACTTGGAGAGATAGTAGACCGTATAGCGTACGCTTTATCTGTGACGTATGTAACAC
GGTCCACTAACCCTCAGTATCA

```
        10        20        30        40
U|      UG   AGA    A      AUA       C  UAU
 GGUACU   GAG   UAGU GACCGU   GCGUACG UU   \
 CUAUGA   CUC   AUCA CUGGCA   UGUAUGC AG   C
A^     --   CCA    C       CAA       -  UGU
 80          70        60        50
```

mml-miR-412

CTGGGGTACGGGGATGGATGGTCGACCAGTTGGAAAGTAATTGTTTCTAATGTACTTCAC
CTGGTCCACTAGCCGTCCGTATCCGCTGCAG

```
            10        20        30        40
CUG----|        GA   A    C      U   AA    AUUGUU
       GGGUACGGG  UGG UGGU GACCAG UGG  AGUA      \
       CCUAUGCCU  GCC AUCA CUGGUC ACU  UCAU      U
GACGUCG^        --   G    C      C   --    GUAAUC
90        80           70        60           50
```

mml-miR-421

CACATTGTAGGCCTCATTAAATGTTTGTTGAATGAAAAAATGAATCATCAACAGACATTA
ATTGGGCGCCTGCTCTGTG

```
        10        20         30
    UU      -|   UUA            A   AAAA
CACA  GUAGGC CUCA   AAUGUUUGUUGA UGA    \
GUGU  CGUCCG GGGU   UUACAGACAACU ACU    A
    CU      C^   UAA            -   AAGU
        70        60         50
```

mml-miR-422a

GAGAGAAGCACTGGACTcAGGGTCAGAAGGCCTGAGTCTCcCTGCTGCAGATGGGCTgTg
TGTCCCTGAGCCAAGCcTTGTCCTCCCTGG

```
              10        20        30        40
-----  -| G   CAC   A       U  GAA     GA    CcCU
     GA GA AAG   UGG CUcAGGG CA   GGCCU  GUCU    \
     CU CU UUc   ACC GAGUCCC gU   UCGGG  UAGA    G
GGUCC  C^ G   CGA   -       U  gUG     --    CGUC
.        80         70        60          50
```

mml-miR-423

ATAAAGGAAGTTAGGCTGAGGGGCAGAGAGCGAGACTTTTCTATTTTCCAAAAGCTCGGT
CTGAGGCCCCTCAGTCTTGCTTCCTACCCCGCGC

```
             10        20         30        40
AUAA-----       U            AG-|  G    A     CUAU
         AGGAAGU AGGCUGAGGGGC   AGA CGAG CUUUU    \
         UCCUUCG UCUGACUCCCCG   UCU GCUC GAAAA    U
CGCGCCCCA       U            GAG^  G    -     CCUU
   90        80        70        60         50
```

mml-miR-424

CGAGGGGATACAGCAGCAATTCATGTTTTGAAGTGTTCTAAATGGTTCAAAACGTGAGGC
GCTGCTATACCCCCTCGTGGGGAAGGTAGAAGGTGGGG

```
                             10        20        30
---------------------|      A  C      AA             G    C
                     CGAGGGG UA AGCAGC  UUCAUGUUUUGAA UGUU \
                     GCUCCCC AU UCGUCG  GAGUGCAAAACUU GUAA U
GGGGUGGAAGAUGGAAGGGGU^      C  A      CG             G    A
       90        80        70        60        50        40
```

mml-miR-425-5p

GAAAGCGCTTTGGAATGACACGATCACTCCCGTTGAGTGGGCcCCCGAGAAGCCATCGGG
AATGTCGTGTCCGCCCAGTGCTCTTTC

```
        10        20        30             40
     C  U    AAU       U  C     U  A| -----  G
GAAAG GC UUGG   GACACGA CA UCCCG UG GU     GG C
CUUUC CG GACC   CUGUGCU GU AGGGC AC CG     CC c
     U  U    CGC       -  A     U  -^ AAGAG  C
      80        70         60         50
```

mml-miR-425-3p

```
        10        20        30             40
     C  U    AAU       U  C     U  A| -----  G
GAAAG GC UUGG   GACACGA CA UCCCG UG GU     GG C
CUUUC CG GACC   CUGUGCU GU AGGGC AC CG     CC c
     U  U    CGC       -  A     U  -^ AAGAG  C
      80        70         60         50
```

mml-miR-429

CGCCGGCCGATGAGCGTCTTACCAGACAcGGTTAGACCTGGCtCTCTGTCTAATACTGTC
TGGTAAAACCGTCCATCCGCgGC

```
        10         20        30        40
C|   G C    AG  UC-          cG       CUGG
 GCCG C GAUG  CG   UUACCAGACA  GUUAGAC    C
 CGgC G CUAC  GC   AAUGGUCUGU  UAAUCUG    u
-^   - C    CU  CAA          CA       UCUC
   80         70        60        50
```

mml-miR-431

TCCTGCTTGTCCTGCGAGGTGTCTTGCAGGCCGTCATGCAGGCCACACTGACGGTAACGT
TGCAGGTCGTCTTGCAGGGCTTCTCGCAAGACGACATCCTCATCACCAACGACG

```
                       10        20        30        40
UCC---------------  C     C       U          C  UCA     ----|  ACA
                  UG UUGUC UGCGAGG GUCUUGCAGG CG   UGCAG    GCC   C
                  AC AGCAG ACGCUCU CGGGACGUUC GC   ACGUU    UGG   U
GCAGCAACCACUACUCCU  -     A       U          U  UGG     GCAA^  CAG
  110       100         90        80        70        60        50
```

mml-miR-432

TGACTCCTCCATgTCTTGGAGTAGGTCATTGGGTGGATCCTCTATTTCCTTAtGTGGGCC
ACTGGATGGCTCCTCCATGTCTTGGAGTAGATCA

```
         10        20        30        40
U  -| C     UgUCU     UA       G     A   UCUAUUU
 GA CU CUCCA     UGGAG  GGUCAUU GGUGG UCC       \
 CU GA GAGGU     ACCUC  UCGGUAG UCACC GGG       C
A  A^ U     UCUGU     C-       G     -   UGuAUUC
   90        80         70        60         50
```

mml-miR-432*

```
         10        20        30        40
U  -| C     UgUCU     UA       G     A   UCUAUUU
 GA CU CUCCA     UGGAG  GGUCAUU GGUGG UCC       \
 CU GA GAGGU     ACCUC  UCGGUAG UCACC GGG       C
A  A^ U     UCUGU     C-       G     -   UGuAUUC
   90        80         70        60         50
```

mml-miR-433

CCaGGGAGAAGTACGGTGAGCCTGTCATTATTCAGAGAGGCTAGATCCTC
TGTGTTGAGAAGGATCATGATGGGCTCCTCGGTGTTCTCCAGG

```
        10        20        30                40
  aG      GUA   U           U   --------| G     CU
CC  GGAGAA   CGG GAGCCUGUCAU AUU         CA AGAGG  A
GG  CCUCUU   GCU CUCGGGUAGUA UAG         GU UCUCC  G
  A-      GUG   C           C   GAAGAGUU^ G     UA
   90        80        70        60        50
```

mml-miR-448

GCCGGGAGGTTGAACATCCTGCATAGTGCTGCCAGGAAATCCCTATTTCATActAagaGG
GGCTGGCTGGTTGCATATGTAGGATGTCCCATCTCCCAGCCtACTTCGTCA

```
                   10        20        30        40        50
-----------| C       UGA            G   U     GAAA     A  UCA
           GC GGGAGGU   ACAUCCUGCAUA UGC GCCAG     UCCCU UU   \
           CG CCCUCUA   UGUAGGAUGUAU ACG UGGUC     GGGGa aA   U
ACUGCUUCAuC^ A       CCC            -   U     GGUC     g  ucA
         100        90        80         70        60
```

mml-miR-449a

CTGTGTGTGATGAGCTGGCAGTGTATTGTTAGCTGGTTGAATATGTGAATGGCATCaGCT
AACATGCAACTGCTGTCTTATTGCATATACA

```
         10        20         30        40
C-      UG      C      -|    U          UGAA   G
  UGUGUG  AUGAG UGGCAG UGUAU GUUAGCUGGU    UAU U
  AUAUAC  UAUUC GUCGUC ACGUA CAAUCGaCUA    GUA G
AC      GU      U      A^    -          CG--   A
90        80        70         60           50
```

mml-miR-449b

TGACCTGAATCAGGTAGGCAGTGTATTGTTAGCTGGCTGCTTGaGTCAAG
TCAGCAGCCACAACTACCCTGCCACTTGCTTCTGGATAAATTCTTCT

```
                 10        20        30        40
UGA---------| U   U     A     U   UU   AGC        U  GU
            CC GAA CAGGU GGCAG GUA  GUU   UGGCUGCU Ga  C
            GG CUU GUUCA CCGUC CAU  CAA   ACCGACGA CU  A
UCUUCUUAAAUA^ U   C     -     C   --   C--        -  GA
      90        80         70             60         50
```

mml-miR-450a-1

AAAtGATACTAAACTGTTTTTGCGATGTGTTCCTAATATGtACTATAAATATATTGGGAA
CATTTTGCATGTgTAGTTTTGTATCAATATA

```
          10        20        30        40
AAA--|     U      UUUU     U               ACU
     uGAUAC AAACUG    UGCGA GUGUUCCUAAUAUGu   \
     ACUAUG UUUGAU    ACGUU UACAAGGGUUAUAUA   A
AUAUA^     U      gUGU     U               AAU
90         80        70        60        50
```

mml-miR-450a-2

CCAAAGAAAGATGCTAAACTATTTTTGCGATGTGTTCCTAATATGTAATATAAATGTATT
GGGGACATTTTGCATTCATAGTTTTGTATCAATAATATGG

```
         10        20        30        40
CCAAAGAAA-|    U       UUU     U               AAU
          GAUGC AAACUAU   UGCGA GUGUUCCUAAUAUGU   \
          CUAUG UUUGAUA   ACGUU UACAGGGGUUAUGUA   A
GGUAUAAUAA^    U       CUU     U               AAU
.        90        80        70        60
```

mml-miR-450b-5p

GCAGAATTATTTTTGCAATATGTTCCTGAATATGTAgTATAAGcGTATTG
GGATCATTTTGCATCCATAGTTTTGTAT

```
          10        20        30
--|         UUU     U   U    G       AgU
  GCAGAAUUAU   UGCAA AUG UCCU AAUAUGU   \
  UGUUUUGAUA   ACGUU UAC AGGG UUAUGcG   A
UA^         CCU     U   U    -       AAU
       70        60        50         40
```

mml-miR-450b-3p

```
          10        20        30
--|         UUU     U   U    G       AgU
  GCAGAAUUAU   UGCAA AUG UCCU AAUAUGU   \
  UGUUUUGAUA   ACGUU UAC AGGG UUAUGcG   A
UA^         CCU     U   U    -       AAU
       70        60        50         40
```

mml-miR-451

CTTGGGAATGGCAAGGAAACCGTTACCATTACTGAGTTTAGTAATGGTAAgGGTTCTCTT
GCTATAtCCAGA

```
        10        20        30
C|    A        GA    G            A
 UUGGG AUGGCAAG  AACC UUACCAUUACUG G
 GACCu UAUCGUUC  UUGG AAUGGUAAUGAU U
A^    A        UC    g            U
```

```
 70        60        50        40
```

mml-miR-452

GCTAAGCACTTACAACTGTTTGCAGAGGAAACTGAGACTTTGTAACTATGTCTCAGTCTC
ATCTGCAAAGAAGTAAGTGCTTTGC

```
        10        20        30        40
| U          AA  GU       G  A        UUUGU
GC AAGCACUUAC  CU  UUGCAGA GA ACUGAGAC     \
CG UUCGUGAAUG  GA  AACGUCU CU UGACUCUG     A
^ U          AA  --       A  C        UAUCA
    80        70          60        50
```

mml-miR-452*:

```
        10        20        30        40
| U          AA  GU       G  A        UUUGU
GC AAGCACUUAC  CU  UUGCAGA GA ACUGAGAC     \
CG UUCGUGAAUG  GA  AACGUCU CU UGACUCUG     A
^ U          AA  --       A  C        UAUCA
    80        70          60        50
```

mml-miR-453

GCAGGAATGCTGtGAGCAGTGCCACCTCATGGTACTCGGAGGGAGGTTGTCCGTGGTGAG
TTCGCATTATTTAATGATGC

```
            10        20         30
   G----|    C       AG      CU-  UGGUA   GG
GCA     GAAUG UGUGAGC  UGCCAC   CA     CUC  \
CGU     UUUAU ACGCUUG  GUGGUG   GU     GAG  A
   AGUAA^    U       A-      CCU  UG---   GG
.        70        60         50
```

mml-miR-454

TCTGTTTATCACCAGATCCTAGAACCCTATCAATATTGTCTCTGCTGTGT
AAATAGTTCTGAGTAGTGCAATATTGCTTATAGGGTTTTGGTGTTTGGgA
AGAACAATGGGCAGG

```
               10        20        30               40          50
UC-------     A  A      C            ---         ----   U--|    G
         UGUUU UC CCAGAU CUAGAACCCUAU   CAAUAUUGU    CUC   GCUGU U
         ACAAG Ag GGUUUG GGUUUUGGGAUA   GUUAUAACG    GAG   UGAUA A
GGACGGGUA     A  -      U            UUC         UGAU   UCU^    A
   110       100         90        80        70        60
```

mml-miR-454*

```
               10        20        30               40          50
UC-------     A  A      C            ---         ----   U--|    G
         UGUUU UC CCAGAU CUAGAACCCUAU   CAAUAUUGU    CUC   GCUGU U
         ACAAG Ag GGUUUG GGUUUUGGGAUA   GUUAUAACG    GAG   UGAUA A
GGACGGGUA     A  -      U            UUC         UGAU   UCU^    A
   110       100         90        80        70        60
```

mml-miR-455

TCCCTGGCGTGAGGGTATGTGCCTTTGGACTACATCGTGGAAGCCAGCACCATGCAGTCC
ATGGGCATATACACTTGCCTCAAGGCCTATGTCATC

```
             10             20        30        40
UCCCU----   G-     -----|         U      A   C   GAA
         GGC  UGAGG     GUAUGUGCCU UGGACU CAU GUG   G
         CCG  ACUCC     CAUAUACGGG ACCUGA GUA CAC   C
CUACUGUAU   GA     GUUCA^         U      C   C   GAC
```

```
     90        80        70        60        50

mml-miR-484
GTCAGGCTCAGTCCCCTCCCGATAAACCCCTAAATAGGGACTTTCCCGGGGGGTGACCCT
GGC
        10        20         30
|     CUCA   C       AU   C-    A
GUCAGG    GUC CCUCCCG  AAA  CCCU A
CGGUCC    CAG GGGGGGC  UUU  GGGA A
^     ----   U       CC   CA    U
  60            50        40

mml-miR-485-5P
ACTTGGAGAGAGGCTGGCCGTGATGAATTCGATTCATCAAAGCGAGTCATACACGGCTCT
CCTCTCTTTTAGT
        10        20         30
   U         CU       -|   A    AUUCA
ACU GGAGAGAGG  GGCCGUG AUGA UUCG     \
UGA UUCUCUCC  UCGGCAC UACU GAGC     U
   U        UC       A^   -    GAAAC
  70        60        50         40

mml-miR-485-3p
        10        20         30
   U         CU       -|   A    AUUCA
ACU GGAGAGAGG  GGCCGUG AUGA UUCG     \
UGA UUCUCUCC  UCGGCAC UACU GAGC     U
   U        UC       A^   -    GAAAC
  70        60        50         40

mml-miR-486
GTATCCTGTACTGAGCTGCCCCGAGCTGGGCAGCATGAAGGGCCTCGGGGCAGCTCAGTA
CAGGATGC
        10        20         30
                        -|  GGG AG
GUAUCCUGUACUGAGCUGCCCCGA GCU   C  \
CGUAGGACAUGACUCGACGGGGCU CGG   G  C
                        C^  GAA UA
       60        50        40

mml-miR-487a
GGTACTTGGAGAGTGGTCATCCCTGctgTGTTCGcTTTGtTtATGaCGAATCaTACAGGG
aCATCCAgTTTTTCAGTATC
        10        20        30         40
|     U     G   UCA      c    -    cU   u
GGUACU GGAGA UGG   UCCCUG ugUG UUCG  UUG U
CUAUGA UUUUU ACC   aGGGAC AUaC AAGC  aGU u
^     C     g   UAC      -    U    --   A
.        70        60         50

mml-miR-487b
TTGGTACTTGGAGAGTGGTTATCCCTGTCCTGTTCGTTTTGCTCgTGTCG
AATCGTACAGGGTCATCCACTTTTTCAGTATCAA
        10        20        30        40
U|      U         UUAU      CCU     UUU GC
 UGGUACU GGAGAGUGG    CCCUGU   GUUCG   U  U
 ACUAUGA UUUUUCACC    GGGACA   UAAGC   G  C
A^      C         UACU      UGC     U-- Ug
```

```
   80        70        60        50
```

mml-miR-488

GAGAATCATCTCTCCCAGATAATGGCACTCTCAAACAAGTTTCCAAgTTGTTTGAAAGGC
TATTTCTTGGTCAGATGACTCTC

```
        10        20        30        40
|   A      CU  C   U      A  C        G  U
GAGA UCAUCU  CC AGA AAUGGC CU UCAAACAA UU \
CUCU AGUAGA  GG UCU UUAUCG GA AGUUUGUU AA C
^   C      CU  U   -      -  A        g  C
  80        70         60         50
```

mml-miR-489

GTGGCAGCTTGGTGGTCGTATGTGTGgCGCCATTTACTTGAACCTTTAGGAGTGACATCA
CATATACGGCAGCTAAACTGtTAC

```
   10        20        30        40
|      C     G             CGC    UA      A
GUGGCAG UUGGU GUCGUAUGUGUGg   CAUU  CUUGA C
CAUuGUC AAUCG CGGCAUAUACACU   GUGA  GGAUU C
^      A     A             ACA    --      U
   80        70        60          50
```

mml-miR-490

TGGAGGCCTTGCTGGTTTGGAAAGTTCATTGTTCGACACCATGGATCTCCAGGTGGGTCA
AGTTTAGAGATGCACCAACCTGGAGGACTCCATGCTGTTGAGCTGTTCACAAGCAGCGGA
CACTTCCA

```
         10         20        30        40          50        60
U     CC-|     G    -   AGUUCAUU        C      --         G   CAA   U
 GGAGG   UUGCUG UUUG GAA        GUUCGACA CAUGGA  UCUCCAGGU GGU   GUU A
 CCUUC   GGCGAC GAAC CUU        CGAGUUGU GUACCU  GGAGGUCCA CCA   UAG G
A     ACA^     -    A   GU------        C      CA         A   CG-   A
      120        110              100        90         80          70
```

mml-miR-491

TTGACTTAGCTGGGTAGTGGGGAACCCTTCCATGAGGAGTAGAACACTCCTTATGCAAGA
TTCCCTTCTACCTGGCTGGGTTGG

```
        10        20        30        40
|UG       UG     U      CC   C          AG
U  ACUUAGC  GGUAG GGGGAA  CUU CAUGAGGAGU  \
G  UGGGUCG  CCAUC UCCCUU  GAA GUAUUCCUCA  A
^GU       GU     U      A-   C          CA
   80        70        60         50
```

mml-miR-492

ACTACAGCCACTACTACAaGACCtTCGAGGACCTGCGGGACAAGATTCTTGGTGCCgtCA
aTGAGAACtCCAGGATTGTCctgCAGATCAACAATGCCTGTCTGGCTGCAGATG

```
          10        20           30        40        50        60
A--  A     ACUACUACA|   CuUCGAG---  C           GAU      UGCCg  Aa
   CU CAGCC         aGAC          GA CUGCGGGACAA   UCUUGG     uC  \
   GA GUCGG         UCUG          CU GACgucCUGUU   AGGACC     AG  U
GUA  C     ---------^   UCCGUAACAA  A           ---      uCA--  AG
  110                100        90        80            70
```

mml-miR-493-5p

CTGGCCTCCAGGGCTTTGTACATGGTAGGCTTTCATTCATTCGTTTGCACATTCGGTGAA
GGTCTACTGTGTGCCAGGCCCTGTGCCAG

```
        10        20        30        40
|    CUC        U                    CAUUC  U
CUGGC   CAGGGCUU GUACAUGGUAGGCUUUCAUU     GU U
```

```
GACCG   GUCCCGGA CGUGUGUCAUCUGGAAGUGG     CA G
^    U--        C                    CUUA-  C
          80        70        60         50
```

mml-miR-493-3p

```
        10        20        30        40
|    CUC        U                    CAUUC  U
CUGGC   CAGGGCUU GUACAUGGUAGGCUUUCAUU     GU U
GACCG   GUCCCGGA CGUGUGUCAUCUGGAAGUGG     CA G
^    U--        C                    CUUA-  C
          80        70        60         50
```

mml-miR-494

GATACTCGAAGGAGAGGTTGTCCGTGTTGTCTTCTCTTTATTTATGATGAAACATACACG
GGAAACCTCTTcTTTAGTATC

```
        10        20         30         40
|     C           GU       -   C   -  UUUA
GAUACU GAAGGAGAGGUU  CCGUGU UGU UUC UC    \
CUAUGA UUUcUUCUCCAA  GGCACA ACA AAG AG    U
^     -           AG       U   -   U  UAUU
80         70        60         50
```

mml-miR-495

TGGTACCTGAAAAGAAGTTGCCCATGTTATTTTCGCTTTATATGTGACGAAACAAACATG
GTGCACTTCTTTTTCGGTATCA

```
        10        20         30         40
U|     U          U  -       AU      C  UAU
 GGUACC GAAAAGAAGU GC CCAUGUU  UUUCG UU   \
 CUAUGG UUUUUCUUCA CG GGUACAA  AAAGC AG   A
A^     C          -  U       AC      -  UGU
```

mml-miR-496

CCCgAGTCAGGTACTCGAATGGAGGTTGTCCATGGTGTGTTCATTTTATT
TATGATGAGTATTACATGGCCAATCTCCTTTCGGTACTCAATTCTTCTTG
GG

```
             10        20        30        40
CCC-----|   CA     U    U        U     GU         UUA
        gAGU  GGUAC CGAA GGAGGUUG CCAUG  GUGUUCAUU   U
        CUUA  UCAUG GCUU CCUCUAAC GGUAC  UAUGAGUAG   U
GGGUUCUU^   AC     -    U        C     AU         UAU
100        90         80        70         60        50
```

mml-miR-497

CCACCCCGGTCCTGCTCCCGCCCCAGCAGCACACTGTGGTTTGTACGGCACTGTGGCCAC
GTCCAAACCACACTGTGGTGTTAGAGCGAGGGTGGGGGAGGCACCGCCGAGG

```
        10            20               30        40          50
CCA  C    ----   G         C------     G    C         U  --|  AC
   CC CGGU    CCU CUCCCGCCC       AGCA CACA UGUGGUUUG AC  GGC  U
   GG GCCG    GGA GGGGGUGGG       UUGU GUGU ACACCAAAC UG  CCG  G
---  A    CCAC   -         AGCGAGA     G    C         C  CA^  GU
   110       100         90        80         70        60
```

mml-miR-498

AAtCCTCCTTGGGAAGTGAAGCTCAGGCTGTGATTTCAAGCCAGGGGGCGTTTTTCTgTgACTGGATGAA
AAGCACCTCCgGgGCTTGAAGCTCACAGTTTGAGAGCAATCaTCTAAGGAAGTT

```
        10         20         30         40         50         60
AAUCC        AAG-   AAG            -         A-|    C       UGUGA
     UCCUUGGG    UG   CUCAGGCUGUGA UUUCAAGCC  GGGGG GUUUUUC      \
```

```
     AGGAAUCU    AC   GAGUUUGACACU GAAGUUCGG  CCUCC CGAAAAG     C
UUGA-        ACUA  GA-            C         GG^    A       UAGGU
   120       110        100        90        80        70
```

mml-miR-499-5p

GCCCTGTCCCCGTGtCTTGGGCGGGCaGCTGTTAAGACTTGCAGTGATGT
TTAACTCCTCTCCACGTGAACATCACAGCAAGTCTGTGCTGCTTCCCGTC
CCTACGCTGCCTGGGCAGGGT

```
        10           20        30        40        50
         CC---|   CUU       C   U  UA        A          ACUCCU
GCCCUGUCC     GUGu   GGGCGGG aGC GU  AGACUUGC GUGAUGUUUA      \
UGGGACGGG     CGCA   CCUGCCC UCG CG  UCUGAACG CACUACAAGU      C
         UCCGU^   UC-       U   U  UG        A          GCACCU
 .       110        100        90        80        70
```

mml-miR-499-3p

```
        10           20        30        40        50
         CC---|   CUU       C   U  UA        A          ACUCCU
GCCCUGUCC     GUGu   GGGCGGG aGC GU  AGACUUGC GUGAUGUUUA      \
UGGGACGGG     CGCA   CCUGCCC UCG CG  UCUGAACG CACUACAAGU      C
         UCCGU^   UC-       U   U  UG        A          GCACCU
 .       110        100        90        80        70
```

mml-miR-500

GCTCCCCCTCTCTAATCCTTGCTACCTGGGTGAGAGTGCTaTCTGAATGC
AATGCACCTGGGCAAGGATTCTGAGAGCGAGAGC

```
       10         20        30        40
    CCC     U-|         ACC      AGAG   UaUC
GCUC   CUCUC  AAUCCUUGCU   UGGGUG    UGC    \
CGAG   GAGAG  UUAGGAACGG   GUCCAC    ACG    U
    AGC     UC^         ---      GUA-   UAAG
   80        70           60         50
```

mml-miR-500*

```
       10         20        30        40
    CCC     U-|         ACC      AGAG   UaUC
GCUC   CUCUC  AAUCCUUGCU   UGGGUG    UGC    \
CGAG   GAGAG  UUAGGAACGG   GUCCAC    ACG    U
    AGC     UC^         ---      GUA-   UAAG
   80        70           60         50
```

mml-miR-501-5p

GCTCTTCCTCTCTAATCCTTTGTCCCTGGGTGAGAGTGCTTTCTGAATGC
AgTGCACCCaGGCAAGGATTCTGAGAGGGTGAGC

```
        10         20        30        40
    U       U-|      UGUC        AGAG   U  C
GCUC UCCUCUC  AAUCCUU    CCUGGGUG    UGC UU \
CGAG GGGAGAG  UUAGGAA    GGaCCCAC    ACG AA U
    U       UC^      C---        GUg-   U  G
   80        70           60         50
```

mml-miR-501-3p

```
        10         20        30        40
    U       U-|      UGUC        AGAG   U  C
GCUC UCCUCUC  AAUCCUU    CCUGGGUG    UGC UU \
CGAG GGGAGAG  UUAGGAA    GGaCCCAC    ACG AA U
```

```
    U       UC^      C---        GUg-   U  G
   80        70           60         50
```

mml-miR-502-5p

CCCTCTCTAATCCTTGCTATCTGGGTGCTAGTGCTGtCTCAATGCAATGC
ACCTGGGCAAGGATTCAGAGAGGGGGAGCT

```
               10        20        30
------        -|         AUC       UAG   UGuC
      CCCUCUCU AAUCCUUGCU   UGGGUGC   UGC    \
      GGGAGAGA UUAGGAACGG   GUCCACG   ACG    U
UCGAGG        C^         ---       UA-   UAAC
.        70        60            50        40
```

mml-miR-502-3p

```
               10        20        30
------        -|         AUC       UAG   UGuC
      CCCUCUCU AAUCCUUGCU   UGGGUGC   UGC    \
      GGGAGAGA UUAGGAACGG   GUCCACG   ACG    U
UCGAGG        C^         ---       UA-   UAAC
.        70        60            50        40
```

mml-miR-503

TGCCCTAGCAGCGGGAACAGTTCTGCAGTGAGtGATCaGTACTCTGGaGTATTGTTTCCG
CTGCCAGGGTA

```
        10        20             30
      A              U  G   -----|  G
UGCCCU GCAGCGGGAACAGU CU CAG     UGA u
AUGGGA CGUCGCCUUUGUUA Ga GUC     aCU G
      C              U  G   UCAUG^  A
70        60        50        40
```

mml-miR-504

GCTGCTGTTGGGAGACCCTGGTCTGCACTCTATCTGTATTCTTACTGAAGGGAGcGCAGGG
CAGGGTTTCCCATACAGAGGGC

```
           10        20        30         40
G--  G    -            G     A    A-| U   U
   CU CUGU UGGGAGACCCUG UCUGC CUCU  UC GUA U
   GA GACA ACCCUUUGGGAC GGACG GAGG  AG CAU C
CGG  -    U            G     c    GA^ U   U
  80         70        60        50
```

mml-miR-505

GATGCACCCAGTGGGGGAGCCAGGAAGTATTGATGTTTCTGCCAGTTTAG
CGTCAACACTTGCTGGTTTCCTCTCTGGAGCATC

```
        10        20        30        40
GA|  AC    U           G    A        UCUGC
  UGC  CCAG GGGGGAGCCAG AAGU UUGAUGUU     \
  ACG  GGUC CUCCUUUGGUC UUCA AACUGCGA     C
CU^  A-    U           G    C        UUUGA
   80         70        60        50
```

mml-miR-506

GCCACCACCATCAGCCATgCTATGTGTAGTGCCTTATTCAGGAAGGTGTTACTTAATAtATTAAT
ATTTGTAAGGCACCCTTCTGAGTAGAGTAATGTGCAACATGGACAtCATTTGTGGTGGC

```
        10           20        30        40          50        60
GC      CAUCAG---|    CUA  UG  G   C          A     --    UU      A
  CACCAC         CCAUg   UG  UA UGC UUAUUCAGGA GGUGU  UAC  AAUAu U
```

```
  GUGGUG         GGUAC   AC  GU AUG GAUGAGUCUU CCACG  AUG  UUAUA U
CG      UUUACuACA^    A--  GU  A   A          C     GA   U-      A
  120       110         100        90         80        70
```

mml-miR-507

GTGCTGTGTGTAGTGCTTCACTTCAAtAAGTGCCATtCATGTGTCTAGAA
ATATGTTTTGCACCTTTTGGAGTGAAATAATGCACAACAGgTAC

```
          10        20        30        40
   -   -|     G  C          uAA    CAUu     GUC
GUG CUG UGUGUA UG UUCACUUCAA   GUGC    CAUGU   U
CAU GAC ACACGU AU AAGUGAGGUU   CACG    GUAUA   A
   g   A^     A  A          UUC    UUUU     AAG
   90        80        70        60        50
```

mml-miR-508

CCAtCTTCAGCTGAGTGTcGTGCtCTACTCCAGAGGGCGTCACTCAcaTAAACTAAAACA
TGATTGTcGCCTTTTTGAGTAGAGTAATACACATCACGTAAgGCATATTTGGTGG

```
          10               20        30        40        50
     UUCA--  ------   -|   cG          C        U  C   caUAAA
CCAuC      GC       UGA GUGU  UGCuCUACUC AGAGGGCG CA UCA       C
GGUGG      CG       ACU CACA  AUGAGAUGAG UUUUCCGc GU AGU       U
     UUUAUA  gAAUGC   A^   UA          U        U  U   ACAAAA
   110       100        90        80        70
```

mml-miR-509-1

CATGCTGTGTGTGGTACCCTACTaCAGgCAGTGGCAATCATGTATAgTTAAAAATGATTG
GTAtGTCTGTGGGTAGAGTAaTGCATGACACATG

```
        10        20         30        40
    C   -    GG   C   -|        G  G       GUAUA
CAUG UGU GUGU  UAC CUA CUaCAGgCA UG CAAUCAU     g
GUAC ACA UACG  AUG GAU GGUGUCUGu AU GUUAGUA     U
    -   G    Ua   A   G^        -  G       AAAAU
    90        80        70         60        50
```

mml-miR-509-2

CATGtTGTGTGTGGTACCCTACTGCAGgCAGTGGCAATCATGTATAgTTAAAAATGATTG
GTAtGTCTGTGGGTAGAGTAaTGCATGACACATG

```
         10        20         30        40
    u   -    GG   C   -|UG     G  G       GUAUA
CAUG UGU GUGU  UAC CUA C  CAGgCA UG CAAUCAU     g
GUAC ACA UACG  AUG GAU G  GUCUGu AU GUUAGUA     U
    -   G    Ua   A   G^GU     -  G       AAAAU
    90        80        70         60        50
```

mml-miR-510

GTGGTaTCCTACTCCGGAGAGTGGCAATCACATaTAATTAaGTGTGATTGAAACCTCTAA
GAGTGGAGTAACAC

```
        10         20        30
   G  UC      C-|   A  GG         aUA
GUG Ua  CUACUC  GGAG GU  CAAUCACAU   A
CAC AU  GGUGAG  UCUC CA  GUUAGUGUG   U
   A  GA      AA^   -  AA         aAU
   70        60         50        40
```

mml-miR-511-1

CAATAGACACCCAcCtTGTCTTTTGCTCTGCAGTCAGTAAATATTTTTTTGTGAATGTGT
AGCAAAAGACAGAATGGgGGTCCATTG

```
        10        20        30        40
|   A   A    cC            CU   G   G    UA
```

```
CAAU GAC CCCA  uUGUCUUUUGCU  GCA UCA UAAA  U
GUUA CUG gGGU  GACAGAAAACGA  UGU AGU GUUU  U
^   C   G    AA           UG   A   -    UU
      80        70        60          50
```

mml-miR-511-2

CAATAGACACCCAcCtTGTCTTTTGCTCTGCAGTCAGTAAATATTTTTTTGTGAATGTGT
AGCAAAAGACAGAATGGgGGTCCATTG

```
        10        20        30        40
CA| A   A    cC           CU   G   G    UA
  AU GAC CCCA  uUGUCUUUUGCU  GCA UCA UAAA  U
  UA CUG gGGU  GACAGAAAACGA  UGU AGU GUUU  U
GU^ C   G    AA           UG   A   -    UU
      80        70        60          50
```

mml-miR-512-1-5p

TCTCAcTCTGTGGCACTCAGCCTcGgGGGCACTTTCTGGTGtCAGAATGA
AAGTGCTGTCATtGCTGAGaTCCAATGACTGAGG

```
        10        20        30        40
|    c  UG   CA      CUc  G         UGGUG
UCUCA UC  UGG  CUCAGC   Gg GGCACUUUC     u
GGAGU AG  ACC  GAGUCG   CU UCGUGAAAG     C
^    C  UA   Ua      uUA  G         UAAGA
   80        70        60        50
```

mml-miR-512-1-3p

```
        10        20        30        40
|    c  UG   CA      CUc  G         UGGUG
UCUCA UC  UGG  CUCAGC   Gg GGCACUUUC     u
GGAGU AG  ACC  GAGUCG   CU UCGUGAAAG     C
^    C  UA   Ua      uUA  G         UAAGA
   80        70        60        50
```

mml-miR-512-2-5p

GGTACTTCTCAcTCTGTGGCACTCAGCCTcGgGGGCACTTTCTGGTGtCA
GAATGAAAGTGCTGTCATtGCTGAGaTCCAATGACTGAGGCGAGCACC

```
          10        20        30        40
   A  --|    c  UG   CA      CUc  G         UGGUG
GGU CU  UCUCA UC  UGG  CUCAGC   Gg GGCACUUUC     u
CCA GA  GGAGU AG  ACC  GAGUCG   CU UCGUGAAAG     C
   C  GC^    C  UA   Ua      uUA  G         UAAGA
       90        80        70        60        50
```

mml-miR-512-2-3p

```
          10        20        30        40
   A  --|    c  UG   CA      CUc  G         UGGUG
GGU CU  UCUCA UC  UGG  CUCAGC   Gg GGCACUUUC     u
CCA GA  GGAGU AG  ACC  GAGUCG   CU UCGUGAAAG     C
   C  GC^    C  UA   Ua      uUA  G         UAAGA
       90        80        70        60        50
```

mml-miR-513-1

GGGATGCCACATTCAGCCATTCAGtGTACAGTGCCTTTCACAGGGAGGTGTCATTTATGT
GAACTAAAATATAAATTTCACCTTTCTGAGAAGaGTAATGTACAGCATGCACTGCATATG
TGGTGTCCC

```
        10        20          30        40        50        60
            UCA  CAUU   - -|      G   C    A   GG     UC        GAA
```

```
GGGAUGCCACAU   GC    CA G uGUACA UGC UUUC CAG  AGGUG  AUUUAUGU   C
CCCUGUGGUGUA   CG    GU C ACAUGU AUG GAAG GUC  UCCAC  UAAAUAUA   U
            UA-  UCAC  A G^     A   a    A   UU     UU        AAA
       120        110       100        90        80        70
```

mml-miR-513-2

GGGATGCCgCATTCAGCCATTCAGTGgtgTaCAGTGCCTTTCACAGGGAGGTGTCATTTA
TGTGAACTAAAcTATAAAtgtCACCTTTCTGcGAAGGGTAATGTACAtCATGCACTGCAT
ATGTGGTGTCCC

```
        10        20        30        40        50        60
|           UCA  CAUUCA          G        A   GG     U       -  GAA
GGGAUGCCgCAU   GC      GUGgugUaCA UGCCUUUC CAG  AGGUG CAUUUAU GU   \
CCCUGUGGUGUA   CG      UACuACAUGU AUGGGAAG GUC  UCCAC guAAAUA cA   C
^           UA-  UCACG-          A        c   UU     u       U  AAU
130       120         110       100        90        80        70
```

mml-miR-513-3

GGGATGCCACATTCAcCCATTtAcTGTaCAtTGCCTTTCACAGGGAGGTGTCATTTATGT
GAACTAAAcTATAAATgTCACtTTTCTGAGAAGaGTAATGTACAGCATGCACTGCATATG
TGGTGTCCC

```
       10           20        30        40        50         60
            U--| cC-   UuA           C    A   GG     U       -  GAA
GGGAUGCCACAU   CA   CAU   cUGUaCAuUGC UUUC CAG  AGGUG CAUUUAU GU   \
CCCUGUGGUGUA   GU   GUA   GACAUGUAAUG GAAG GUC  UuCAC gUAAAUA cA   C
            UAC^ CAC   C--           a    A   UU     U       U  AAU
       120       110         100        90        80        70
```

mml-miR-513b-1

GGGATGCCACATTCAGCCATTCAGtGTgCAGTGCCTTTCACAaGGAGGTGTCATTTATGTGA
ACTAAAcTATAAATgTCACCTTTtTGgGAAGaGTAATGTACAaCATGCACTGCATATGTGGT
GTCCCt

```
         10           20        30         40        50         60
-            U--   C-|  UCAG      G   -     A          U       -  GAA
 GGGAUGCCACAU   CAG  CAU    uGUgCA UGC CUUUC CAaGGAGGUG CAUUUAU GU   \
 CCCUGUGGUGUA   GUC  GUA    ACAUGU AUG GAAGg GUuUUCCAC  gUAAAUA cA   C
u            UAC   AC^  Ca--      A   a     -          U       U  AAU
.       120       110         100        90         80        70
```

mml-miR-513b-2

GGGATGCCACATTCAGCCATTCgGttTACAGTGCCTTTCACAaGGAGGTGTCATTTATGTGAACTAAAcT
ATAAATgTCACCTTTtTGgGAAGaGTAATGTACAaCATGCACTGCAaATGTGGTGTCCC

```
        10          20         30         40        50         60
             --   CCAUUCg|  -    G   -     A          U       -  GAA
GGGAUGCCACAUU  CAG       Guu UACA UGC CUUUC CAaGGAGGUG CAUUUAU GU   \
CCCUGUGGUGUAa  GUC       CaA AUGU AUG GAAGg GUuUUCCAC  gUAAAUA cA   C
             AC   ACGUA--^  C    A   a     -          U       U  AAU
       120       110         100        90         80        70
```

mml-miR-514-1

AACATGTTGTCTGTGGTACCCTACTCTGGAGAGTGACAATCATGTATAATTAAATTTGAT
TGACACTTCTGTGAGTAGAGTAAtGCATGACACGTgCG

```
        10        20          30        40
AA    U    -   GG   C      -|   G    A      U  AUA
  CAUG UGUC UGU   UAC CUACUC UGGA AGUG CAAUCA GU   A
  gUGC ACAG ACG   AUG GAUGAG GUCU UCAC GUUAGU UA   U
GC    -    U   uA   A      U^   -    A      U  AAU
        90        80        70         60        50
```

mml-miR-514-2

GTTGTCTGTGGTACCCTACTCTGGAGAGTGACAATCATGTATAAtTAAATTTGATTGACA
CTTCTGTGAGTAGAGTAAtGCATGACAC

```
         10        20         30        40
GUU   -   GG   C      -|   G    A      U  AUA
   GUC UGU  UAC CUACUC UGGA AGUG CAAUCA GU   A
   CAG ACG  AUG GAUGAG GUCU UCAC GUUAGU UA   u
CA-   U   uA   A      U^   -    A      U  AAU
        80        70         60        50
```

mml-miR-516a-1-5p

TCTCAGGCTGTGACCgTCTCGAGGAAAGAAGCACTTTCTGTTGTCTAAAGAAAAGgAAGT
GtTTCCTTcCcGAGGGTTACGGTTTGAGA

```
        10        20        30        40
|              gU    A    A            GUUG  U
UCUCAGGCUGUGACC  CUCG GGAA GAAGCACUUUCU    UC A
AGAGUUUGGCAUUGG  GAGc CcUU CUUuGUGAAgGA    AG A
^              --    -    C            AA--  A
        80           70        60           50
```

mml-miR-516a-1-3p

```
        10        20        30        40
|              gU    A    A            GUUG  U
UCUCAGGCUGUGACC  CUCG GGAA GAAGCACUUUCU    UC A
AGAGUUUGGCAUUGG  GAGc CcUU CUUuGUGAAgGA    AG A
^              --    -    C            AA--  A
        80           70        60           50
```

mml-miR-516a-2-5p

TCTCAGGcTGTGACCgTCTCGAGGAAAGAAGCACTTTCTGTTGTCTAAAGAAAAGgAAGT
GtTTCCTTcCcGAGGGTTACGGTTTGAGA

```
        10        20        30        40
|              gU    A    A            GUUG  U
UCUCAGGcUGUGACC  CUCG GGAA GAAGCACUUUCU    UC A
AGAGUUUGGCAUUGG  GAGc CcUU CUUuGUGAAgGA    AG A
^              --    -    C            AA--  A
        80           70        60           50
```

mml-miR-516a-2-3p

```
        10        20        30        40
|              gU    A    A            GUUG  U
UCUCAGGcUGUGACC  CUCG GGAA GAAGCACUUUCU    UC A
AGAGUUUGGCAUUGG  GAGc CcUU CUUuGUGAAgGA    AG A
^              --    -    C            AA--  A
        80           70        60           50
```

mml-miR-517a

CTCAtGCAGTGACCCTCTAGATGGAAGCACTGTCTGTgGTcTAAAAGAAA
AGATCGTGCATCCtTTTAGAGTGTTACcGTTTGAGA

```
        10        20        30        40
-|   u  A     C       U   A    U    GUgG   A
 CUCA GC GUGAC CUCUAGA GGA GCAC GUCU    UcU \
 GAGU UG CAUUG GAGAUUU CCU CGUG UAGA    AGA A
A^   U  c     U       u   A    C    AA--   A
     80        70        60        50
```

mml-miR-517b

GTGACCCTCTAGATGGAAGCACTGTCTGTgGTCTaaAAGAAAAGATCGTG

CATCCtTTTAGAGTGTTAC

```
        10        20        30
|    C       U   A    U    GUgG   a
GUGAC CUCUAGA GGA GCAC GUCU    UCU \
CAUUG GAGAUUU CCU CGUG UAGA    AGA a
^    U       u   A    C    AA--   A
        60        50          40
```

mml-miR-518a-1

TCTCAtGCTGTGACccTaCAAAGGGAAGCCCTTTCTGTTGTCTaAAcGAAaAGAAAGtGC
TTCtCTTTGCTGGgTTACGGTTTGAGA

```
        10        20        30        40
     u         Ua          C      G-|   C
UCUCA GCUGUGACcc  CAAAGGGAAGC CUUUCU  UUGU U
AGAGU UGGCAUUgGG  GUUUCuCUUCG GAAAGA  AGcA a
     U         UC          u      aA^   A
      80        70        60        50
```

mml-miR-518b

TCAgGCTGTGaCCCTCCAGAGGGAAGCaCTTTCTGTTGTCTGAAAGAAAgCAAAGCGCTCCCC
TTTAGAGGaTTACGGTTTGA

```
        10        20        30        40
UC|        C    C      A   a   UC    G  UG
  AgGCUGUGa CCUC AGAGGG AGC CUU  UGUU UC  \
  UUUGGCAUU GGAG UUUCCC UCG GAA  ACgA AG  A
AG^        a    A      C   C   --    A  AA
  80        70        60          50
```

mml-miR-518c*

GCGAGAAGATtTCATGCTGTGACTCTCTGGAGGGAAGCgCTTTCTGTTGTCTGAAAGAAAAC
AAAGCGCTTCTCTTTAGAGaGTTACGGTTTGAGAAAAGC

```
        10        20        30        40        50
GCGAGAAGA|    U                           UC    G  UG
         UuUCA GCUGUGACUCUCUGGAGGGAAGCgCUU  UGUU UC  \
         AGAGU UGGCAUUGaGAGAUUUCUCUUCGCGAA  ACAA AG  A
CGAAA----^    U                           --    A  AA
 .            90        80        70          60
```

mml-miR-518c

```
        10        20        30        40        50
GCGAGAAGA|    U                           UC    G  UG
         UuUCA GCUGUGACUCUCUGGAGGGAAGCgCUU  UGUU UC  \
         AGAGU UGGCAUUGaGAGAUUUCUCUUCGCGAA  ACAA AG  A
CGAAA----^    U                           --    A  AA
 .            90        80        70          60
```

mml-miR-518d

CATGCTGTGACtCTCTgGAGGGAAGCgCTTTCTGTTGTCTGAAAGAAAaCAAAGCGCTTC
tCTTTaGAGaGTTACGGTTTGAGA

```
           10        20        30        40
---| U                             UC    G  UG
   CA GCUGUGACuCUCUgGAGGGAAGCgCUU  UGUU UC  \
   GU UGGCAUUGaGAGaUUUCuCUUCGCGAA  ACaA AG  A
AGA^ U                             --    A  AA
   80        70        60          50
```

mml-miR-518e

TCTCAGGCTGTGACCCTCTAGAGGGAAGCGaTTTCTGTgatCTgAAAGAA
AAGAAAatGgTTCCCTTtAGAGTGTTActgTTTGAGA

```
        10        20        30        40
UC|     U     C            G  a     GUga  Ug
  UCAGGC GUGAC CUCUAGAGGGAA CG UUUCU    uC  \
  AGUUUg cAUUG GAGAuUUCCCUU Gu AAAGA    AG  A
AG^     u     U            g  a     AA--  AA
      80        70        60        50
```

mml-miR-518f*
TCTCAgGCTGTGACCCTCTAGAGGGAAGCACTTTaTCTTGTgTgAAAGgAAAGAAAGCGC
TTCcCTTcAGAGGATTACTCTTTGAGA

```
        10        20        30          40
UC     CU    C     AG        A    a--|   GUg
  UCAgG   GUGA CCUCU  AGGGAAGC CUUU   UCUU   \
  AGUUU   CAUU GGAGA  UCcCUUCG GAAA   AgGA   U
AG     CU    A     cU        C    GAA^   AAg
      80        70        60        50
```

mml-miR-518f

```
        10        20        30          40
UC     CU    C     AG        A    a--|   GUg
  UCAgG   GUGA CCUCU  AGGGAAGC CUUU   UCUU   \
  AGUUU   CAUU GGAGA  UCcCUUCG GAAA   AgGA   U
AG     CU    A     cU        C    GAA^   AAg
      80        70        60        50
```

mml-miR-519a-1
CTCAGGCTGTGACcCTCTAGAGGGAAGCGCTTTCTGTgGTCTGAAAGAAA
aGAAAGTGCtTCCTTTTAGAGgGTTACcGTTTGAG

```
        10        20        30        40
CU|    U                           GUgG  UG
  CAGGC GUGACcCUCUAGAGGGAAGCGCUUUCU    UC  \
  GUUUG CAUUGgGAGAUUUUCCUuCGUGAAAGa    AG  A
GA^    c                           AA--  AA
    80        70        60        50
```

mml-miR-519b
CATGCTGTGACCCTCTgGAGGGAAGCGCTTTCTGTTGTCTGAAAGAAAAG
AAcGTGCATCCcTTTAGAGGgTTACTcTTTG

```
        10        20        30        40
CAUGCU|                A    U    GUUG  UG
      GUGACCCUCUgGAGGGA GCGC UUCU    UC  \
      CAUUgGGAGAUUUcCCU CGUG AAGA    AG  A
GUUUcU^                A    c    AA--  AA
80        70        60        50
```

mml-miR-519c
TCTCAGtCTGTGACCCTCTAGAaGGAAGCaCTTTCTGTTGTtTGAAAGAA
AAGAAAGTGCATCaTTTTAGAGGATTACAGTTTGAGA

```
        10        20        30        40
UC|   u      C         G  A         GUUG   G
  UCAG CUGUGA CCUCUAGAa GA GCaCUUUCU    UuU A
  AGUU GACAUU GGAGAUUUU CU CGUGAAAGA    AAG A
AG^   U      A         a  A         A---   A
```

```
      80         70         60           50
```

mml-miR-519d

```
TCCCAaGCTGTGACCCTCCAAAGGGAAGCaCTTTCTGTTTGTTgtctgag
agaAAACAAAGTGCtTCCtTTTAGAGTGTgACCGcTTGGGA
        10         20         30          40
UC   U  U  G  C    AG  a-         CUG-|  G  G
  CCA GC GU gC CUCU  AG  AAGCACUUU    UUU UU U
  GGU UG Cg ug GAGA  UC  UUCGUGAAA    AAg AG C
AG   U  U  g  U    cU  CC         CAAA^  G  U
.        80         70         60         50
```

mml-miR-520a

```
CTCAGGCTGTGACCCTCCAGAGGGAAGTAtTTTCTGTTGTCTGAagGAAAAGAAAGTGCT
TCCCTTTGGACTGTTTCGGTTTGAG
        10         20         30         40
|        U   CC                    GUUG  UG
CUCAGGCUG GAC  UCCAGAGGGAAGUAuUUUCU    UC  \
GAGUUUGGC UUG  AGGUUUCCCUUCGUGAAAGA    AG  A
^        U   UC                    AA--  Ga
    80         70         60         50
```

mml-miR-520a*

```
        10         20         30         40
|        U   CC                    GUUG  UG
CUCAGGCUG GAC  UCCAGAGGGAAGUAuUUUCU    UC  \
GAGUUUGGC UUG  AGGUUUCCCUUCGUGAAAGA    AG  A
^        U   UC                    AA--  Ga
    80         70         60         50
```

mml-miR-520b

```
CCCTCTAgAGGGAAGCGCTTTCTGTgGTCTGAAAGAAAAGAAAGTGCTTC
CTTTTAGAGGG
       10         20         30
|                       GUgG  UG
CCCUCUAgAGGGAAGCGCUUUCU    UC  \
GGGAGAUUUUCCUUCGUGAAAGA    AG  A
^                       AA--  AA
60         50         40
```

mml-miR-520c

```
TCTCAGGCTGTgacCCTCTAGAGGGAAGCgCTTTCTGTgGTCTGAAAGAA
AAGAAAGTGCTTCCTTTTAGAGGGTTACCGTTTGAGA
        10         20         30         40
|       U                         GUgG  UG
UCUCAGGC GUgacCCUCUAGAGGGAAGCgCUUUCU    UC  \
AGAGUUUG CAUUGGGAGAUUUUCCUUCGUGAAAGA    AG  A
^       C                         AA--  AA
      80         70         60         50
```

mml-miR-520d

```
TCTCAtGCTGTGAccCTACAAAGGGAAGCCCTTTCTGTTGTCTAAAcGAA
AAGAAAGTGCTTCTCTTTGcTGGGTTACGGTTTGAGA
        10         20         30          40
     u          UA           C      G-|   C
UCUCA GCUGUGAccC  CAAAGGGAAGC CUUUCU  UUGU U
AGAGU UGGCAUUGGG  GUUUCUCUUCG GAAAGA  AGcA A
```

```
     U          Uc           U      AA^   A
      80        70        60        50
```

mml-miR-520d*

```
        10        20        30          40
     u          UA           C      G-|   C
UCUCA GCUGUGAccC  CAAAGGGAAGC CUUUCU  UUGU U
AGAGU UGGCAUUGGG  GUUUCUCUUCG GAAAGA  AGcA A
     U          Uc           U      AA^   A
      80        70        60        50
```

mml-miR-520e

GCTGTGACCCTCtAGAgGGAAGCgcTTTCTGTgGTCTGAAAGaAAAGAAA
GTGCTTCCTTTTaGAGGGTTACcGTTTGAGA

```
              10        20        30
------| U                           GUgG  UG
      GC GUGACCCUCuAGAgGGAAGCgcUUUCU    UC  \
      UG CAUUGGGAGaUUUUCCUUCGUGAAAGA    aG  A
AGAGUU^ c                           AA--  AA
80        70        60        50          40
```

mml-miR-520f

TCTCAGGCTGTGACCCTCTAgAGGGAAGCGCTTTCTGTGGTCtGAAAGAAAAGaAAGTGC
TTCCTTTTAGAGGGTTACCGTTTGaGA

```
        10        20        30        40
|       U                           GUGG  uG
UCUCAGGC GUGACCCUCUAgAGGGAAGCGCUUUCU    UC  \
AGaGUUUG CAUUGGGAGAUUUUCCUUCGUGAAaGA    AG  A
^       C                           AA--  AA
      80        70        60        50
```

mml-miR-520g

TCCCATGCTGTGgCCCTCTAGAGaAAGCACTTTCTGTTTGTTGTCTGAGgAAAAACAAAG
TGCTTCCCTTcAGAGTGTggCtGTTTGGGA

```
        10        20         30         40
     U  U  G  C    AG  a-        CUG-|  G  G
UCCCA GC GU gC CUCU  AG  AAGCACUUU    UUU UU U
AGGGU UG Cg UG GAGA  UC  UUCGUGAAA    AAg AG C
     U  u  g  U    cU  CC        CAAA^  G  U
.        80        70        60        50
```

mml-miR-520h

TCCCAaGCTGTGACCCTCcaaagGGAAGCACTTTCTGTTTGTTGTCTGAGAgAAAACAAAG
TGCTTCCtTTTAGAgtgtg

```
        10        20        30        40
UCCCAaGCUGUG| C   c               CUG   G  GU
            AC CUC aaagGGAAGCACUUU   UUU UU  C
            ug gAG UUUuCCUUCGUGAAA   AAA AG  U
g-----------^ u   A               CA-   g  AG
.                  70        60         50
```

mml-miR-521

TCTCAtGCTGTGACCCTCCAAAGGGAAGtACTTTCTGTTGTCTAAAAGAAAAGAACGCAC
TTCCCTTTgGAGTGTTACCGTtTGAGA

```
        10        20        30        40
UC|  u  U     C              ACU    GUUG  UA
  UCA GC GUGAC CUCCAAAGGGAAGu   UUCU    UC  \
```

```
  AGU UG CAUUG GAGgUUUCCCUUCA   AAGA    AG  A
AG^  u  C     U              CGC    AA--  AA
      80        70        60        50
```

mml-miR-522

```
TCTCAGGCTGTGaCCCTCTAGAGGGAAGCGaTTTCTGTgaTCTGAAAGAAAAGAAAATGG
TTCCCTTTAGAGTGTTACtgTTTGAGA
        10        20        30        40
UC|     U     C            G  a     GUga  UG
  UCAGGC GUGaC CUCUAGAGGGAA CG UUUCU    UC  \
  AGUUUg CAUUG GAGAUUUCCCUU GU AAAGA    AG  A
AG^     u     U            G  A     AA--  AA
      80        70        60        50
```

mml-miR-523a

```
TCTCAGGCTGTGACCCTCtAGAGGGAAGcACTTTCTGTTGTCTGgaAGAA
AAGAAtGcGCTTCCCTTTAGAGGGTTACTCTcTGAGA
        10        20        30        40
|      CU                    ACU    GUUG  UG
UCUCAGG  GUGACCCUCuAGAGGGAAGc   UUCU    UC  \
AGAGUcU  CAUUGGGAGAUUUCCCUUCG   AAGA    AG  g
^      CU                    cGu    AA--  Aa
      80        70        60        50
```

mml-miR-523b

```
TCTCATGATGTGACCCTCTAGAGCGAAGCGCTTTCTGTTGGCTAGAAAAGAATAGGAAGC
GCTTCCCTTTAGAGTGTTACGCTTTGAGA
        10        20        30        40
|    UGA      C        C               GGCUA
UCUCA   UGUGAC CUCUAGAG GAAGCGCUUUCUGUU      \
AGAGU   GCAUUG GAGAUUUC CUUCGCGAAGGAUAA      G
^    UUC      U        C               GAAAA
        80        70        60        50
```

mml-miR-523c-1

```
CATGCTGTGACCCTCTgGAGGGAAGCGCTTTCTGTTGTCTGAAAGAAAAGAACGtGCaTCC
CTtTAGAGGGTTACtCTTTGAGA
           10        20        30        40
---| UGCU                 A    U    GUUG  UG
   CA     GUGACCCUCUgGAGGGA GCGC UUCU    UC  \
   GU     CAUUGGGAGAUuUCCCU CGuG AAGA    AG  A
AGA^ UUCu                 a    C    AA--  AA
   80        70        60        50
```

mml-miR-523c-2

```
TCCCATGCTGTGACCCTCTgGAGGgAAGCgCTTTCTGTTGTCTGAaaGAAAAgAAcGTGC
aTCCCTTTAGAGGGTTACTcTTTGaGAaga
           10        20        30        40
U---|C  UGCU                 A    U    GUUG  UG
    C CA     GUGACCCUCUgGAGGgA GCgC UUCU    UC  \
    G GU     CAUUGGGAGAUUUCCCU CGUG AAgA    AG  A
agaA^a  UUcU                 a    c    AA--  aa
.        80        70        60        50
```

mml-miR-525

```
CTCAGGCTGTGACTCTCCAGAGGGATGCACTTTCTTTTATGTGAAAAAAAAAGAAGGCGC
ATCCCTTTGGAGCGTTACGGTTTGGG
        10        20        30        40
```

```
|            U             A         AUGU
CUCAGGCUGUGAC CUCCAGAGGGAUGC CUUUCUUUU    G
GGGUUUGGCAUUG GAGGUUUCCCUACG GGAAGAAAA    A
^            C             C         AAAA
     80        70        60        50
```

mml-miR-525*

```
        10        20        30        40
|            U             A         AUGU
CUCAGGCUGUGAC CUCCAGAGGGAUGC CUUUCUUUU    G
GGGUUUGGCAUUG GAGGUUUCCCUACG GGAAGAAAA    A
^            C             C         AAAA
     80        70        60        50
```

mml-miR-532

CGACTTGCTTTCTCTCCTCCATGCCTTGAGTGTAGGACCGTTGGCATCTTAATTACCCTC
CCACACCCAAGGCTTGCA

```
        10        20        30        40
CGACUUGCUUUCUCUCCUC  U      A    A   CC  U----| C
                   CA GCCUUG GUGU GGA  GU     GG \
                   GU CGGAAC CACA CCU  CA     UC A
AC-----------------  U      C    C   CC  UUAAU^ U
                        70        60        50
```

mml-miR-539

ATACTTGAGGAGAAATTATCCTTGGTGTGTTCGCTTTATTTATGATGAATCATACAAGGA
CAATTTCTTTTTGAGTAT

```
        10        20         30
|    UG          A      G    -    C  UAU
AUACU  AGGAGAAAUU UCCUUG UGUG UUCG UU   \
UAUGA  UUUUCUUUAA AGGAAC AUAC AAGU AG   U
^    GU          C      -    U    -  UAU
       70        60         50
```

mml-miR-542-5p

CAGAcCTCAGACATCTCGGGGATCATCATGTCACGAGATACCAcTGTGCACTTGTGACAG
ATTGATAACTGAAAGGTCTGGGAGCCAtTCATCTTCA

```
                  10        20        30         40
CAGAc----------       AUC    GG    UCA         A-|  C
               CUCAGAC   UCGG  AUCA   UGUCACGAG  UAC A
               GGGUCUG   AGUC  UAGU   ACAGUGUUC  GUG c
ACUUCUACUuACCGA       GAA    AA    UAG         AC^  U
      90        80        70        60        50
```

mml-miR-542-3p

```
                  10        20        30         40
CAGAc----------       AUC    GG    UCA         A-|  C
               CUCAGAC   UCGG  AUCA   UGUCACGAG  UAC A
               GGGUCUG   AGUC  UAGU   ACAGUGUUC  GUG C
ACUUCUACUuACCGA       GAA    AA    UAG         AC^  U
      90        80        70        60        50
```

mml-miR-544

ATTTTCATCACCTAGGGATCTTGTTAAAAAGCAGATTCTGATTCAGGGACCAAGATTCTG
CATTTTTAGCAAGTTCTCAAGTGATGCTAAT

```
        10        20        30         40
AUUUU      CUA    U           -     U   GA-| C
     CAUCAC   GGGA CUUGUUAAAA  GCAGA UCU   UU A
```

```
     GUAGUG   CUCU GAACGAUUUUU CGUCU AGA   AG G
UAAUC      AA-    U           A     U   ACC^ G
90        80         70        60        50
```

mml-miR-545

CCCAGCCTGGCACATTAGTAGGCCTCAGTAAATGTTTATTAGATGAATAAATGAATGACT
CATCAGCAAACATTTATTGTGTGCCTGCTAAAGTGAGCTCCACAGG

```
            10          20         30         40         50
CCCAG    ----  A   --       CU-|           A  A     AUAAAU
     CCUG    GC CAU  UAGUAGGC   CAGUAAAUGUUU UU GAUGA      \
     GGAC    CG GUG  AUCGUCCG   GUUAUUUACAAA GA CUACU      G
-----    ACCU  A   AA       UGU^           C  -     CAGUAA
         100        90        80        70         60
```

mml-miR-548a

TcCAGGGAGGTATTAAGTTGGTGCAAAAGTAATTGTGgtTTTTTGCCATT
AAAAGTAAtGACAAtACTGGCAATTACTTTTcCtCCAAACCTGaTATT

```
               10        20        30        40
Uc---   ----|    UAUU     UGG   AAA    A    G   U   GCC
     CAG    GGAGG    AAGU   UGC   AGUA UUGU guU UUU    A
     GUC    CCuCc    UUCA   ACG   UCAu AACA uAA GAA    U
UUAUa   CAAA^    UU--     UUA   G--    -    G   U   AAU
       90        80          70           60        50
```

mml-miR-548b

CAGgCTATgTATTTAGGTTGGtGCAAAAGTAATTGgGGcTTgGGCCTTTA
TTTTCAATGGCAAaAACCTCAaTTGCTTTTGTGCCAACCTAATACTT

```
        10        20        30        40        50
CAGgCUAU|    U       uG                c  gG   UUUAU
        gUAUU AGGUUGG  CAAAAGUAAUUGgGG UU  GCC     U
        CAUAA UCCAACC  GUUUUCGUUaACUCC Aa  CGG     U
UU------^    -       GU                A  AA   UAACU
             90        80        70        60
```

mml-miR-548c

TGTGATGTATTAGGTTgaTGCAAAAGTAATTGGGGTTTTTTGtCaTTAAA
AGTAgTGaCAAAACcGGCAATTACTTcTGCACCAAACTAATATAA

```
        10        20        30         40        50
UGUGA        G   a    A         G-|    UU        A
     UGUAUUAG UUg UGCA AAGUAAUUG  GGUUU  UGuCaUUA A
     AUAUAAUC AAC ACGU UUCAUUAAC  cCAAA  ACaGUgAU A
A----        A   C    c         GG^    --        G
        90        80        70          60
```

mml-miR-548d

AAACAAGTTgTATTAGGTTGGTGCAAAAGTAATTGTGGTTcTTGCCTaTAAAAGTAATGG
CAAAAACCACAaTTTCTTTTGCACCAaACTAATAAAG

```
        10        20        30        40        50
AAACAAGUUg|     G            U          c     UaUAA
          UAUUAG UUGGUGCAAAAG AAUUGUGGUU UUGCC     A
          AUAAUC aACCACGUUUUC UUaACACCAA AACGG     A
GAA-------^     A            U          A     UAAUG
             90        80        70        60
```

mml-miR-548e

CCTAGAATGTTAcTAGGTtGGTGCAAAAGTAATTGCGAGTTTTACCATTA
CTTTCAATGGCAAAACcGGCAgTTACTTTTGCACCAACGTAATACTT

```
        10        20        30        40        50
CCUAGAA|     UAG                    GA     A     AC
       UGUUAc   GUuGGUGCAAAAGUAAUUGC  GUUUU CCAUU  U
       AUAAUG   CAACCACGUUUUCAUUgACG  CAAAA GGUAA  U
UUC----^     ---                    Gc     C     CU
          90           80        70        60
```

mml-miR-548f

ATTtAGGTTGGTGCAAAAGTAATTGCgGAtTTTGCCATTGAAAGTAATGGCCAAAACCAC
AGTTcCTTTTGCACCAATCTATAGA

```
         10        20        30        40
AUU-|               U     C  A   U       A
    uAGGUUGGUGCAAAAG AAUUG gG uUU GCCAUUG A
    AUCUAACCACGUUUUC UUGAC CC AAA CGGUAAU A
AGAU^               c     A  A   C       G
    80        70        60        50
```

mml-miR-549

AGACATGCAACTCAAGAATATATTGAGAGCTCATCCATAGTTGTCACTGTCTCAgATCAT
GACAAtTATGGATGAGCTCTTAATATATCCCAGGC

```
        10        20        30        40        50
AGACAUGCAACUCAAGA|                            CU   U
                 AUAUAUUGAGAGCUCAUCCAUAGUUGUCA  GUC \
                 UAUAUAAUUCUCGAGUAGGUAUuAACAGU  UAg C
CGGACCC----------^                            AC   A
    90                  80        70        60
```

mml-miR-550-1

TGATGCTTTGCTGGCTGGTGCAGTGCCTGAGGGAGTAAGAGCCCTGTTGTTGTAAGATAG
TGTCcTACTCCCTCAGGCACATCTCCAgCAAGT

```
        10        20        30        40        50
UGAUGCUUU|  G     UGCA              A AG C     GUU
         GCU GCUGG    GUGCCUGAGGGAGUA G  C CUGUU   \
         UGA CgACC    CACGGACUCCCUCAU c  G GAUAG   G
---------^  A     UCUA              - CU U     AAU
           90        80        70         60
```

mml-miR-551a

GGGGACTGCCGGGTGACCCTGGAAATCCAGAGTGGGTGGGGCCtGTCTGACCaTTTCTAG
GCGACCCACTCTTGGTTTCCAGGGTTGCCCTGGAAA

```
        10          20       30        40        50
GGGGACUG  -|  UG            C         GGG      UCUGA
        CC GGG  ACCCUGGAAAUC AGAGUGGGU   GCCuG     C
        GG CCC  UGGGACCUUUGG UCUCACCCA   CGGAU     C
AAA-----  U^  GU            U         G--      CUUUa
          90        80        70          60
```

mml-miR-551b

AGATGTGCTCTCCTGGCCCATGAAATCAAGCGTGGGTGAGACCTGGTGCA
GAACaGGAAGGCGACCCATACTTGGTTTCAGAGGCTGcGAGAATA

```
        10        20        30         40
AGAUGUGC    C     CA           CG      - AGA-|   GUG
        UCUC UGGCC  UGAAAUCAAG  UGGGU G    CCUG   C
        AGAG GUCGG  ACUUUGGUUC  ACCCA C    GGaC   A
AUA-----    c     AG           AU      G GGAA^   AAG
         90        80        70        60        50
```

mml-miR-552

ACCATTCAAATATACCACAGTTTGTTTgACCaTTaaCCTGTTTtGTTGAAG
ATGCCTTTCAACgGGTGACTGGTTAGACAAACTGTGGTATAttCA

```
        10        20        30        40        50
ACCAUUCA|                       UUa       uGUU    A
        AAUAUACCACAGUUUGUUUgACCa   aCCUGUU    GAAG U
        uuAUAUGGUGUCAAACAGAUUGGU   UGGgCAA    UUUC G
AC------^                       CAG       C---    C
          90        80        70        60
```

mml-miR-553

CTTCAATTTTATTTgAAAAaGGTGAGgTTTTGTTTTGTCTGAGAAAATCTCaCTGTTTTA
GACTGAGG

```
        10        20        30
|    AUUUUA       Aa           G  UU
CUUCA      UUUgAAA  GGUGAGgUUUU UU  G
GGAGU      AGAUUUU  UCaCUCUAAAA AG  U
^    C-----       G-           G  UC
            60         50        40
```

mml-miR-554

ACCTGAGTAACCTTTGCTAGTCCTGACTCAGCCAGTACTGaTCTTAcACTGGcagTGGGT
CAGGGTTCATATTTTGGCATCTCTCTCTGGGCATCT

```
             10        20                30          40
A-----  U   UAACCUU      --------          --|     ACUGa
      CC GAG       UGCUAG        UCCUGACUCA  GCCAGU     \
      GG CUC       ACGGUU        GGGACUGGGU  cGGUCA     U
UCUACG  U   UCUCU--      UUAUACUU          ga^     cAUUC
     90        80           70        60        50
```

mml-miR-556

GATAGTAATgAGAAAGATGAaCTCATTGTAATATGAGCTTCATTTATgCATTTCATATTA
CaATTAGCTgATCTTTTTTTTT

```
        10        20        30           40
GAUAGUAAU         GAa  C            ---| UUCA
         gAGAAAGAU   CU AUUGUAAUAUGA   GC    \
         UUUUUUCUA   GA UAaCAUUAUACU   Cg    U
UUU------         gUC  U            UUA^ UAUU
 80              70        60        50
```

mml-miR-557

AGAAUGGGCAAAUGAAtAGTAAATTTGGAGGCCTGGGGCCCTCCCTGCTG
CTGGAcAAGTGTcTGCAtGGGTGaGCCTTaTCTTTGAAAGGAGGTGGA

```
        10        20        30        40        50
AGAAUGGGCAAAUGAAuAGUAAA|       CC      CCU   UGC   U    A
                       UUUGGAGG  UGGGGC   CCC   UGC GGAc A
                       AAGUUUCU  aUUCCG   GGG   ACG UcUG G
GUGGAGGA---------------^       --      aGU   u--   -    U
     90                         80        70            60
```

mml-miR-558

GTGTGTGTGTGTGTtTGTGtTTATTTTGGcATAGTAGCTCTAGACTCTATTATAGTTTCC
TGAGCTGCTGTACCAAAATACCACAAACTGCCTG

```
        10        20        30        40        50
GUGUGUGU|  U       uU         c          UA  CU   U
        GUG GUuUGUG  UAUUUUGG AUAGUAGCUC  GA  CUA \
        CGU CAAACAC  AUAAAACC UGUCGUCGAG  CU  GAU U
GUC-----^  -       C-         A          UC  UU   A
        90          80        70        60
```

mml-miR-562

AGTGAAATTGCTgGGTCATATGGTCAGTCTACTTTcAGAGTAATTGTGAAAgTaTTTTTC
AAAGTAGCTGTACCATTTGCAtTCCCTGTGGCAAT

```
        10              20         30         40
AGUGAA      ---   U---  U     -|  U       c-      A  G
      AUUGCU   gGG    CA AUGGU CAG CUACUUU  AGAGUA UU U
      UAACGG   CCC    GU UACCA GUC GAUGAAA  UUUUaU AA G
------      UGU   UuAC  U     U^  -       CU      g  A
          90        80        70          60        50
```

mml-miR-563

AGCAAAGAAGTGTGTTGCCCTCcAGGAAATGTGTGTTGCTCTGATGTAAT
TAGGcTGACATACaTTTCCCTGGTAGCCA

```
       10        20        30         40
AGCAAAGAAG| U      CUCcA                 -  U     G
          UG GUUGCC     GGAAAUGUGUGUU GC CUGAU \
          AC CGAUGG     CCUUUaCAUACAG cG GAUUA U
----------^ -      UC---             U  -     A
                   70           60         50
```

mml-miR-567

GGATTCTTAcAGGACAcTATGTTCTTCCAGGACAGAACATTCTTTGCTATTTTGTACTGG
AAGAACATGCAAAACTttAAAAAAAGTTATTGCT

```
          10        20        30            40
.-GGAUUCUUACAGGACAcU            G      ---| UU
                    AUGUUCUUCCAG ACAGAA   CA  \
                    UACAAGAAGGUC UGUUUU   GU  C
\ ------------------            A      AUC^ UU
                           60        50
```

mml-miR-568

ATATACACTATATTATGTATAAATGTATACACACTTCCTATATGTATCCACATATATATA
GTGTATATATTATACATGTATAGGTGTGTATATG

```
            10        20        30        40
---       -|     U        A         ACUUCC       A
   AUAUACA CUAUAU AUGUAUAA UGUAUACAC      UAUAUGU U
   UAUGUGU GAUAUG UACAUAUU AUAUAUGUG      AUAUACA C
GUA       G^     -        -         AUAU--       C
   90        80         70         60           50
```

mml-miR-569

GGTATTGTTAGATTAATTTTGTGGGACATTAACAACAGCATCAGcAGCAACATCAGCTTT
AGTTAATGAATCCTGGAAAGTTAAGTGACTTTATTT

```
         10        20          30          40
-    UU    GA         G     --       .-AACA| A
 GGUA  GUUA  UUAAUUUU UGGGA  CAUUAAC      GC U
 UUAU  CAGU  AAUUGAAA GUCCU  GUAAUUG      cG C
U    UU    G-         G     AA       \ ----^ A
     90         80        70
```

mml-miR-570

TATTAGGTtGGTGCAAAcGTAATTGCAGTTTTTgCCATTAcTTTTAAagGCaAAAgtAGCAATT
ACCTTTGCACCAACCT

```
        10        20        30        40
UAUU|            c        AG        A   c
    AGGUuGGUGCAAA GUAAUUGC  UUUUUgCC UUA U
    UCCAACCACGUUU CAUUAACG  gAAAaCGg AAU U
```

```
----^            C        Au        a   U
   80        70        60        50
```

mml-miR-572

GTCGAGGCCGTGGCCCGGAAGTGaTCGGGGCCGCcGCGGaCGGAAGGGCG
CCTcTGCTTCGTCCGCTCGGCGGTGGCCCAGCCAGGCCCGCGGGA

```
        10         20        30        40          50
GUCGAGG    -|   C  AAGUGaUCG          G  -    A-     C
       CCGU GGCC GG         GGGCCGCcGC Ga CGGA  GGGCG C
       GGCG CCGG CC         CCCGGUGGCG CU GCCU  UUCGU U
AG-----    C^   A  GA-------          G  C    GC     c
         90        80               70        60
```

mml-miR-573

TTTAGAGGTGTCTCCCTGAAGTGATGCATAACCGATCAGGATCTACTCATGTCAACTTTG
GTAAAGTTATGTTGCTTGTCAGGGTGAGGAGAGGTTTTG

```
        10             20        30         40
UUUA  GG G   -----      AGUGAU       CG-|     AUCU  U
    GA  U UCU     CCCUGA      GCAUAAC   AUCAGG    AC C
    UU  G AGA     GGGACU      UGUAUUG   UGGUUU    UG A
G---  UU G   GGAGU      GUUCGU       AAA^     CAAC  U
           90        80        70        60        50
```

mml-miR-576

TACAATCCAgtGAGGATTCTAATTTCTCCACaTCTTTGGTAATAAGtTTT
GGCAAAGATGTGGAAAAATTGGAATCCTCATTgGATTGGTTATAA

```
             10        20        30        40
UA-----|                      C            G   UA
       CAAUCCAguGAGGAUUCUAAUUU UCCACaUCUUUG UAA  A
       GUUAGgUUACUCCUAAGGUUAAA AGGUGUAGAAAC GUU  G
AAUAUUG^                      A            G   Uu
    90        80        70        60        50
```

mml-miR-577

TGGGGGAaTGAAGAGTAGATAAAATATTGGTACCTGATGAgTgTGAGGCC
AGGTTTCAATACTTTATCTGCTCTTCATTTtCCCATATCTACTTAC

```
                   10        20        30        40
-----------|   GG                 A      U     AUGAgU
           UGGG  AaUGAAGAGUAGAUAAA UAUUGG ACCUG      \
           ACCC  UUACUUCUCGUCUAUUU AUAACU UGGAC      g
CAUUCAUCUAU^   uU                 C      U     CGGAGU
     90        80        70        60        50
```

mml-miR-578

GATAAATaTATAGACAAAATACAATCCtGGACtAtAAGAAGCTCCTATAG
CTCCTGTAGCTTCTTGTGCTCTgGGATTGTATTTTGTTTATATAT

```
        10        20        30        40        50
GAUAA|                     uG  C           CCU    C
     AUaUAUAGACAAAAUACAAUCC  GA uAuAAGAAGCU   AUAG \
     UAUAUAUUUGUUUUAUGUUAgG  CU GUGUUCUUCGA   UGUC U
-----^                     GU  C           ---    C
         90        80        70        60
```

mml-miR-579

CATATTAGGTTAATGCAAAAGTAATCGCGGTTTGTGCCAaATGgCGATTT
GAATTAATAAATTCATTTGGTAcAAACCGCGATTAcTTTTGCATCAgC

```
       10        20        30        40        50
```

```
CAUAUUAG|  A           C                   gC      AA
        GUU AUGCAAAAGUA UCGCGGUUUGUGCCAaAUG  GAUUUG  U
        CgA UACGUUUUcAU AGCGCCAAAcAUGGUUUAC  UUAAAU  U
--------^  C           U                   --      AA
               90        80        70         60
```

mml-miR-580

```
ATAAAATTTCCAgTTGGAACCTAATGATTCATCAGACTCAGATATTTAAG
TTAACAGTATTTGAGtcTGATGAATCATTAGGTTCCaGTCAGAAATT
        10        20        30        40        50
AUAA|     CAg                              UAAG
    AAUUUC   UUGGAACCUAAUGAUUCAUCAGACUCAGAUAUU    \
    UUAAAG   GaCCUUGGAUUACUAAGUAGUcuGAGUUUAUGA    U
----^     ACU                              CAAU
          90        80        70        60
```

mml-miR-581

```
GTTcTGTGAAcGTATTCTTGTGTTCTgTAGATCAGTGCTTTTAGAAAATT
TGTGTGATCTAgAGAACACAAAGAATACCTACACAGAACCAtCTGC
               10         20        30         40
-------        AAc      -|        g       G   UUUUAG
       GUUcUGUG   GUAUUC UUGUGUUCU UAGAUCA UGC      \
       CAAGACAC   CAUAAG AACACAAGA AUCUAGU GUG      A
CGUCuAC        AUC      A^        g       -   UUUAAA
     90        80        70        60         50
```

mml-miR-582

```
ATCTGTGCTCTTTGATTACAGTTGTTCAACCAGTTACTAATCTAcCTAAT
TGTAACTGGTTGAACAACTGAACCCAAAGGGTGCAAAGTAGAAACATT
                  10        20        30        40
AUC----------| UG      AUUA                  UAAUCU
             UG  CUCUUUG    CAGUUGUUCAACCAGUUAC      A
             AC  GGGAAAC    GUCAACAAGUUGGUCAAUG      c
UUACAAAGAUGAA^ GU      CCAA                  UUAAUC
       90        80        70        60        50
```

mml-miR-583

```
AACTCGCACATTtACCAAAGAGGAAGGTCCCAgTACTGCAGGGATCTTAGCAGTACTGGG
ACCTACCTCTTTGGT
        10        20        30         40
AACUCGCACAUUu         A                -|  G
             ACCAAAGAGG AGGUCCCAgUACUGC AGG \
             UGGUUUCUCC UCCAGGGUCAUGACG UUC A
-------------         A                A^  U
                 70        60        50
```

mml-miR-584

```
TAGGGTGACCAGCCATTATGGTTTGCCTGGGACTGAGGAATTTGCTGGGATATGTCAGTT
CCAGGCCAACCAGGCTGGTTGGTtTCCCTGAAGCAAC
             10        20        30        40
-------|    U         UUA     U         GGA    G
       UAGGG GACCAGCCA   UGGUU GCCUGGGACUGA   AUUU C
       GUCCC uUGGUUGGU   ACCAA CGGACCUUGACU   UAGG U
CAACGAA^    U         CGG     C         GUA    G
      90        80        70        60
```

mml-miR-586

```
ATGGGGTAAAACCATTATGCatATTGTATTTTTAGGTCCCAATACgTGTG
GaCCCTAAAAATgCAATGCATAATGGTTTTatACTCTTTATCTTCTTAT
                   10        20        30          40
AU-----------   -|                AU            -   -  AU
             GGG GUAAAACCAUUAUGCAU  UGUAUUUUUAGG UCC CA  \
             CUC UAUUUUGGUAAUACGUA  ACGUAAAAAUCC AGG GU  A
UAUUCUUCUAUUU   A^                --            C   U  GC
        90        80        70          60        50
```

mml-miR-587

```
CTCCTAgGCACCCTCTTTCCAcAGGTGATGAGTtACAGGGCcCAGGGAAT
GTGTCTGCACCTGTGACTCATCACtgGTGGAAGCCCATAC
       10        20        30           40        50
CUCCUA|  ACCCUCU      A                -  cCA   AA
      gGC       UUCCAc GGUGAUGAGUuACAGG GC   GGG  U
      CCG       AAGGUG uCACUACUCAGUGUCC CG   UCU  G
CAUAC-^  -------      g                A  ---   GU
.                80        70        60
```

mml-miR-589

```
TCCAGCCTGTGCCCAGCAGCCCCTGAGAACCACGTCTGCTCTGAGCTGGGTACTGCCTGT
TCAGAACAgAcGCtGcTTCCCAGACGCTGCCAGCTGGCC
         10        20        30         40        50
U-|    C  UGCCCA     CC   A   C  -      C       U   UA
  CCAGC UG       GCAGC  CUG GAA CA CGUCUG UCUGAGC GGG  \
  GGUCG AC       CGUCG  GAC CUU Gu GcAgAC AGACUUG UCC  C
CC^    -  ------     CA   C   c  C      A       -   GU
               90        80        70        60
```

mml-miR-590

```
TAGCCAGTCAGAAATGAGCTTATTCATAAAAGTGCAGTATGGTGgAGTCAgTCTGTAATT
TTATGTATAAGCTgGTCTCTaAcTGAAACgTGCAGCA
                10        20        30        40
UAGC--------|   C   A  G       U       G     UA   UG
            CAGU AGA AU AGCUUAU CAUAAAA UGCAG  UGG  \
            GUcA UCU UG UCGAAUA GUAUUUU AUGUC  ACU  g
ACGACGUgCAAA^   a   C  g       U       A     Ug   GA
      90        80        70        60        50
```

mml-miR-592

```
TATTATGCCATGACATTGTGTCAATATGCGATGATGTGTTGTGATGGCACAGCGTCATCA
CGTGGTGACGCAACATCATGACGTAAGACGTCACAAC
                 10        20        30        40
UA---------|    C     CA         A    C         GU     A
           UUAUG CAUGA  UUGUGUCA UAUG GAUGAUGU  UGUG U
           AAUGC GUACU  AACGCAGU GUGC CUACUGCG  ACAC G
CAACACUGCAG^    A     AC         G    A         --     G
      90        80        70        60          50
```

mml-miR-593

```
CCCCCAGAgTgTGTCAGGCAtCAGCCAGGCATcGCTCAGCCCcTTTCCCT
CTGGGGGAGCAAGGAGTGGTGCTGGGTTTGTCTCTGCTGGGGTTTCTCCT
               10        20        30            40
C-------      -| UgUGUC     uC    A          AG----     UUC
        CCCCAG Ag      AGGCA  AGCC GGCAUcGCUC      CCCcU   C
        GGGGUC UC      UCUGU  UUGG UCGUGGUGAG      GGGGG   C
UCCUCUUU      G^ ------     --    G          GAACGA     UCU
```

```
.        90              80          70         60         50
```

mml-miR-597

TACTTACTCTACaTGTGTGTCACTtGAcGACCACTGTGAAGAgAGTAAAA
TGTACAGTGGTTCTCTTGgGGCTCAAGCGTAAcGTAGAGTgCTGGTC

```
         10              20        30        40
U   --        ---    GU--|  A  u-  c          AAGAgA
 ACU  UACUCUAC   aUGU    GUC CU  GA GACCACUGUG      G
 UGG  gUGAGAUG   UGCG    CGG gG  CU UUGGUGACAU      U
C   UC        cAA    AACU^  -  UU  C          GUAAAA
      90        80        70         60        50
```

mml-miR-598

GCTTGATGATGCTGCTGATGCTGGCGGTGATCCCGATGGTGTGAGCTGGAAATGGGGTGC
TACGTCATCGTTGTCATCGTCATCATCATCATCCGAG

```
        10        20        30        40        50
G   -|    UGCUGC     C          CC     GU  G   UGGAA
 CUU GAUGA      UGAUG UGGCGGUGAU  CGAUG  GU AGC     A
 GAG CUACU      ACUAC ACUGCUACUG  GCUAC  CA UCG     U
-   C^    ------     U          UU     UG  -   UGGGG
       90              80        70          60
```

mml-miR-599

AAAGACATGCTGTCCACAGTGTGTTTGATAAGCTGACATGGGACAGGGATTCTTTTCACT
GTTGTGTCAGTTTATCAAACCCATACTTGGATGAC

```
        10        20          30        40          50
GAAAGACAUGCU     C     --|              GG     -   UU
            GUCCA AGUGU  GUUUGAUAAGCUGACAU  GACAG GGA  \
            UAGGU UCAUA  CAAACUAUUUGACUGUG  UUGUC CUU  C
CAG---------     -     CC^              --     A   UU
              90          80        70          60
```

mml-miR-600

AAGTCACtTaCTGTGtCTCCAGCTTCAcAGGAAGGCTCTTGTCTGTCAGGCAGTGGAGTTACAGACAAGA
GCCTTGCTCAGGCCAGCCCTGCCC

```
20                30        40        50
  CCA--------    CAc  G               CA   AG
             GCUU   AG AAGGCUCUUGUCUGU  GGC  U
             CGGA   UC UUCCGAGAACAGACA  UUG  G
  CCCGUCCCGAC    C--  G               --   AG
     90        80          70          60
```

mml-miR-601

TGCATGAGTTCaTCTTGGTCTAGGATTGTTGGAGGAGTCAGAAAAAtTACCCCAGGGATC
CTGAAGTCaTTGGGgTGGA

```
        10        20            30         40
UGCAUGAG       UG  C----|      G    A  -   CAG
        UUCaUCU  GU      UAGGAUU UUGG GG AGU   \
        AGGUgGG  Ua      GUCCUAG GACC CC UuA   A
--------       GU  CUGAA^      G    -  A   AAA
                70        60         50
```

mml-miR-604

AGAGCATCGTGCTTGACCTTCCACGCTCcCGTGTCCACTAGCAGGCAGGTTTTCTGACAC
gGGCTGCGGgATTCAGGACAGcGCATCAcGGAGA

```
               10        20         30        40
A------  -| A    G     CCU   A   U-        CACU   AG
```

```
       GA GC UUGU CUUGA   UCC CGC  CcCGUGUC    AGC  G
       CU CG GACA GGACU   AgG GCG  GGgCACAG    UUG  C
AGAGGcA  A^ c    -     U--   -   UC        UCUU   GA
   90        80           70         60        50
```

mml-miR-605

CCCTAGCTTGGTTCTAAATCCCAcGGTGCCTTCTCCTTGGGAAAAACAGAGAAGGCACTg
TGgGATTTAGAAcCAAGTTAGG

```
        10        20        30        40
C|                                 CU   GGA
 CCUAGCUUGGUUCUAAAUCCCAcGGUGCCUUCUC  UG   \
 GGAUUGAACcAAGAUUUAGgGUgUCACGGAAGAG  AC   A
-^                                 --   AAA
  80        70        60        50
```

mml-miR-607

TcGCCcAAAGTCACACAGGTTATAGATCTGGATTGGAACCCAGGtAGCCAGACTGCCTGG
GTTtGAATCCAGATCTgTAACcTGTGTGACTTTGG

```
        10        20        30        40
UcGC|                             G            CC
    CcAAAGUCACACAGGUUAUAGAUCUGGAUU GAACCCAGGuAG  \
    GGUUUCAGUGUGUcCAAUgUCUAGACCUAA uUUGGGUCCGUC  A
----^                             G            AG
        90        80        70        60
```

mml-miR-609

TGCTCtGCTtTTCCTAGGGTGTTgCTCTCATCTCTGGTCTATAATGGGgTAAATGTAGAG
ATGAGGGCAACAgCCTAGGAACAGCAGAGGAACC

```
           10        20        30         40
UG---       u        G                 -| C   AA
     CUCuGCU UUCCUAGG UGUUgCUCUCAUCUCUG GU UAU  U
     GAGACGA AAGGAUCC ACAACGGGAGUAGAGAU UA AUg  G
CCAAG       C        g                 G^ A   GG
   90        80        70        60        50
```

mml-miR-611

AAAATGGTGAGAGgGTTaAGGGGAGTTCCcGACGGAGATGCGAGGACCCCTCGGGGTCTG
ACCCACA

```
        10        20          30
AAAAU|  G   GgG  aA    A  U  -  A  G
     GGU AGA   UU  GGGG GU CC cG CG A
     CCA UCU   GG  UCCC CA GG GC GU G
ACAC-^  G   G--  GC    -  -  A  -  A
       60          50           40
```

mml-miR-612

TCtCATCTGGACCCcaCTGGGgAGGGCTTCTGAGCTCCTcAGCACTgGCAGGAGGGGCTC
CAGGGGCCCTCCCTCCATGGCAGCCAGGACAGGACTCTCA

```
               10         20         30          40
------   CA-     ACC   CU-             -         A-| A
      UCu   UCUGG   Cca   GGGgAGGGCUUCU GAGCUCCUc  GC C
      AGG   GGACC   GGU   UCCCUCCCGGGGA CUCGGGGAG  CG U
ACUCUC   ACA     GAC   ACC             C         GA^ g
.        90        80         70         60        50
```

mml-miR-615

CTCGGGAGGGGCGGaAGGGGGGTCCCCGGTGCTCGGATCTCGAGGGTGCTTATTGTTCGG
TCCGAGCCTGGGTCTCCCTCTTCCCCCCAACCCCCC

```
         10        20        30        40
CUC   A-|  C          UC     U         UC  G  UG
   GGG  GGGG GGaAGGGGGG  CCCGG GCUCGGAUC  GA GG  C
   CCC  CCCC CCUUCUCCCU  GGGUC CGAGCCUGG  UU UU  U
CCC   AA^  -          CU     -         C-  G  AU
     90         80        70         60         50
```

mml-miR-616

TTAGGTAATTCCTCCtCTCAAAACCCTcCAaTGACTTCCcTGACATGAcATAGGAAGTCAcTGGAGaGtT
TTGAGCAGAGGAATGACCTGTTTTAAAA

```
               10        20        30        40
U-------     A       CU        C     a        c-| Ac
        UAGGU AUUCCUC  CUCAAAAC CUcCA UGACUUCC  UG  A
        GUCCA UAAGGAG  GAGUUUuG GAGGU ACUGAAGG  AC  U
AAAAUUUU     G       AC        a     c        AU^ AG
       90        80        70        60        50
```

mml-miR-618

TCTTGTTCACAaCCAAACTCTACTTGTCCTTCTGAGTGTgATTACGccCA
TGgAGTAGCTCAGGAGgCAAaCAGGgTTACCCTGTGGATagGTCTGAaaa

```
               10        20        30        40
U-------|         aCCA      AC   U          GUgAU   G  C
        CUUGUUCACA    AACUCU  UUG CCUUCUGAGU     UAC cc \
        GgaUAGGUGU    UUgGGA  AAC gGAGGACUCG     AUG gG A
aaaAGUCU^         CCCA      Ca   -          -----   A  U
.        90        80        70         60
```

mml-miR-619

CGCCCACCTCAGCCTCCCAAAATGCTGGGATTACAGGCATGAGCCACcGC
aGTCGACCATGAtCTGGACATGTTTGTGCCtgGgAtTGTCAGTTTGCAG

```
        10        20         30        40          50
CGCCCACCUCAGCCUCCCAAAAU|    -    A           CA--   Ca    A
                       GCUGG GAUU CAGGCAUGAGC    CcG  GUCG C
                       UGACU UuAg guCCGUGUUUG    GGU  uAGU C
GACGUU-----------------^    G    G           UACA   C-    A
                         90        80        70         60
```

mml-miR-624

AATGCTGTTTCAAGGTAGTACCAGTAtCTTGTGTTCAGTGGAACCAAGGTAAACACAAGa
TAcTGGTATTACCTTGAGATAGCATTaACACCTAAGTG

```
          10        20        30          40
                                     CAG--| G
--AAUGCUGUUUCAAGGUAGUACCAGUAuCUUGUGUU     UG A
  UUACGAUAGAGUUCCAUUAUGGUcAUaGAACACAA     AC A
\                                    AUGGA^ C
       80        70        60        50
```

mml-miR-625

AGGGTAGAGGtataagGGGGGAAAGTTCTgcAGgCCTGTAATTAGATCTC
AGGACTgTAGAACTTTCtCCCTCACCTCTGCCCT

```
        10        20        30        40
|          auaa                  g    UAAUU
AGGGUAGAGGu    gGGGGGAAAGUUCUgcAG CCUG     \
UCCCGUCUCCA    UCCCuCUUUCAAGAUgUC GGAC     A
^          C---                  A    UCUAG
   80           70        60        50
```

mml-miR-626

ACcGATATcTTTGTCTTATTTctGAGCTGAGGgGtTATTTTTATGCAgTC
TaAATGATCTCAGCTGTCcGAAAATGTCTTCAAgTTTAAAGGCTT

```
       10        20        30        40        50
ACcGAUAUcUUU|    AUUUc      GA    u       AU     CUaAA  A
            GUCUU     uGAGCU  GGgG UAUUUUU  GCAgU     UG U
            CGGAA     AUUUgA  CUUC GUAAAAG  UGUCG     AC C
UU----------^    -----      A-    U       cC     -----  U
              90              80        70             60
```

mml-miR-627

TACTTATTACCGGTAGTGAGTCTGTAAGAAAAGAGGAGGTGGTGGTTTTCCTCCTCTTTT
CTTAGAGACTCACTACCAGTAATAAGAAATACTACTA

```
                 10        20        30        40
UA---------|       C            G               UG U
           CUUAUUAC GGUAGUGAGUCU UAAGAAAAGAGGAGG  G G
           GAAUAAUG CCAUCACUCAGA AUUCUUUUCUCCUCC  U G
AUCAUCAUAAA^       A            G               UU U
      90        80        70        60        50
```

mml-miR-628

ATAGCTGTTGTGTCACTTCCTCATGCTGACATATTTACTAGAGGGTAAAATTAATAACCT
TCTAGTAAGAGTGGCAGTCGAAGGGAAGGaCTCAT

```
        10        20         30        40        50
AUAGCUGU| U   A      CA  -   A   A               AAAA
        UG GUC CUUCCU  UG CUG CAU UUUACUAGAGGGU    U
        AC CaG GAAGGG  GC GAC GUG GAAUGAUCUUCCA    U
U-------^ U   -      AA  U   G   A               AUAA
           90         80        70        60
```

mml-miR-631

GTGGAGAGCCTGGTTAGACCTGGCCCAGACCTCAGCTACACAAGCTGATGGACTGAGTCA
GGGGCCACACTCTCC

```
       10        20        30
GU|     CC     AGA      C   A  -      AC
  GGAGAG  UGGUU   CCUGGC CAG CC UCAGCU  \
  CCUCUC  ACCGG   GGACUG GUC GG AGUCGA  A
--^     AC     ---      A   A  U      AC
      70           60        50
```

mml-miR-632

CGCCTCCTgCCGCAGTGCcTGACGGGAGGCGGAGCGGcGAACGAGGCCGT
CGGCCATTTTGTGTCTGCTTCCTGTGGGACGcGGTcGTAGCCGT

```
         10        20        30        40
-  C  CCU     A  G   G|           G  G    CGA    G
 CG CU   gCCGC GU CcU ACGGGAGGCGGA CG cGAA   GGCC \
 GC GA   UGGcG CA GGG UGUCCUUCGUCU GU GUUU   CCGG U
U  C  UGc     -  -   -^           -  -    UA-    C
   90         80          70          60
```

mml-miR-633

AACCTCTCTTAGCCTCTGTTTCTTTAcTGtGGTAGATACTATTAgCCTAA
AATaAGAAGGCTAATAGTATCTACCACAATAAAATTGTTGTGAtGATA

```
        10        20        30        40        50
AACCUCUCUUAGCCUCUGUUUC|   c                     AAAA
                      UUUA UGuGGUAGAUACUAUUAgCCU    \
                      AAAU ACACCAUCUAUGAUAAUCGGA    U
```

```
AUAGuAGUGUUGUUA-------^   A                     AGAa
       90               80        70        60
```

mml-miR-636

TGGCGGCCTGGGCGGGAGCGCGCGGGCGGGGCCGGCCCCGCTGCCTGGAATTAACCCCGC
TGTGCTTGCTCGTCCCGCCtGCAGCCCTAGGCGGCGTCG

```
          10        20        30         40        50
UG--| G       CGGGA  GC           C   CC-   U  CU  AA
    GC GCCUGGG     GC  GCGGGCGGGGC GGC   CGC GC  GG  U
    CG CGGAUCC     CG  CGuCCGCCCUG UCG   GUG CG  CC  U
GCUG^ G       -----  A-           C   UUC   U  CC  AA
        90              80        70        60
```

mml-miR-638

GTaAGCGGGCGCGGCAGGGATCGCGGGCGGGcGGCGGCCTAGGGtGCGGA
GGGCGGACCGGGAATGGCGCtCCcTGCGCCGCCGGCGTAACTGCGGCGCT

```
        10        20        30           40            50
GUaAGCG     G    GG U   G     -| -  C   -   G----   G  GA
       GGCGC GCAG  A CGC GGCGG Gc GG GGC CUA     GGu CG  \
       UCGCG CGUC  U GCG CCGCC CG cC uCG GGU     CCA GC  G
-------     G    AA -   G     G^ U  C   C   AAGGG   G  GG
  100        90         80        70        60
```

mml-miR-639

TGGCCGACGGGGCGCGCGCGGCCgGGAGGGGCGGGGCGGACGCAcAGCCGCGTTTAGTCT
AgCGCaGCGGTcGCGAGCGCTcTgGGtAtCCTGTcCTG

```
                 10        20        30           40
UG---------   GA        G        GGAGGG   --|   -      Ac
           GCC  CGGGGCGC CGCGGCCg      GCG  GGGC GGACGC  A
           uGG  gUcUCGCG GCGcUGGC      CGC  UCUG UUUGCG  G
GUCcUGUCCuA   --        A        Ga----   gA^   A      CC
       90          80        70            60        50
```

mml-miR-640

GTGACCCTGGGCAAGTTCCTGAAGATCAaACACATCAGATCCCTTATCTG
TAAAATGGGCATGATCCAGGAACCTGCCTCTAtGGTTGCCTTGGaG

```
                 10        20        30            40
------- UG   CU--     A       AA     aACA   ----|    CC
       G  ACC    GGGCA GUUCCUG  GAUCA    CAU    CAGAU  \
       C  UGG    UCCGU CAAGGAC  CUAGU    GUA    GUCUA  C
GaGGUUC GU   UAUC     C       --     ACGG   AAAU^    UU
     90        80        70          60        50
```

mml-miR-642

ATCTGAGcTGGGAGGGTCCCTCTCCAAATGTGTCTTGGGGTGGGGGATCA
AGACACATTTGGAGAGGGAACCTCCCAACTCGGCCTC

```
          10        20        30        40
AU--|    C       G                      GGUG
    CUGAG UGGGAGG UCCCUCUCCAAAUGUGUCUUGG    \
    GGCUC ACCCUCC AGGGAGAGGUUUACACAGAACU    G
CUCC^    A       A                      AGGG
      80        70        60        50
```

mml-miR-643

ACCAAcTGATAcgCATTaTCTACgTGAGCTAGAATACAAGTAGTTGGtGTCTTCAGAGAC
ACTTGTATtCTAGCTCAGGTAGATAcTGAATGgAAAA

```
           10        20        30        40        50
```

```
A---|  AcUGAUAcg  U       g                 A   GG  U
    CCA         CA UaUCUAC UGAGCUAGAAUACAAGU GUU  uG C
    gGU         GU AUAGAUG ACUCGAUCuUAUGUUCA CAG  AC U
AAAA^  AA-------  c       G                 -   AG  U
      90               80        70          60
```

mml-miR-644

TTTTaTTTAGTATTcTTCCATCAGTGTTCATAAGGgATGTTGgTCTGTAG
TTTTCTTATAGTGTGGCTTgCTTAGAGCAAAGgTGGTTCCCT

```
        10        20        30        40        50
UUUUaUUUAGUAUUcUU|    AG     A    G  U    gU      UU
                 CCAUC  UGUUC UAAG gA GUUG  CUGUAG  \
                 GGUgG  ACGAG AUUC UU CGGU  GAUAUU  U
UCCCUU-----------^    AA     -    g  -    GU      CU
 90                   80         70         60
```

mml-miR-648

AgCACAGACgCCTCCAAGTGTGCAGGGCACTGaTGGGGGCCaGGGCAGGC
CCAGCcAAAGTGCAGGACCTGGCACTTAGTCGGAgGTGAGGaTG

```
        10            20            30          40
AgCACAGA       ----      G    ----     Ga    --|    aG
        CgCCUCC    AAGUGU CAGG    GCACU  UGG  GGGCC  G
        GUGgAGG    UUCACG GUCC    CGUGA  AcC  CCCGG  G
GUaGGA--       CUGA      -    AGGA     A-    GA^    AC
   90          80         70        60         50
```

mml-miR-649

GcCCTAGCCAAATACTGTATTTTTtATCaACATTTGGTTGAAAAAcATCTgTGTATTAGT
AAACCTGTGTTGTTCAAGAGTCCgCTGTGcTTTGCTG

```
        10        20        30        40
GcCC    C  A   U  --      uAU      UU    GAAAA--|  U
    UAGC AA UAC GU  AUUUUU   CaACAU  GGUU       AcA C
    GUCG UU GUG Cg  UGAGAA   GUUGUG  CCAA       UGU U
----    U  c   U  CC      CUU      U-    AUGAUUA^  g
          90        80        70         60        50
```

mml-miR-650a-1

CAGTGCTGGGaTCTCAGGAGGCAGCGCTCTCAGGACGTCtCCACCATGGtCTGGGCTCTG
CTCCTCCTCACCCTCCTCACTCAGGGCACAGGTGA

```
                   10         20         30        40
CA----     -------    UCUC-|     C    CU-     GA   CuC
      GUGCU       GGGa     AGGAGG AGCG   CUCAG  CGU   \
      CACGG       UCCU     UCCUCC UCGU   GGGUC  GUA   C
AGUGGA     GACUCAC    CCCAC^     -    CUC     uG   CCA
    90        80        70         60        50
```

mml-miR-650a-2

CAGTGCTGGGaTCTCAGGAGGCAGCGCTCTCAGGACtTCtCCACCATGGtCTGGGCTCTG
CTCCTCCTCACCCTCCTCACTCAGGGCACAGGTGA

```
                   10         20          30        40
CA----     -------|   UCUC-     --     C    UCA     UCuC
      GUGCU       GGGa     AGGAG  GCAG GCUC   GGACu    C
      CACGG       UCCU     UCCUC  CGUC CGGG   UCuGG    A
AGUGGA     GACUCAC^   CCCAC     CU     U    ---     UACC
    90        80        70        60           50
```

mml-miR-650b

CAGTGCTGGGGTCTCAGGAGGCAGCGCTCTCgGGACaTCtCCACCATGGCCTGGGaTCTG

CTCCTCtTCACCCTCCTCACTCAGGGCACAGGTGA

```
                      10        20         30        40
CA----     ----------|    CUC      C    C-       A   CuC
      GUGCU          GGGGU   AGGAGG AGCG  UCUCgGG CaU    \
      CACGG          UCCCA   UuCUCC UCGU  aGGGUCC GUA    C
AGUGGA     GACUCACUCC^    C--      -    CU       G   CCA
    90        80        70           60        50
```

mml-miR-650c

CAGTGCTGGGGTgTCAGGAGGCAGCGCTCTCAGTCtCCACCATGGCCTGGGCTCTGCTCC
TCCTCACtCTCCTCACTCAtGGCACgGGTGA

```
                    10        20          30
CA----     --------    U  C     --|   C    UCA   uCC
      GUGCU        GGGG gU AGGAG  GCAG GCUC   GUC   A
      CACGG        CCUC CA UCCUC  CGUC CGGG   CGG   C
AGUGGg     uACUCACU    u  C     CU^   U    UC-   UAC
90        80        70        60        50         40
```

mml-miR-650d

CAGTGCTGGGGTCTCAGGAGaCAGtGCTgTCgGGACGTCtCCACCATGGCCTGGGCTCTG
CTCCTCCTCACCCTtCTCACTCAaGGCACAGG

```
                   10          20        30        40
CA-     ----------     CUC-|    a   u   gU    A   CuC
   GUGCU         GGGGU     AGGAG CAG GCU  CgGG CGU   \
   CACGG         UCCCA     UCCUC GUC CGG  GUCC GUA   C
GGA     aACUCACUCu     CUCC^    -   U   --    G   CCA
 90        80        70          60           50
```

mml-miR-651

AAgCTATCACTGCTTTTTAGaATAAGCTTGACTTTTGTTCAAATAAAAAcGCAAAAGGAA
AGTGTATCtTAAAAGGCAATGACAGTTTAATaTGTTT

```
                 10        20        30        40
---------|    A   C         Ga   A    GA        UCAAA
         AAgCU UCA UGCUUUUUA  AUA GCUU  CUUUUGU     U
         UUUGA AGU ACGGAAAAU  UAU UGAA  GAAAACG     A
UUUGUaUAA^    C   A         uC   G    AG        cAAAA
      90        80        70        60        50
```

mml-miR-652

ACGAATGGCTATGCACTGCACAACCCTAGGAGAGGGTGCCATTCACATAGACTATAATTG
AATGGCGCCACTAGGGTTGTGCAGTGCACAACCTgCAC

```
        10        20        30        40        50
ACGAAU| CUA                  GAGAG          CA  GA
      GG  UGCACUGCACAACCCUAG     GGUGCCAUUCA  UA  \
      CC  ACGUGACGUGUUGGGAUC     CCGCGGUAAGU  AU  C
CACgU-^ AAC                  A----          UA  AU
        90        80        70            60
```

mml-miR-653

TTCATTCCTTCAGTGTTGAAACAATCTCTACTGAACCAGCTTCAAACAAaTTCACTGGAG
TTTGTTTCAATATTGCAAGAATGATAAGATGGAAGC

```
                   10        20        30        40
U-----------|     CUU              U     C    CCAGCUU
            UCAUUC   CAGUGUUGAAACAA CUCUA UGAA       \
            AGUAAG   GUUAUAACUUUGUU GAGGU ACUU       C
CGAAGGUAGAAU^     AAC              U     C    aACAAA
     90        80        70        60        50
```

mml-miR-654

GGGTAAGTGGAAAGATGGTGGGCCGCAGAACATGTGCTGAGTTCGTGCCATATGTCTGCT
GACCATCACCTTTAGAAGCCC

```
        10        20        30          40
|   AAGUGG    A      GCC     A      - -  A
GGGU      AAAG UGGUGG   GCAGA CAUGUG C UG G
CCCG      UUUC ACUACC   CGUCU GUAUAC G GC U
^   AAGA--    C      AGU     -      C U  U
80          70        60         50
```

mml-miR-656

CTGAAATAGGTTGtCTGTGAGGTGTTCACTTTCTATATGATGAATATTATACAGTCAACC
TCTTTCCGATATCGAATC

```
                  10        20        30
CU----------|   U      u     AG       CUU  UA
            GAAA AGGUUG CUGUG  GUGUUCA   UC  \
            CUUU UCCAAC GACAU  UAUAAGU   AG  U
CUAAGCUAUAGC^   C      U     AU       ---  UA
       70        60        50           40
```

mml-miR-657

GGAGGAGAGGGTCCTGGAGAAGCGTGGACgGcTCCaGGTGGGTTCtGGCA
GGTcCTCACCCTCTCTAGGCCCCATTCTC

```
        10        20        30          40
G|    GA   U        A C    AC     --     UG
 GAGGA  GGG CCUGGAGA G GUGG  gGcU  CCaGG  \
 CUCUU  CCC GGAUCUCU C CACU  cUGG  GGuCU  G
-^    AC   -        - C    C-    AC     UG
         70          60          50
```

mml-miR-660

CTGCTCCTTCTCCCATACCCATTGCATATCGGAGTTGTaAATTCTCAAAACACCTCCTGT
GTGCATGGATTACAGGAGGGTGAGCCTTGTCATCGTG

```
                  10         20        30        40
CU----------    C      CAUAC-|   U      C    U   aAAUU
            GCUC UUCUCC      CCAU GCAUAU GGAG UGU     \
            CGAG GGGAGG      GGUA CGUGUG CCUC ACA     C
GUGCUACUGUUC    U      ACAUUA^   -      U    C   AAACU
      90        80        70         60        50
```

mml-miR-661

GGAGAGGCTGTGCTGTGGGGCAGGCGCtggcctgggtGGCCTGAGCCCTG
aTTTtGGGCTGCCTGGGTaTCTGGCCcGtGCGTGACcTTGGGGcGGCT

```
        10        20         30        40          50
GGAGA|     G  GU     -  G    u    u     G  UG--     UGa
     GGCUGU CU  GGGG CA GCGC ggcc ggguG CC    AGCCC   \
     UCGGcG GG  UUcC GU CGuG CCGG UCUaU GG    UCGGG   U
-----^     G  --     A  G    c    -     G  UCCG     uUU
            90          80         70         60
```

mml-miR-662

GCTGTTGAGGCTGtaCAGCCAGGaCCTGACGGTGGGGTGGCTtCGGGCCTTCTGcAGGTC
TCCCACGTTGTGGCCCAGCAGCGCAGTCACGTTGC

```
                     10        20            30        40
-------------       A    Gu     CA---      A|  U     GGC
             GCUGUUG GGCU  aCAGC     GGaCCUG CGG GGGGU   U
             CGACGAC CCGG  UGUUG     UCUGGAc GUC UUCCG   u
CGUUGCACUGACG       -    --     CACCC      -^  -     GGC
```

```
    90        80           70        60          50
```

mml-miR-663

CCgTtCGGCGTCCCAGGCGGGGCGCtGCGGGACCGCCCTCGTGTCTGTGGCGGTGGGATC
CCGtGGCCGTGTTTTCCTGGTGGCCCGGCC

```
        10          20         30          40
CCgU    C   -     C-  G  -  uG     --|    C   UG
    uCGG GUC CCAGG  GG GC GC  CGGGA  CCGCC UCG  U
    GGCC CGG GGUCC  UU UG CG  GCCCU  GGUGG GGU  C
CC--    -   U     UU  G  C  Gu     AG^    C   GU
.           80        70        60        50
```

mml-miR-664

CTGGCTAGGGAAAATGATTGGATAGAAAaTgTTATTCTATTCATTTATCCCCAGCCTA

```
        10        20        30
CU|   A    AAA   U         AaU
  GGCU GGGA   UGA UGGAUAGAA   \
  CCGA CCCU   AUU ACUUAUCUU   g
AU^   C    ---   U         AUU
       50           40
```

mml-miR-668

GGTAAGTGCGCCTCGGGTGAGCATGCACTTAATGTGGGTGTATGTCACTC
GGCTCGGCCCACTACC

```
       10           20        30
   A    C   ---|       G         AA
GGU AGUG GCC   UCGGGUGA CAUGCACUU  \
CCA UCAC CGG   GGCUCACU GUAUGUGGG  U
   -    C   CUC^       -         UG
      60        50         40
```

mml-miR-671

GCAGGTGAACTGGCAGGCCAGGAAGAGGAGGAAGCCCTGGAGGGGCTGGAGGTGATGGAT
GTTTTCCTCCGGTTCTCAGGGCTCCACCTCTTTCGGGCCGTAGAGCCAGGGCTGGTGC

```
        10            20        30        40        50
   GGUGAA     A----|   A        A   A      GA           U-   G
GCA      CUGGC     GGCC GGAAGAGG GGA GCCCUG  GGGGCUGGAGG  GAU G
CGU      GACCG     CCGG CUUUCUCC CCU CGGGAC  UCUUGGCCUCC  UUG A
   GGUCGG     AGAUG^   G        A   -      --           UU   U
      110       100        90         80          70         60
```

mml-miR-675

CCCAGGGTCTGGTGCGGAGAGGGCCCACAGTGGACTTGGTGACaCTGTATGCCCTCACCG
CTCAGCCCCTGGG

```
        10        20        30
|      U    U    A      CC      GACU
CCCAGGG CUGG GCGG GAGGGC  ACAGUG    U
GGGUCCC GACU CGCC CUCCCG  UGUCaC    G
^      C    -    A      UA      AGUG
  70         60        50        40
```

mml-miR-758

GCCTGGATACgTGAGATGGTTGACCAGAGAGCACACGCTTTATaTGTGCCGTTTGTGACC
TGGTCCACTAcCCCTCAGTATCTAATGC

```
         10          20         30          40
GCC-       g    A--    U      AGA   C  -| UUUA
    UGGAUAC UGAG   UGGU GACCAG   GCA AC GC    \
```

```
    AUCUAUG ACUC   AUCA CUGGUC   UGU UG CG    U
CGUA       -    CCc    C      CAG    U  C^ UGUa
       80         70        60        50
```

mml-miR-765

TTTAGGgGCTGATGAAAGTGGAGTTCAGTAGACAaCCCTTTTCAAGCCCTgCaAGAAACT
GGGGTTTCTGGAGGAGAgGGAAGGTGcTGAAGGggCTGcTCTCGTGAGCCTGAA

```
        10        20          30        40             50
UUUAG     G    A  UG AG--       GACAa        -----|       CaA
     GgGCU AUGA AG  G    UUCAGUA     CCCUUUUC     AAGCCCUg   G
     UCCGA UGCU Uc  C    AAGUcGU     GGgAGAGG     UUUGGGGU   A
AAG--     G    C  GU ggGG       GGAA-        AGGUC^       CAA
    110       100        90         80        70        60
```

mml-miR-767

GCTTTTATATTGTAGGTTTTTGCTCATGCACCATGGTTGTCTGAGCATGCAGCATGCTTG
TCTGCTCATACCCCATGGTTTCTGAGCAGGAAtCTTCATTGTCTACTGCt

```
          10        20        30        40        50
-  UUUU-|  U  U             UGC       UU  C      U    CA
 GC      AUA UG AGGUUUUUGCUCA   ACCAUGG  GU UGAGCA GCAG  \
 CG      UGU AC UCuAAGGACGAGU   UGGUACC  CA ACUCGU UGUU  U
u  UCAUC^  U  U             CUU       C-  U      C    CG
.       100        90        80          70        60
```

mml-miR-768

CTGTGCTTTGTGTGTTGGAGGATGAAAGTACGGAGTGATCCATCGGCTAAGTGTCTTaTC
ACAATGCTGACACTCAAACTGCTGACAGCACACGTTTTTCACAG

```
          10        20        30        40        50
     CUU--|        GA  AUGAA   ACG     AUCCA     UAA   UC
CUGUG     UGUGUGUUG  GG     AGU   GAGUG     UCGGC   GUG  U
GACAC     GCACACGAC  UC     UCA   CUCAC     AGUCG   CAC  U
     UUUUU^        AG  G----   AA-     -----     UAA   Ua
  100        90            80              70        60
```

mml-miR-770

AGGAGCCACCTTCCGAGCCTCCAGTACCACGTGTCAGGGCCACATGAGCTGGGCCTCGTG
GGCCTGATGTGGTGCTGGGGCCTCAGGGGTCTGCTCTT

```
         10        20        30        40        50
      C-|    CC   C            GU      G  A      CU
AGGAGC  ACCUU  GAG CUCCAGUACCAC  GUCAGG CC CAUGAG  G
UUCUCG  UGGGG  CUC GGGGUCGUGGUG  UAGUCC GG GUGCUC  G
      UC^    A-   C            --      -  -      CG
       90         80        70            60
```

mml-miR-802

GTTCTGTTATTTGCAaTCAGTAACAAAGATTCATCCTTGTGTCCATCATGCAgCAAGGAG
AATCTTTGTCACTTAGTGTAATTAATAGCTGGAC

```
           10        20        30         40
GUU---|     U     aUC   A          A      -   CCA
      CUGUUA UUGCA   AGU ACAAAGAUUC UCCUUG UGU   \
      GAUAAU AAUGU   UCA UGUUUCUAAG AGGAAC ACG   U
CAGGUC^     U     GAU   C          -      g   UAC
   90        80        70        60         50
```

mml-miR-874

TTAGCCCTGCGGCCCCACGCACCAGGGTAAGAGAGAgTCTCGCTTCCTGC
CCTGGCCCGAGGGACCGACTGGCTGGGC

```
             10        20        30
UUA|   UG-----   C   A  CA        A   A-  g
   GCCC       CGG CCC CG  CCAGGGUA GAG  GA \
   CGGG       GCC GGG GC  GGUCCCGU CUU  CU U
---^   UCGGUCA   A   A  CC        C   CG  C
          70        60        50        40
```

mml-miR-875-5p

TTAGTGGTACTATACCTCAGTTTTATCAGGTGTTCcTAAAATCACCTGGA
AAtACTGAGGTTGTGTCTCACTGAAC

```
          10        20        30
--       U  UAU        U  A       -| Cc
  UUAGUGG AC   ACCUCAGU UU UCAGGUG UU  \
  AGUCACU UG   UGGAGUCA AA GGUCCAC AA  U
CA       C  UGU        u  A       U^ AA
     70        60        50        40
```

mml-miR-875-3p

```
          10        20        30
--       U  UAU        U  A       -| Cc
  UUAGUGG AC   ACCUCAGU UU UCAGGUG UU  \
  AGUCACU UG   UGGAGUCA AA GGUCCAC AA  U
CA       C  UGU        u  A       U^ AA
     70        60        50        40
```

mml-miR-876-5p

TGAAGTGCTGTGGATTTCTTTGTGAATCACCATATCTAAGCTAATGTGGT
GGTGGTTTACAAAGTAATTCATAGTGCTTCA

```
        10        20        30        40
|               U                AUCUAAG
UGAAGUGCUGUGGAUU CUUUGUGAAUCACCAU       \
ACUUCGUGAUACUUAA GAAACAUUUGGUGGUG       C
^               U                GUGUAAU
80        70        60        50
```

mml-miR-876-3p

```
        10        20        30        40
|               U                AUCUAAG
UGAAGUGCUGUGGAUU CUUUGUGAAUCACCAU       \
ACUUCGUGAUACUUAA GAAACAUUUGGUGGUG       C
^               U                GUGUAAU
80        70        60        50
```

mml-miR-877

GCTAGAGAAGGTAGAGGAGATGGCGCAGGGGACACGGGCtAAGACTcGGG
GGTTCCTGGGACCCTCAGACaTGTGTCCTCTTCTCCCTCCTCCCAGGTGT
ATG

```
             10        20        30         40        50
-----|  AGAGA   U  A     UGGCGC          GG-  AAGACUc        C
     GCU     AGG AG GGAGA      AGGGGACACG   Cu       GGGGGUUC \
     UGG     UCC UC CCUCU      UCUCCUGUGU   GA       CUCCCAGG U
GUAUG^  ACCC-   -  -     ------          aCA  -------        G
 100         90                 80        70                60
```

mml-miR-877*

```
            10        20        30          40        50
-----|  AGAGA   U  A     UGGCGC          GG-  AAGACUc        C
     GCU     AGG AG GGAGA      AGGGGACACG   Cu       GGGGGUUC \
     UGG     UCC UC CCUCU      UCUCCUGUGU   GA       CUCCCAGG U
GUAUG^  ACCC-   -  -     ------          aCA  -------        G
 100         90                80        70               60
```

mml-miR-885-5p

CCGCACTCTCTCCATTACACTACCCTGCCTCTTCTCCATGAGAGGCAGCG
GGGTGTAGTGGATAGAGCACGGGT

```
          10        20         30
--|  CA    C           A  -         CU
  CCG  CUCU UCCAUUACACU CC CUGCCUCUU  C
  GGC  GAGA AGGUGAUGUGG GG GACGGAGAG  C
UG^  AC    U           -  C         UA
   70        60         50        40
```

mml-miR-885-3p

```
          10        20         30
--|  CA    C           A  -         CU
  CCG  CUCU UCCAUUACACU CC CUGCCUCUU  C
  GGC  GAGA AGGUGAUGUGG GG GACGGAGAG  C
UG^  AC    U           -  C         UA
   70        60         50        40
```

mml-miR-886-5p

CCGGGTCGGAGTTAGCTCAAGCGGTTACCTCCTCATGCCGcACTTTCTAaCTGTCCATCTCTGT
GCTGGGGTTCGAGACCCGCGGGTGCTTACTGACCCTTTTATGCAcTAA

```
                   10         20        30          40        50
CC-----------|      AGUU   -  AA     UUACCU--     UGCC     UUUCUAaCU
             GGGUCGG    AGC UC  GCGG         CCUCA    GcAC         \
             CCCAGUC    UCG GG  CGCC         GGGGU    CGUG         G
AAUcACGUAUUUU^      AU--   U  G-     CAGAGCUU     ----     UCUCUACCU
110       100          90         80        70            60
```

mml-miR-866-3p

```
                   10         20        30          40        50
CC-----------|      AGUU   -  AA     UUACCU--     UGCC     UUUCUAaCU
             GGGUCGG    AGC UC  GCGG         CCUCA    GcAC         \
             CCCAGUC    UCG GG  CGCC         GGGGU    CGUG         G
AAUcACGUAUUUU^      AU--   U  G-     CAGAGCUU     ----     UCUCUACCU
110       100          90         80        70            60
```

mml-miR-887

TGCAGATCCTTGGGAGCCCTGTTAGACTCTGGATTTTACACTTGGAGTGA
ACGGGCGCCATCCCGAGGCTTTGCACAG

```
           10           20        30
---      U        --  -|     AG        AUUU
   UGCAGA CCUUGGGA  GC CCUGUU  ACUCUGG    \
   ACGUUU GGAGCCCU  CG GGGCAA  UGAGGUU    U
GAC      C        AC  C^     G-        CACA
       70        60        50         40
```

mml-miR-888

GGCAGTGCcCTACTCAAAAAGCTGTCAGTCACTTAtgTTACATGTGACTG
ACACCTCTTTaGaTGAAGGAAGGCTCA

```
       10             20        30
GGCAGU   CUAC-|  ----  A  C          UUAug
      GCc     UCA    AA AG UGUCAGUCAC     \
      CGG     AGU    UU UC ACAGUCAGUG     U
ACU---   AAGGA^  aGaU  C  C          UACAU
         70        60        50        40
```

mml-miR-888*

```
        10              20        30
GGCAGU   CUAC-|  ----  A  C          UUAug
      GCc     UCA    AA AG UGUCAGUCAC     \
      CGG     AGU    UU UC ACAGUCAGUG     U
ACU---   AAGGA^  aGaU  C  C          UACAU
         70        60        50        40
```

mml-miR-889

GTGCTTAAAGAATGGCTGTCCGTAGTATGGTCTCTATATTTATGATGATT
AATATCGGACAACCATTGTTTTAGTATCC

```
          10        20        30
--|    U   G     C      UAGUA     UC   A
  GUGCU AAA AAUGG UGUCCG     UGGUC  UAU U
  UAUGA UUU UUACC ACAGGC     AUUAG  GUA U
CC^    U   G     A      UAUA-     UA   U
        70        60         50        40
```

mml-miR-890

```
               10        20        30
-------|  C             CAc       UUAGA
       UGC CUACUUGGAAAGG   CAGUUaC     \
       AUG GAUGAGUCUUUCC   gUCAAUG     U
AUUCgGA^  A             CUU       cACAU
 70        60        50        40
```

mml-miR-891

CCTTAATCCTTGCAACTTACCTGAGTCATTGATTCAGTAAAACATTCAAT
GGCACATGTTTGTTGTTAGGGTCAAAAGA

```
          10        20        30
CC--| A     U     UU  C  A        UUCAGU
    UU AUCCU GCAAC  AC UG GUCAUUGA      \
    AA UGGGA UGUUG  UG AC CGGUAACU      A
AGAA^ C     U     UU  U  A        UACAAA
        70        60        50        40
```

mml-miR-892

GCAGTGCtcTACTtAGAAAGGTGCCAGTCACTTACAtTACATGTCACTGT
GTCCTTTCTGCGTAGAGTAAGGCTC

```
          10        20         30
--| AG        U        U  -    C  UUACA
  GC  UGCucUAC uAGAAAGG GC CAGU AC     \
  CG  AUGAGAUG GUCUUUCC UG GUCA UG     u
CU^ GA        C        -  U    C  UACAU
    70        60         50        40
```

mml-miR-920

GTAGTTGTTC TgCAGAAGAC CTGGATGTGg AaGAGCTAAG ACACACTCCA
GGGGAGCTGT aGAAGCgGTA ACACG

```
            10        20         30
---  A U  -        AAGA      -|   gAaGA
   GU G UG UUCUgCAG    CCUGGA UGUG     G
   CA U gC AAGaUGUC    GGACCU ACAC     C
GCA  A G  G        GAGG      C^   AGAAU
    70        60        50        40
```

mml-miR-922

TGGCGTTcTCtCTCTCCCTGTCCTGGACTGGGGTCAGACcGTGCCCCGAG
GAGAAGCAGCAGAGAATgaGACTACGTCgT

```
           10          20             30
-      --    uC    C-   ----|   GGAC     U  GA
 UGGCGU  UcUC  UCUC  CUG    UCCU    UGGGG CA  \
 gCUGCA  AGag  AGAG  GAC    AGGA    GCCCC GU  C
U      UC    UA    AC    GAAG^   ----     -  Gc
.        70        60        50             40
```

mml-miR-924

AATAGAGTCTTGTGtTGTCTTGCTTAAaGGCCATCCAACCTAGAGTCTA

```
        10            20
AA    G   ----| U  U     GC
  UAGA UCU    UG Gu GUCUU  \
  AUCU AGA    AC UA CGGaA  U
--    G   UCCA^ C  C     AU
         40        30
```

mml-miR-933

CTTGGGTCAGTTCAGAGGTCCTCGGGGCGCGCGTCGAGTCAGCCGTGTGC
GCAGGGAGACCTCTCCCACCCACAGT

```
          10        20         30
CU--|    CAGUUC      -   GGG       U  AGU
    UGGGU      AGAGGUC CUC   GCGCGCG CG   \
    ACCCA      UCUCCAG GGG   CGCGUGU GC   C
UGAC^    CCC---      A   A--       -  CGA
     70            60          50
```

mml-miR-934

AGgAATAAGGCTTCTGTCTACTACTGGAGACACTGaTAGTgTAAAACCCA
GAGTCTTtCgGTAATGGACGGGAGCCTTATTTCT

```
        10        20        30        40
                    C          A   aUA  gU
AGgAAUAAGGCUUCUGUCUA UACUGGAGAC CUG   GU  \
UCUUUAUUCCGAGGGCAGGU AUGgCuUCUG GAC   CA  A
^                   A          A   C--  AA
  80        70        60        50
```

mml-miR-936

TCAAGGaCACTGGGACAGgAGAGGGAGGAATCGCAGAAATCACTCCAGGA
GCAACTGAGAGACCTTGCTTCTACTTTACCAGGTCCTGCTGGCCCAGA

```
         10         20        30         40         50
UCAA  a-  C      A-  A    GA    UC|   AAAUCA   -   GA
    GG  CA UGGGAC  Gg GAGG  GGAA  GCAG      CUC CAG  G
    CC  GU GUCCUG  CC UUUC  UCUU  CGUU      GAG GUC  C
AGA-  CG  C      GA  A    A-    --^   CCA---   A   AA
        90        80         70             60
```

mml-miR-937

AGCACTGCCCCCGGTGAGTCAGGGTGGGGCTGGCCCCCTGCTTCGcGCCC
ATCCGCaCTCTGACTCTCcaCCtgCCTGCAGGAGCT

```
         10            20             30
   A-     CCCC-   ---           -----    U---|  CC
AGC  CUGC     GGU   GAGUCAGGGUG     GGGC    GGC  \
UCG  GACG     CCa   CUCAGUCUCaC     CCCG    UCG  C
   AG     UCCgu   cCU           GCCUA    cGCU^  UC
     80        70        60        50        40
```

mml-miR-938

GAAaGTGTACCATGTGCaCTTAAAGaTGAAgCCgGTGCACCTTCATGAAC
tGTGGTACACCTTTAAGAACTTGGT

```
        10        20        30          40
   a   U    U  GCa       a  AA   --|  G  CC
GAA GUG ACCA GU   CUUAAAG UG  gCC  gGU CA  U
cuu cac UGGU CA   GAAUUUC AC  UGG  uCA GU  U
   c   c    U  A--       C  A-   UG^  A  AC
  80         70         60         50
```

mml-miR-939

TGTGGGCAGGGCCCTGGGGAGCTGAGGCTCTGGGGGTGGCCGGGGCTGAc
CCCTGGGCCTCTGCTCCCCAGTGTCTGACCGtG

```
        10        20        30         40
UG|  GCA    C         U     U        -   C
  UGG   GGGC CUGGGGAGC GAGGC CUGGGGU  GGC G
  GCC   UCUG GACCCCUCG CUCCG GGUCCCcA UCG G
Gu^  AG-    U         U     -        G   G
  80         70        60         50
```

mml-miR-940

GTGgGGTGTGGGCCCGGCCCCAGGAGCGGGGCCTGGGCAGCCCCGTGTGT
TGAGGAAGGAAGGCAGGGCCCCCGCTCCCCGGGCCTGACCCCAC

```
        10        20        30         40
|      G         CCCCA         -   GG  AG-  --  GUGU
GUGgGGU UGGGCCCGG     GGAGCGGGG CCU  GC   CC  CC    \
CACCCCA GUCCGGGCC     CCUCGCCCC GGG  CG   GG  GG    G
^      -         -----         C   A-  GAA  AA  AGUU
   90         80             70         60         50
```

mml-miR-942

ATTAaGAGAGTAcCTTCTCTGTTTTGGCCATGTGTGTACTCACAGCCCCT
CACACgTGGCCGAAACAGAGAAGgTACTTTCCTAAT

```
        10        20        30            40
    a                              UA---| C
AUUA GAGAGUAcCUUCUCUGUUUUGGCCAUGUGUG     CU \
UAAU CUUUCAUgGAAGAGACAAAGCCGGUgCACAC     GA A
    C                              UCCCC^ C
     80        70        60        50
```

mml-miR-944

GTTCCAGACACATCTCATCTGATATACAATATTTTCTTAAATTGTAaAAA
GAGAAATTATTGTAtATCaGATGAGaTGTGTCTGGGgT

```
        10        20        30         40
G|                             U      AAA  G
 UUCCAGACACAUCUCAUCUGAUAUACAAUA UUUCUU   UU U
 gGGGUCUGUGUaGAGUAGaCUAuAUGUUAU AAAGAG   AA A
U^                             U      A--  a
```

80 70 60 50
